# Borane-Pyridine: An Efficient Catalyst for Direct Amidation

**DOI:** 10.3390/molecules29010268

**Published:** 2024-01-04

**Authors:** P. Veeraraghavan Ramachandran, Aman Singh, Harry Walker, Henry J. Hamann

**Affiliations:** Department of Chemistry, Purdue University, 560 Oval Drive, West Lafayette, IN 47907, USAhjhamann@purdue.edu (H.J.H.)

**Keywords:** direct amidation, carboxamides, borane-pyridine, borane-amines, liquid catalyst, catalysis

## Abstract

Borane-pyridine acts as an efficient (5 mol%) liquid catalyst, providing improved solubility for the direct amidation of a wide range of aromatic and aliphatic carboxylic acids and amines to form secondary and tertiary carboxamides. Tolerance of potentially incompatible halo, nitro, and alkene functionalities has been demonstrated.

## 1. Introduction

Amide functionality plays an undeniably important role in biochemistry and pharmaceutical chemistry [1]. An appreciable number of major medicines and natural products contain amide bonds [2]. The challenge of amide formation is still a pressing objective as their synthesis is still one of the most frequently used and practiced transformations in process and medicinal chemistry [3]. A variety of techniques have been implemented to increase the reactivity of carboxylic acids to enhance amidation such as activation via acid chlorides, anhydrides, imidazolides, carbodiimides, thioesters and other coupling reagents [2]. Silane reagents have been documented recently as efficient amidating tools with tetramethylorthosilicate (TMOS) and methyltrimethoxysilane (MTM) having been explored for this purpose [4]. Boron-based amidation reagents, though known for some time, have recently been taken up again and studied extensively for direct amidation [5]. Many of the boron-based reagents are catalytic, avoiding one of the most obvious drawbacks with stoichiometric reagents: the large quantity of waste produced.

**Scheme 1:**
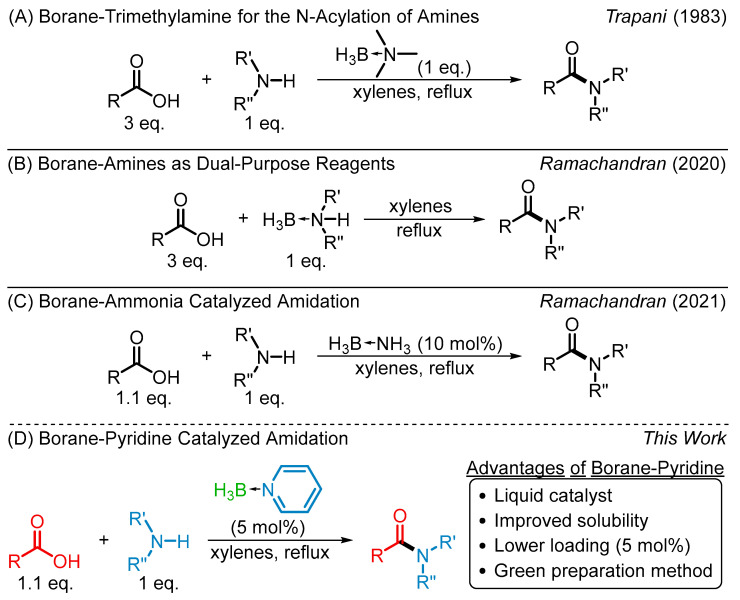
Prior and current borane-amine amidations. Prior work (**A**) N-Acylation of amines promoted by borane-trimethylamine [14], (**B**) Borane-amines as dual purpose reagents for amide formation [17], and (**C**) Borane-ammonia as a catalyst for direct amidation [18]; Current work (**D**) Borane-pyridine as a catalyst for direct amidation.

Pioneering work in boronic acid catalyzed direct amidation was performed by Yamamoto, with additional developments and improvements made by Blanchet [6], Hall [7], Whiting [8], and more recently Xian-Bin [9] as well as Liu, who utilized boronic acids as starting materials for amide synthesis [10]. Boronic ester and boric acid mediated reactions have also been explored, with Sheppard making the use of tris(2,2,2-trifluoro) borate and other simple borate esters [11] and Tang utilizing boric acid [12]. Triacyloxyborane intermediates formed through the reaction between a carboxylic acid and borane-tetrahydrofuran [13] or borane-trimethylamine [14] (Scheme 1A) were also found to be sufficiently activated to enable amidation.

Our recent focus and success in exploring borane-amines in a wide variety of reactions led us to further investigate their application in carboxamide synthesis. While borane-amines have been demonstrated to be viable reductants for various functional groups, including carboxylic acids [15] and amides [16], these reactions require a catalyst to proceed. The potentially competing reduction was not found to be an issue in our recently reported direct amidation of carboxylic acids and borane-amines, wherein the amine coordinated to boron was incorporated into the product amide (Scheme 1B) [17]. Our subsequent report on the use borane-ammonia (BH_3_NH_3_, **1a**) as an efficient sub-stoichiometric amidation catalyst was found to require 10 mol% of **1a** (Scheme 1C) [18]. Catalytic application of borane-ammonia achieved fantastic results in direct amidation of various aryl and alkyl carboxylic acids and amines, delivering appreciable yields of a variety of carboxamides.

However, several issues with the use of **1a** soon became apparent, especially with the transition to continuous flow synthesis in mind. The static nature of solid **1a** makes handling cumbersome. The low solubility of **1a** in the reaction solvent additionally makes the potential advancement from batch to continuous flow synthesis difficult. Herein, we report the use of pyridine-borane (5 mol%) as an effective and efficient liquid amidating reagent for the synthesis of various aryl and alkyl carboxamides in good to excellent yields (Scheme 1D).

## 2. Results and Discussion

As a part of the project utilizing catalytic **1a**, borane-trimethylamine (**1i**) and triethylamine borane (**1j**) were tested and provided acceptable yields. The optimization studies carried out yielded model amide **4a** in 93% and 91% yields with **1i** and **1j** respectively. Since **1a** produced a higher yield, it was ultimately chosen as the preferred catalyst at that time.

**Scheme 2:**
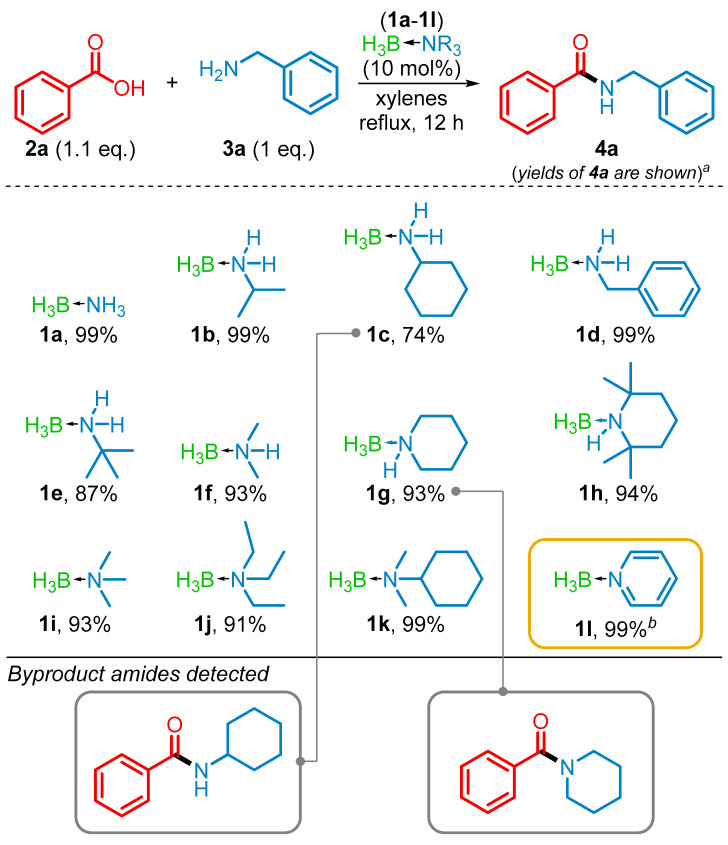
Scope of examined borane-amine catalysts. *^a^* Isolated yields are shown. *^b^* Yield obtained when using 5 mol%.

To overcome the identified challenges posed by the use of **1a**, our focus shifted to exploring alternate borane-amines for direct amidation. Thus, a variety of 1° (isopropylamine (**1b**), cyclohexylamine (**1c**), benzylamine (**1d**), *t*-butylamine (**1e**)), 2° (dimethylamine (**1f**), piperidine (**1g**), 2,2,6,6-tetramethylpiperidine (**1h**)), 3° (trimethylamine (**1i**), triethylamine (**1j**), *N,N*-dimethylcyclohexylamine (**1k**)), and heteroaromatic (pyridine (**1l**)) borane-amines were examined (Scheme 2). Each of the borane-amines tested provided good to excellent yield of the model amide **4a**. However, the use of borane coordinated to 1° and 2° amines (**1b**–**1h**) was eliminated due to the detection (in the ^1^H NMR spectra of the product) of a second byproduct amide formed from the borane-coordinated amine and carboxylic acid. The lower yields obtained when using **1i** and **1j**, and the drawbacks associated with **1k**, including cost and difficulty in separation from the reaction mixture due to high boiling point and low water solubility, led to the selection of borane-pyridine (**1l**) as the optimal catalyst. Additional benefits of **1l** include the fact that it is a liquid, it works at a lower (5 mol%) catalyst loading, and our recently described large-scale, green synthesis of **1l** eliminates the use of tetrahydrofuran from its preparation [19].

Standardization of conditions led to the use of 1 eq. of amine and 1.1 eq. of carboxylic acid, similar to previous protocol [18]. Completion time for the majority of the examples is 12 h, although some required only 6 h. Xylenes proved to be the appropriate solvent for pyridine-borane **1l** catalyzed amidation, while a comparatively lower boiling point solvent such as toluene resulted in a decreased yield of 75% of **4a**, proving the necessity for higher temperatures. While 5 mol% of **1l** is adequate to carry forth the transformations, some substrates require an increased loading of 10 mol% to improve carboxamide yield.

Loading of a lower sub-stoichiometric value of **1l** was also tested. Decreasing the amount of pyridine-borane **1l** from 5 mol% to 1 mol% yielded **4a** in 98%, but when similar conditions were analyzed against **4j**, only 54% of the product could be isolated. Further decreasing the loading to 0.1 mol% isolated the carboxamide in 69% yield. Since the latter sub-stoichiometric measurements were only effective for one substrate, 5 mol% was determined as the ideal loading for further analysis. A 100 mmol scale was additionally performed for this substrate (**4a**), where a slightly decreased yield of 67% (14.2 g) was obtained, likely due to a buildup of byproduct water too much for azeotropic removal during the course of the reaction.

**Scheme 3:**
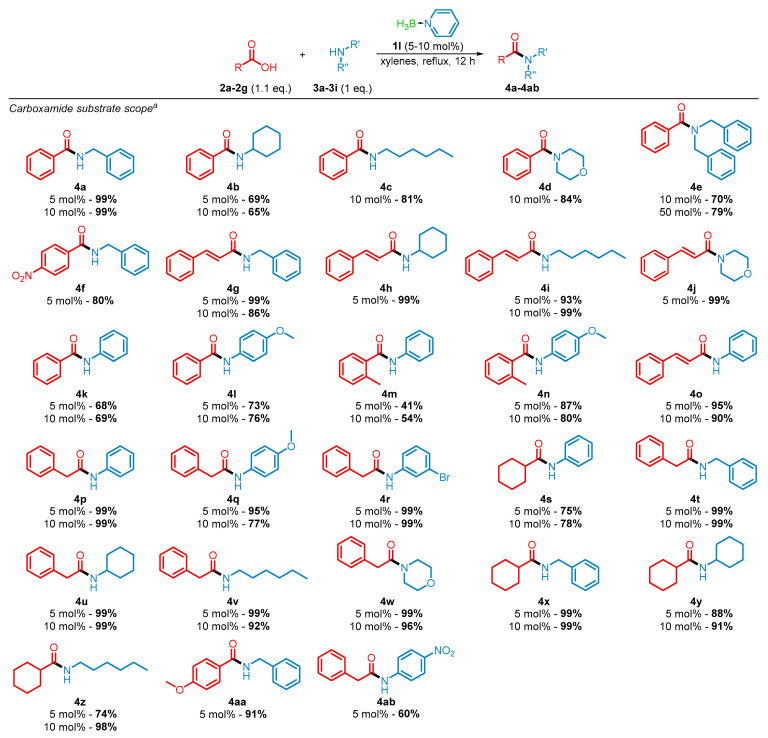
Scope of carboxamides synthesized using borane-pyridine (**1l**). *^a^* Isolated yields are shown.

The substrate scope analysis for carboxamide synthesis using **1l** was performed utilizing a variety of aromatic and aliphatic starting materials (Scheme 3). Aromatic carboxylic acids were initially studied with aliphatic amines. Cyclohexylamine **3b** underwent amidation with benzoic acid (**2a**) to give the product (**4b**) in 69% yield. In contrast, when **1a** was used, the reaction produced carboxamide **4b** in 98% yield, proving the previous method to be more efficient for the synthesis of this particular carboxamide [18].

For the tertiary carboxamide **4d**, 5 mol% of **1l** was insufficient and 10 mol% was used to produce a yield of 84%. However, amidation of the bulky dibenzylamine (**3e**) with **2a** required 50 mol% of **1l** to give an isolated yield of 79%, likely due to the hindered nature of the amine. This finding was in agreement with results of **1a** [18]. Interestingly, when p-nitrobenzoic acid (**2b**) was reacted with benzylamine (**3a**), borane-pyridine **1l** produced **4f** in 80% yield, which in comparison only yielded 69% when **1a** was used [18], showing the present protocol to be superior for carboxylic acid substrates bearing electron-withdrawing substituents. Cinnamic acid (**2c**) showed excellent reactivity with amines **3a** and **3b**, hexylamine (**3c**), and morpholine (**3d**) with quantitative yields of **4g**, **4h**, **4i**, and **4j** in the presence of 5 mol% **1l**.

In the amidation scope of carboxamides bearing an aromatic acid and an aromatic amine, aniline (**3f**) yielded 68% in 12 h when it reacted with **2a**. On increasing the loading of **1l** to 10 mol%, the carboxamide **4k** was isolated in a similar yield after 6 h of reflux in xylenes. Reflux for 12 h resulted in decomposition. Keeping the aromatic acid (**2a**) as constant, *p*-anisidine (**3g**) yielded the corresponding amide (**4l**) in 73% yield with 5 mol% **1l** and similar results were obtained when testing 10 mol%. o-Toluic acid (**2d**) reacted with **3f** and **3g** to produce 41% and 87% yields of carboxamides **4m** and **4n** respectively. However, when **2d** was treated with **3f** using 10 mol% of **1l**, the yield increased to 54%. Cinnamic acid (**2c**) and aniline (**3f**) provided the isolated product **4o** at 95% yield using 5 mol% of **1l**.

Phenylacetic acid (**2e**) gave similar satisfactory results with aromatic amines **3f**, **3g**, and **3h** as well as aliphatic amines **3a**, **3b**, **3c**, and **3d** with isolated yields >95% obtained for carboxamides **4p**–**4r** and **4t**–**4w**. The reaction of cyclohexanecarboxylic acid (**2f**) with aniline (**3f**) gave an improved yield (78%) of **4s** when **1l** was increased to 10 mol%. In fact, this trend was also observed in the reactions of **2f** with amines **3a**, **3b**, and **3c** with isolated yields in the range of 91–99% for carboxamides **4x**–**4z**.

To examine the tolerance of strongly electron-donating or electron-withdrawing groups, an aromatic acid with a strongly electron-donating methoxy group, 4-methoxybenzoic acid (**2g**) was reacted with benzylamine (**3a**) which gave amide **4aa** in 91% yield. Likewise, phenyl acetic acid (**2e**) was reacted with an aromatic amine with a strongly electron-withdrawing nitro group, 4-nitroaniline (**3i**), yielding **4ab** in 60% yield.

Mechanistically, we propose the formation of a key intermediate triacyloxyborane-amine complex to occur through successive dehydrogenative addition of the carboxylic acid to pyridine borane, closely related to our earlier reports on borane-amine promoted carboxamide synthesis [18]. This intermediate then undergoes nucleophilic attack by the reactant amine to form the desired carboxamide.

**Scheme 4:**
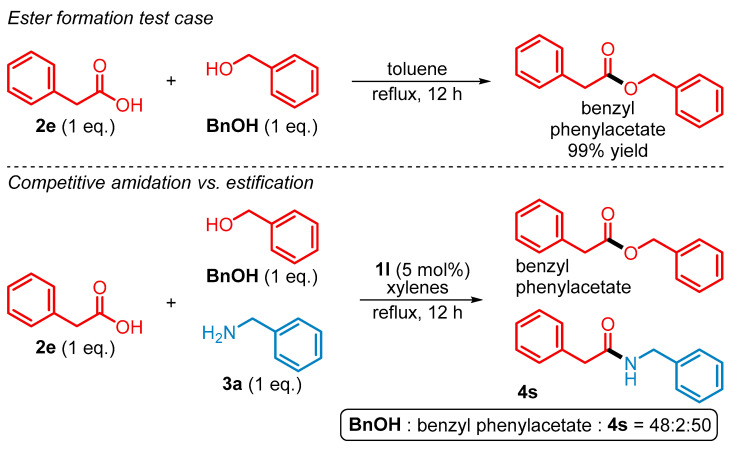
Competition between amide and ester formation.

To determine the selectivity of the carboxamide formation versus the reaction of the triacyloxyborane-amine complex with other potential nucleophiles, a reaction with a competing esterification was performed (Scheme 4). A baseline esterification between phenylacetic acid (**2e**) and benzyl alcohol (**BnOH**) provided the ester benzyl phenylacetate in nearly quantitative yield after 12 h in refluxing toluene, despite the lack of any added catalyst. Following this baseline esterification, a reaction with a competing amidation and esterification was performed. Equimolar quantities of phenylacetic acid (**2e**), benzyl alcohol (**BnOH**), and benzylamine (**3a**), along with 5 mol% of borane-pyridine (**1l**) were heated in refluxing xylenes for 12 h. The resulting reaction mixture was analyzed using ^1^H NMR spectroscopy where a ratio of 48:2:50 of the unreacted benzyl alcohol to the benzyl phenylacetate ester to the amide **4s** was observed, indicating a very high degree of selectivity towards the amidation versus the competing esterification. Further details can be found in the Supplementary Materials.

## 3. Materials and Methods

### 3.1. General Information

Unless otherwise noted, all additions were carried out under open air conditions. ^11^B, ^13^C, and ^1^H NMR spectra were recorded at room temperature, on a Bruker 300 MHz NMR spectrophotometer. Chemical shifts (δ values) are reported in parts per million relative to BF_3_.Et_2_O for ^11^B NMR, respectively. Data are reported as: δ value, multiplicity (s = singlet, d = doublet, t = triplet, q = quartet, m = multiplet, br = broad), and integration. All solvents for routine isolation of products were reagent-grade. Amines, carboxylic acids, and sodium borohydride were purchased from Sigma-Aldrich and/or Oakwood Chemicals and used without further purification. All borane-amines used for optimization studies were prepared using the reported procedure [20]. All reactions requiring heat were brought to temperature using an oil bath and heated stir plate.

### 3.2. Experimental

Procedure for the preparation of borane-pyridine [19] consisted of sodium borohydride (2 eq, 0.2 mole, 7.566 g) being charged to a 200 mL round-bottom flask containing a stir-bar followed by the addition of 100 mL ethyl acetate (1 M w.r.t. amine). With stirring, pyridine (1 eq, 0.1 mole, 8.05 mL) was added with the help of a syringe and, subsequently, 15 mL of water was added in three intervals (vigorous stirring required). After completion of the reaction at 20 h, the reaction mixture was transferred to a 500 mL separatory funnel using 100 mL ethyl acetate and 50 mL water. The organic layer was subjected to two additional washes with 50mL portions of water, then 50 mL of brine, and dried over sodium sulfate. The mixture was then filtered through cotton and condensed via rotary evaporation. The condensed liquid was passed through celite in a cotton-plugged pipette and washed with small ~5 mL portion of dichloromethane. The resulting colorless liquid was stirred under high vacuum to remove any remaining solvent and used without further purification. Characterization data for borane-pyridine can be found in the Supplementary Materials.

General procedure for the preparation of amides consisted of a 100 mL round bottom flask containing a magnetic stir-bar being charged with carboxylic acid (5.5 mmol, 1.1 eq.) and xylenes (5 mL, 1 M with respect to amine). To this stirring mixture, pyridine-borane ((0.25 mmol, 0.05 eq.) or (0.5 mmol, 0.1 eq.) or (2.5 mmol, 0.5 eq.)) was introduced followed by the addition of the respective amine (5 mmol, 1 eq.). A reflux condenser was attached to the flask and the reaction mixture was brought to reflux using an oil bath. After completion (12 h), the reaction mixture was diluted with methanol (50 mL) and condensed via rotary evaporation. The crude mixture was diluted with dichloromethane (15 mL) and transferred to a separatory funnel. The mixture was first washed with cold 3M sodium hydroxide solution (2 × 10 mL) and then the separated organic layer was further washed with 3M HCl (2 × 10 mL). The resultant organic layer was dried with sodium sulfate, filtered through cotton, and condensed via rotary evaporation followed by drying in vacuo for 12 h. The reaction also worked well at 100 mmol scale, w.r.t. the amine. Characterization data for the amide products can be found in the Supplementary Material, along with references of previous reports which provide support for the identities of the amides [17,18,21,22,23,24,25,26,27,28,29,30,31].

## 4. Conclusions

In conclusion, we have explored the reactivity of a new sub-stoichiometric reagent for the direct amidation of carboxylic acids and amines, pyridine-borane. Owing to its liquid nature and ease of preparation, it serves as an efficient alternative to the previously reported borane-ammonia. Borane-pyridine displayed a broad substrate scope with good quantitative yields of carboxamides containing both aromatic/aliphatic carboxylic acids and aryl/alkyl amines, as well as those containing potentially reactive halo, nitro, and alkene substituents. The decreased borane-pyridine loading of 5 mol% provides as additional advantage over previous borane-amine promoted methods of direct amidation.

## Data Availability

All of the ^1^H and ^13^C NMR spectra are available in the supporting information.

## Supplementary Materials

The following supporting information can be downloaded at: https://www.mdpi.com/article/10.3390/molecules29010268/s1, details of competitive reaction study, characterization data of borane-pyridine and product amides, NMR spectra of borane-pyridine and product amides.
