# Supplementary material for: Borane-Pyridine: An Efficient Catalyst for Direct Amidation
Source: Molecules. 2024 Jan 4;29(1):268. doi: 10.3390/molecules29010268 (PMC10780903; doi:10.3390/molecules29010268)
Supplement: Supplementary file 1 [file molecules-29-00268-s001.zip › molecules-2778164-supplementary.pdf]

SUPPORTING INFORMATION:

Borane-Pyridine: An Efficient Catalyst for Direct Amidation

P. Veeraraghavan Ramachandran*, Aman Singh, Harry Walker, and Henry J. Hamann

Herbert C. Brown Center for Borane Research, Department of Chemistry, Purdue University, West Lafayette, Indiana 47907, United States

E-mail: chandran@purdue.edu

Contents:

Page

Competitive studies.......... S2-S3

Characterization of borane-pyridine S4

Characterization of product amides S4-S7

References.......... S8

NMR spectra of amidation products.......... S9-S37

Competitive studies

Preparation of benzyl phenylacetate

A 25 ml round bottom flask was charged with phenylacetic acid (0.15g, 1.1 mmol, 1.1 equiv) and a magnetic stirring bar. Toluene was added (2 mL, 0.5 M solution with respect to alcohol) followed by the addition of benzyl alcohol (0.1 mL, 1 mmol) to the mixture. A condenser was affixed to the flask and the reaction mixture was then refluxed for 24 hours. After cooling to room temperature, the reaction mixture was transferred to a separatory funnel with DCM and washed with 1M HCL (1 x 10mL), sat. sodium bicarbonate (2 x 10 mL), and brine (1 x 10 mL). The organic layer was dried over anhydrous sodium sulfate, filtered, and concentrated under vacuum.

Characterization of benzyl phenylacetate

Benzyl phenylacetate

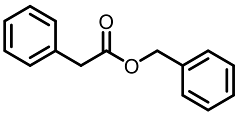

99% Yield (0.223 g), White Solid.

^{1}H NMR (300 MHz, $CDCl_3$): δ 7.61 – 7.28 (m, 10H), 5.23 (s, 2H), 3.76 (s, 2H). δ 8.60 (dd, *J* = 5.3, 3.0 Hz, 2H), 7.93 (t, *J* = 7.7 Hz, 1H), 7.57 – 7.46 (m, 2H), 3.19 – 1.98 (m, 3H).
^{13}C {^{1}H} NMR δ 170.6, 135.2, 133.3, 128.7, 127.9, 127.9, 127.6, 127.5, 126.5, 66.0, 40.8.

NMR Spectra of benzyl phenylacetate

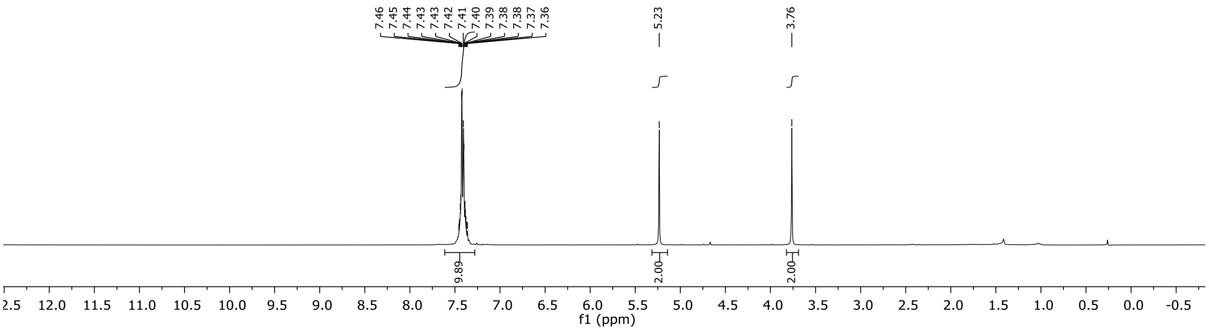

^{1}H NMR (300 MHz, Chloroform-*d*) Benzyl phenylacetate

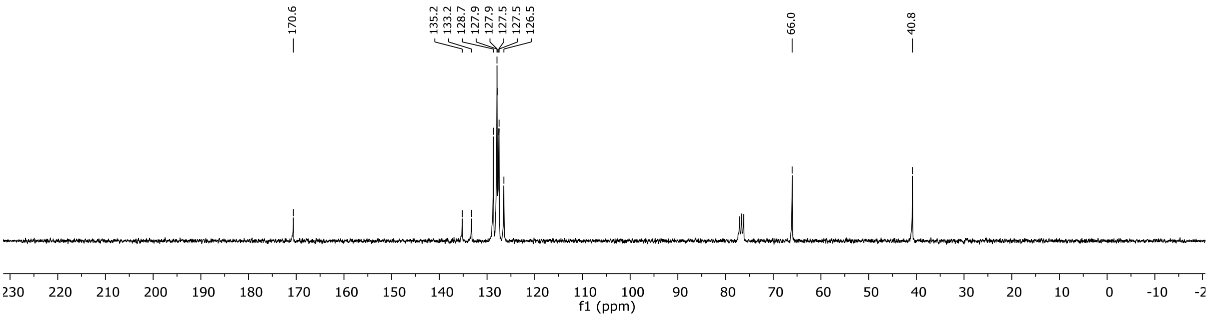

^{13}C NMR (75 MHz, Chloroform-*d*) Benzyl phenylacetate

Procedure for competitive amidation/esterification reaction

A 25 ml round bottom flask was charged with phenylacetic acid (0.749 g, 5.5 mmol, 1.1 equiv) and a magnetic stirring bar. Xylenes was added (5 mL, 1 M solution with respect to alcohol) followed by the addition of benzyl alcohol (0.52 mL, 5 mmol, 1 equiv), benzylamine (0.55 mL, 5 mmol, 1 equiv) , and borane-pyridine (0.023 g, 0.25 mmol, 0.05 equiv) to the mixture. A condenser was affixed to the flask and the reaction mixture was then refluxed for 24 hours. After cooling to room temperature, the solvent was evaporated, and reaction mixture was analyzed by ^{1}H NMR spectroscopy.

Competitive NMR

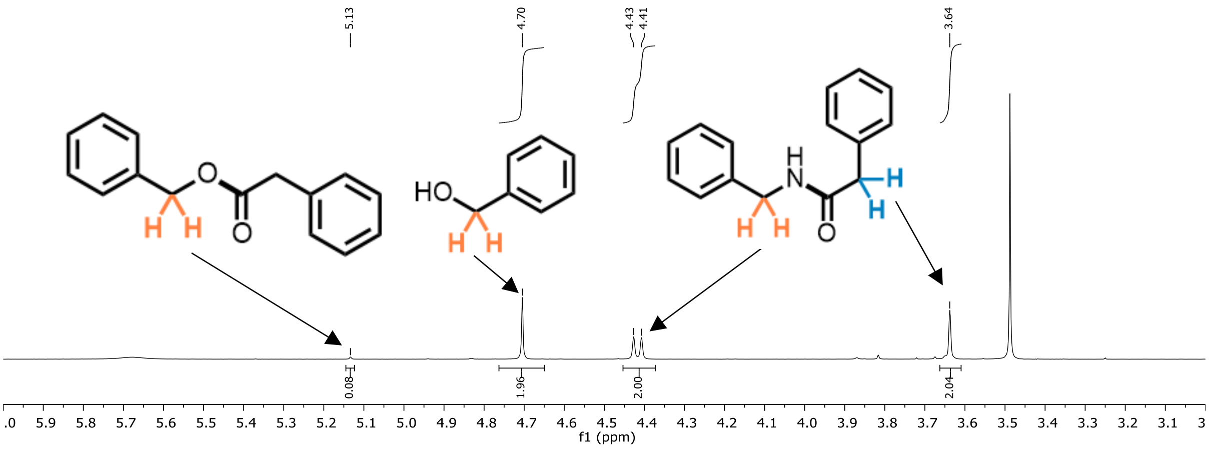

^{1}H NMR (300 MHz, Chloroform-*d*) Crude reaction mixture from competitive reaction.

Characterization of borane-pyridine

*Borane-pyridine (**1l**);* The compound was prepared as described in the procedure for the preparation of borane-pyridine; **^{1}H NMR** (300 MHz, $CDCl_3$): δ 8.60 (dd, J = 5.3, 3.0 Hz, 2H), 7.93 (t, J = 7.7 Hz, 1H), 7.57 – 7.46 (m, 2H), 3.19 – 1.98 (m, 3H). **^{13}C{H} NMR** (75 MHz, $CDCl_3$): δ 147.5, 139.1, 125.4. **^{11}B NMR** (96 MHz, $CDCl_3$): δ -12.25 (q, $^1J(^{11}B, ^1H)$ = 97.8 Hz). Characterization is in agreement with previous reports of this compound [1].

Characterization of product amides

*N-benzylbenzamide (**4a**);* The compound was prepared as described in the general procedure (5 mol%, white solid, mass = 1.046 g, 99% yield; 10 mol%, white solid, mass = 1.042 g, 99% yield); **^{1}H NMR** (300 MHz, $CDCl_3$): δ 7.84 – 7.75 (m, 2H), 7.57 – 7.28 (m, 8H), 6.41 (s, 1H), 4.66 (d, J = 5.6 Hz, 2H). **^{13}C {H} NMR** (75 MHz, $CDCl_3$) δ 167.4 (**C**=O), 138.2, 134.4, 131.6, 128.8, 128.6, 128.0, 127.7, 127.0, 44.2 (-**C**H_2-). Characterization is in agreement with previous reports of this compound [2].

*N-cyclohexylbenzamide (**4b**);* The compound was prepared as described in the general procedure (5 mol%, pink solid, mass = 0.696 g, 69% yield; 10 mol%, pink solid, mass = 0.653 g, 65% yield); **^{1}H NMR** (300 MHz, $CDCl_3$) δ 7.78 – 7.71 (m, 2H), 7.52 – 7.37 (m, 3H), 5.97 (s, 1H), 3.98 (dddd, J = 14.6, 10.5, 8.0, 3.9 Hz, 1H), 2.03 (dt, J = 12.2, 3.9 Hz, 2H), 1.82 – 1.60 (m, 3H), 1.52 – 1.35 (m, 2H), 1.32 – 1.15 (m, 3H). **^{13}C{H} NMR** (75 MHz, $CDCl_3$) δ 166.6 (**C**=O), 135.1, 131.3, 128.5, 126.8, 48.7 (-N**C**H-), 33.3, 25.6, 24.9. Characterization is in agreement with previous reports of this compound [2].

*N-hexylbenzamide (**4c**);* The compound was prepared as described in the general procedure (5 mol%, white solid, mass = 1.016 g, 99% yield; 10 mol%, pale yellow liquid, mass = 0.830 g, 81% yield); **^{1}H NMR** (300 MHz, $CDCl_3$): δ 7.80 – 7.71 (m, 2H), 7.51 – 7.43 (m, 1H), 7.43 – 7.34 (m, 2H), 6.39 (s, 1H), 3.49 – 3.34 (m, 2H), 1.59 (tt, J = 8.3, 6.7 Hz, 2H), 1.40 – 1.23 (m, 6H), 0.88 (t, J = 6.6 Hz, 3H). **^{13}C{H} NMR** (75 MHz, $CDCl_3$): δ 167.6 (**C**=O), 134.9, 131.3, 128.5, 126.9, 40.1 (-N**C**H_2-), 31.5, 29.6, 26.7, 22.6, 14.1 (-**C**H_3). Characterization is in agreement with previous reports of this compound [2].

*Morpholino(phenyl)methanone (**4d**);* The compound was prepared as described in the general procedure (10 mol%, brown solid, mass = 0.806 g, 84% yield); **^{1}H NMR** (300 MHz, $CDCl_3$): ^{1}H NMR (300 MHz, Chloroform-d) δ 7.36 (s, 5H), 3.84 – 3.55 (m, 6H), 3.42 (s, 2H). **^{13}C{H} NMR** (75 MHz, $CDCl_3$): δ 170.5 (**C**=O), 135.3, 129.9, 128.6, 127.1, 66.9 (-O**C**H_2-), 48.2 (-N**C**H_2-), 42.6 (-N**C**H_2-). Characterization is in agreement with previous reports of this compound [3].

*N,N-dibenzylbenzamide (**4e**);* The compound was prepared as described in the general procedure (10 mol%, light brown solid, mass = 1.055 g, 70% yield; 50 mol%, peach solid, mass = 1.185 g, 79% yield); **^{1}H NMR** (300 MHz, $CDCl_3$): δ 7.50 (tdd, J = 4.7, 3.5, 2.1 Hz, 2H), 7.42 – 7.27 (m, 11H), 7.15 (s, 2H), 4.70 (s, 2H), 4.40 (s, 2H). **^{13}C{H} NMR** (75 MHz, $CDCl_3$): δ 172.3 (**C**=O), 136.9, 136.2, 130.4, 129.7, 129.1, 128.8, 128.6, 128.4, 127.6, 127.1, 126.7, 51.6 (-N**C**H_2-), 46.9 (-N**C**H_2-). Characterization is in agreement with previous reports of this compound [2].

*N-benzyl-4-nitrobenzamide (**4f**);* The compound was prepared as described in the general procedure (5 mol%, yellow solid, mass = 1.031 g, 81% yield); **^{1}H NMR** (300 MHz, $CDCl_3$): δ 8.30 (d, J = 8.8 Hz, 2H), 7.95 (d, J = 8.8 Hz, 2H), 7.45 – 7.32 (m, 5H), 6.41 (s, 1H), 4.68 (d, J = 5.6 Hz, 2H). **^{13}C{H} NMR** (75 MHz, $CDCl_3$) δ 165.3 (**C**=O), 139.9, 137.4, 129.0, 128.2, 128.0, 123.9, 44.5 (-**C**H_2-). Characterization is in agreement with previous reports of this compound [2].

*N-benzylcinnamamide (**4g**);* The compound was prepared as described in the general procedure (5 mol%, yellow solid, mass = 1.174 g, 99% yield; 10 mol%, light yellow solid, mass = 1.022 g, 86% yield); **^{1}H NMR** (300 MHz, $CDCl_3$): δ 7.59 (d, J = 15.6 Hz, 1H), 7.46 – 7.36 (m, 2H), 7.31 – 7.15 (m, 8H), 6.36 (d, J = 15.6 Hz, 1H), 6.08 (s, 1H), 4.47 (d, J = 5.8 Hz, 2H). **^{13}C{H} NMR** (75 MHz, $CDCl_3$) δ 165.9 (**C**=O), 141.4, 138.2, 134.8, 129.7, 128.8, 128.7, 127.9, 127.8, 127.6, 120.4, 43.9 (-**C**H_2-). Characterization is in agreement with previous reports of this compound [2].

*N-cyclohexylcinnamamide (**4h**)*; The compound was prepared as described in the general procedure (5 mol%, white solid, mass = 1.135 g, 99% yield); **^{1}H NMR** (300 MHz, $CDCl_3$): δ 7.61 (d, J = 15.5 Hz, 1H), 7.54 – 7.43 (m, 2H), 7.42 – 7.29 (m, 3H), 6.36 (d, J = 15.5 Hz, 1H), 5.51 (d, J = 6.9 Hz, 1H), 3.92 (dddd, J = 12.1, 8.2, 7.3, 4.0 Hz, 1H), 2.09 – 1.91 (m, 2H), 1.79 – 1.60 (m, 3H), 1.50 – 1.31 (m, 2H), 1.29 – 1.08 (m, 3H). **^{13}C{H} NMR** (75 MHz, $CDCl_3$): δ 164.9 (**C**=O), 140.7, 135.0, 129.5, 128.8, 127.7, 121.2, 48.4 (-N**C**H-), 33.3, 25.6, 24.9. Characterization is in agreement with previous reports of this compound [4].

*N-hexylcinnamamide (**4i**)*; The compound was prepared as described in the general procedure (5 mol%, white solid, mass = 1.074 g, 93% yield; 10 mol%, white solid, mass = 1.145 g, 99% yield); **^{1}H NMR** (300 MHz, $CDCl_3$): δ 7.62 (d, J = 15.6 Hz, 1H), 7.47 (d, J = 2.0 Hz, 2H), 7.32 (d, J = 2.4 Hz, 3H), 6.43 (d, J = 15.6 Hz, 1H), 5.93 (s, 1H), 3.37 (td, J = 7.2, 5.8 Hz, 2H), 1.64 – 1.48 (m, 2H), 1.30 (dd, J = 4.2, 2.1 Hz, 6H), 0.87 (t, J = 6.6 Hz, 3H). **^{13}C{H} NMR** (75 MHz, $CDCl_3$): δ 166.3 (**C**=O), 140.4, 135.0, 129.5, 128.8, 127.7, 121.3, 39.9 (-N**C**H-), 31.6, 29.7, 26.8, 22.6, 14.0 (-**C**H_3). Characterization is in agreement with previous reports of this compound [5].

*(E)-1-morpholino-3-phenylprop-2-en-1-one (**4j**)*; The compound was prepared as described in the general procedure (5 mol%, off-white solid, mass = 1.075 g, 99% yield); **^{1}H NMR** (300 MHz, $CDCl_3$): δ 7.70 (d, J = 15.4 Hz, 1H), 7.56 – 7.49 (m, 2H), 7.37 (dd, J = 5.1, 2.1 Hz, 3H), 6.84 (d, J = 15.4 Hz, 1H), 3.78 – 3.63 (m, 8H). **^{13}C{H} NMR** (75 MHz, $CDCl_3$): δ 165.5 (**C**=O), 143.2, 135.1, 129.8, 128.8, 127.8, 116.6, 66.8 (-O**C**H_2-), 46.2 (-N**C**H_2-), 42.5 (-N**C**H_2-). Characterization is in agreement with previous reports of this compound [6].

*N-phenylbenzamide (**4k**)*; The compound was prepared as described in the general procedure (5 mol%, white solid, mass = 0.664 g, 68% yield; 10 mol%, light yellow solid, mass =0.680 g, 69% yield); **^{1}H NMR** (300 MHz, $CDCl_3$) δ 7.91 – 7.85 (m, 2H), 7.82 (s, 1H), 7.69 – 7.62 (m, 2H), 7.58 – 7.46 (m, 3H), 7.42 – 7.34 (m, 2H), 7.20 – 7.12 (m, 1H). **^{13}C{H} NMR** (75 MHz, $CDCl_3$) δ 165.8 (**C**=O), 137.9, 135.0, 131.9, 129.1, 128.8, 127.0, 124.6, 120.2. Characterization is in agreement with previous reports of this compound [2].

*N-(4-methoxyphenyl)benzamide (**4l**)*; The compound was prepared as described in the general procedure (5 mol%, gray solid, mass = 0.825 g, 73% yield; 10 mol%, green solid, mass = 0.869 g, 76% yield); **^{1}H NMR** (300 MHz, $CDCl_3$): δ 7.94 – 7.84 (m, 2H), 7.73 (s, 1H), 7.60 – 7.43 (m, 5H), 6.97 – 6.86 (m, 2H), 3.85 (s, 3H). **^{13}C{H} NMR** (75 MHz, $CDCl_3$): δ 165.7 (**C**=O), 156.6, 135.0, 131.7, 131.0, 128.8, 127.0, 122.1, 114.2, 55.5 (-O**C**H_3). Characterization is in agreement with previous reports of this compound [7].

*2-methyl-N-phenylbenzamide (**4m**)*; The compound was prepared as described in the general procedure (5 mol%, brown solid, mass = 0.439 g, 41% yield; 10 mol%, brown solid, mass = 0.570 g, 54% yield); **^{1}H NMR** (300 MHz, $CDCl_3$): ^{1}H NMR (300 MHz, Chloroform-d) δ 7.62 (d, J = 8.0 Hz, 2H), 7.49 (d, J = 7.7 Hz, 2H), 7.37 (td, J = 7.5, 1.7 Hz, 3H), 7.27 (d, J = 8.1 Hz, 2H), 7.16 (tt, J = 7.0, 1.2 Hz, 1H), 2.51 (s, 3H). **^{13}C{H} NMR** (75 MHz, $CDCl_3$): δ 168.1 (**C**=O), 138.0, 136.5, 131.3, 130.3, 129.1, 126.6, 125.9, 124.6, 119.9, 19.8 (-**C**H_3). Characterization is in agreement with previous reports of this compound [8].

*N-(4-methoxyphenyl)-2-methylbenzamide (**4n**)*; The compound was prepared as described in the general procedure (5 mol%, off-white solid, mass = 1.050 g, 87% yield; 10 mol%, off-white solid, mass = 0.960 g, 80% yield); **^{1}H NMR** (300 MHz, $CDCl_3$): δ 7.53 (d, J = 9.0 Hz, 2H), 7.49 (d, J = 7.6 Hz, 1H), 7.41 – 7.30 (m, 2H), 7.28 (s, 1H), 6.91 (d, J = 9.0 Hz, 2H), 3.82 (s, 3H), 2.52 (s, 3H). **^{13}C{H} NMR** (75 MHz, $CDCl_3$): δ 168.0 (**C**=O), 156.6, 136.5, 136.4, 131.2, 130.2, 126.6, 125.9, 121.8, 114.3, 55.5 (-O**C**H_3), 19.8(-**C**H_3). Characterization is in agreement with previous reports of this compound [9].

*N-phenylcinnamamide (**4o**)*; The compound was prepared as described in the general procedure (5 mol%, green solid, mass = 1.062 g, 95% yield; 10 mol%, green solid, mass = 1.008 g, 90% yield); **^{1}H NMR** (300 MHz, $CDCl_3$): δ 7.75 (d, J = 15.5 Hz, 1H), 7.71 (s, 1H), 7.64 (d, J = 7.9 Hz, 2H), 7.56 – 7.44 (m, 2H), 7.42 – 7.30 (m, 5H), 7.14 (d, J = 7.3 Hz, 1H), 6.60 (d, J = 15.5 Hz, 1H). **^{13}C{H} NMR** (75 MHz, $CDCl_3$) δ 164.4 (**C**=O), 142.4, 138.1, 134.6, 130.0, 129.1, 128.9, 128.0, 124.5, 121.0, 120.2. Characterization is in agreement with previous reports of this compound [8].

N,2-diphenylacetamide (**4p**); The compound was prepared as described in the general procedure (5 mol%, yellow solid, mass = 1.048 g, 99% yield; (1.048g), 10 mol%, green solid, mass = 1.046 g, 99% yield); ^{1}**H NMR** (300 MHz, $CDCl_3$): δ 7.45 – 7.37 (m, 4H), 7.36 – 7.24 (m, 5H), 7.12 – 7.06 (m, 1H), 7.05 (s, 1H), 3.75 (s, 2H). ^{13}**C{H} NMR** (75 MHz, $CDCl_3$): δ 169.2 (**C**=O), 137.7, 134.5, 129.6, 129.2, 129.0, 127.7, 124.5, 119.9, 44.8 (-**C**H_2-). Characterization is in agreement with previous reports of this compound [8].

N-(4-methoxyphenyl)-2-phenylacetamide (**4q**); The compound was prepared as described in the general procedure (5 mol%, brown solid, mass = 1.148 g, 95% yield; 10 mol%, brown solid, mass = 0.926 g, 77% yield); ^{1}**H NMR** (300 MHz, $CDCl_3$): δ 7.47 – 7.36 (m, 2H), 7.37 – 7.27 (m, 5H), 6.92 (s, 1H), 6.85 – 6.77 (m, 2H), 3.77 (s, 3H), 3.73 (s, 2H). ^{13}**C{H} NMR** (75 MHz, $CDCl_3$): δ 169.1 (**C**=O), 156.5, 134.6, 130.7, 129.6, 129.2, 127.6, 121.9, 114.1, 55.5 (-O**C**H_3), 44.6 (-**C**H_2-). Characterization is in agreement with previous reports of this compound [10].

N-(3-bromophenyl)-2-phenylacetamide (**4r**); The compound was prepared as described in the general procedure (5 mol%, green solid, mass = 1.450 g, 99% yield; 10 mol%, green solid, mass = 1.436 g, 99% yield); ^{1}**H NMR (**300 MHz, $CDCl_3$): δ 7.65 (t, J = 2.0 Hz, 1H), 7.47 – 7.29 (m, 6H), 7.24 – 7.09 (m, 2H), 7.06 (s, 1H), 3.74 (s, 2H). ^{13}**C{H} NMR** (75 MHz, $CDCl_3$): δ 169.5 (**C**=O), 138.9, 134.1, 130.2, 129.5, 129.3, 127.8, 127.4, 122.8, 122.5, 118.4, 44.7 (-**C**H_2-). Characterization is in agreement with previous reports of this compound [11].

N-phenylcyclohexanecarboxamide (**4s**); The compound was prepared as described in the general procedure (5 mol%, green-yellow solid, mass = 0.761 g, 75% yield; 10 mol%, white solid, mass = 0.790 g, 78% yield); ^{1}**H NMR** (300 MHz, $CDCl_3$): δ 7.58 – 7.49 (m, 2H), 7.37 – 7.24 (m, 2H), 7.21 (s, 1H), 7.14 – 7.03 (m, 1H), 2.35 – 2.15 (m, 1H), 2.03 – 1.78 (m, 4H), 1.75 – 1.46 (m, 3H), 1.41 – 1.15 (m, 3H). ^{13}**C{H} NMR** (75 MHz, $CDCl_3$): δ 173.5 (**C**=O), 137.1, 127.9, 123.0, 118.7, 45.5 (-**C**H-), 28.6, 24.6. Characterization is in agreement with previous reports of this compound [8].

N-benzyl-2-phenylacetamide (**4t**); The compound was prepared as described in the general procedure (5 mol%, white solid, mass = 1.125 g, 99% yield; 10 mol%, white solid, mass = 1.120 g, 99% yield); ^{1}**H NMR** (300 MHz, $CDCl_3$) δ 7.39 – 7.24 (m, 8H), 7.21 – 7.15 (m, 2H), 5.68 (s, 1H), 4.42 (d, J = 5.8 Hz, 2H), 3.64 (s, 2H). ^{13}**C{H} NMR** (75 MHz, $CDCl_3$) δ 170.9 (**C**=O), 138.1, 134.8, 129.5, 129.1, 128.7, 127.5, 127.4, 43.9 (-N**C**H_2-), 43.6 (-**C**H_2-). Characterization is in agreement with previous reports of this compound [2].

N-cyclohexyl-2-phenylacetamide (**4u**); The compound was prepared as described in the general procedure (5 mol%, off-white solid, mass = 1.083 g, 99% yield; 10 mol%, off-white solid, mass= 1.076 g, 99% yield); ^{1}**H NMR** (300 MHz, $CDCl_3$): δ 7.41 – 7.20 (m, 5H), 5.19 (s, 1H), 3.75 (tdt, J = 10.5, 8.0, 4.0 Hz, 1H), 3.55 (s, 2H), 1.89 – 1.77 (m, 2H), 1.58 (ddd, J = 13.6, 10.3, 3.7 Hz, 3H), 1.41 – 1.23 (m, 2H), 1.18 – 0.90 (m, 3H). ^{13}**C{H} NMR** (75 MHz, $CDCl_3$) δ 170.0 (**C**=O), 135.2, 129.4, 129.0, 127.3, 48.2 (-N**C**H-), 44.0 (-**C**H_2-), 32.9, 25.5, 24.7. Characterization is in agreement with previous reports of this compound [10].

N-hexyl-2-phenylacetamide (**4v**); The compound was prepared as described in the general procedure (5 mol%, white solid, mass = 1.090 g, 99% yield; 10 mol%, white solid, mass = 1.012 g, 92% yield); ^{1}**H NMR** (300 MHz, $CDCl_3$): δ 7.42 – 7.18 (m, 5H), 5.36 (s, 1H), 3.56 (s, 2H), 3.19 (td, J = 7.2, 5.8 Hz, 2H), 1.46 – 1.34 (m, 2H), 1.28 – 1.15 (m, 6H), 0.84 (t, J = 6.8 Hz, 3H). ^{13}**C{H} NMR** (75 MHz, $CDCl_3$) δ 171.0 (**C**=O), 135.2, 129.4, 128.9, 127.2, 43.8 (-**C**H_2-), 39.7 (-N**C**H-), 31.4, 29.4, 26.5, 22.5, 14.0 (-**C**H_3). Characterization is in agreement with previous reports of this compound [12].

1-morpholino-2-phenylethan-1-one (**4w**); The compound was prepared as described in the general procedure (5 mol%, white solid, mass = 1.016 g, 99% yield; 10 mol%, white solid, mass = 0.982 g, 96% yield); ^{1}**H NMR** (300 MHz, $CDCl_3$): δ 7.38 – 7.30 (m, 2H), 7.30 – 7.21 (m, 3H), 3.74 (s, 2H), 3.65 (s, 4H), 3.51 – 3.41 (m, 4H). ^{13}**C{H} NMR** (75 MHz, $CDCl_3$): δ 169.6 (**C**=O), 134.8, 128.8, 128.6, 126.9, 66.8 (-O**C**H_2-), 66.4 (-O**C**H_2-), 46.5 (-N**C**H_2-), 42.1 (-N**C**H_2-), 40.8 (-**C**H_2-). Characterization is in agreement with previous reports of this compound [6].

*N-benzylcyclohexanecarboxamide (**4x**)*; The compound was prepared as described in the general procedure (5 mol%, brown solid, mass = 1.077 g, 99% yield; 10 mol%, white solid, mass = 1.082 g, 99% yield); ^{1}**H NMR** (300 MHz, $CDCl_3$): δ 7.41 – 7.22 (m, 5H), 5.70 (s, 1H), 4.44 (d, J = 5.6 Hz, 2H), 2.11 (tt, J = 11.7, 3.5 Hz, 1H), 1.95 – 1.74 (m, 4H), 1.72 – 1.57 (m, 1H), 1.47 (qd, J = 12.1, 3.1 Hz, 2H), 1.36 – 1.16 (m, 3H). ^{13}**C{H} NMR** (75 MHz, $CDCl_3$) δ 175.0 (**C**=O), 137.6, 127.6, 126.6, 126.4, 44.5 (-N**C**H_2-), 42.3 (-**C**H-), 28.7, 24.7. Characterization is in agreement with previous reports of this compound [2].

*N-cyclohexylcyclohexanecarboxamide (**4y**)*; The compound was prepared as described in the general procedure (5 mol%, off-white solid, mass = 0.919 g, 88% yield; 10 mol%, off-white solid, mass= 0.951 g, 91% yield); ^{1}**H NMR** (300 MHz, $CDCl_3$): δ 5.26 (s, 1H), 3.75 (tdt, J = 10.6, 8.0, 3.9 Hz, 1H), 2.01 (tt, J = 11.7, 3.4 Hz, 1H), 1.93 – 1.55 (m, 10H), 1.49 – 1.00 (m, 10H). ^{13}**C{H} NMR** (75 MHz, $CDCl_3$) δ 174.1 (**C**=O), 46.7 (-N**C**H-), 44.7 (-**C**H-), 32.2, 28.7, 24.7, 24.5, 23.8. Characterization is in agreement with previous reports of this compound [13].

*N-hexylcyclohexanecarboxamide (**4z**)*; The compound was prepared as described in the general procedure (5 mol%, brown solid, mass = 0.787 g, 74% yield; 10 mol%, off-white solid, mass = 1.036 g, 98% yield); ^{1}**H NMR** (300 MHz, $CDCl_3$): δ 5.40 (s, 1H), 3.32 – 3.14 (m, 2H), 2.04 (tt, J = 11.7, 3.4 Hz, 1H), 1.91 – 1.73 (m, 4H), 1.69 – 1.63 (m, 1H), 1.53 – 1.18 (m, 13H), 0.88 (t, J = 6.7 Hz, 3H). ^{13}**C{H} NMR** (75 MHz, $CDCl_3$) δ 175.0 (**C**=O), 44.6 (-N**C**H_2-), 38.3 (-**C**H-), 30.5, 28.7, 28.6, 25.5, 24.7, 21.5, 13.0 (-**C**H_3). Characterization is in agreement with previous reports of this compound [5].

*N-benzyl-4-methoxybenzamide (**4aa**)*; The compound was prepared as described in the general procedure (5 mol%, white solid, mass = 1.098 g, 91% yield); ^{1}**H NMR** (300 MHz, $CDCl_3$): δ 7.76 (d, J = 8.9 Hz, 2H), 7.32 (d, J = 4.4 Hz, 4H), 7.30 – 7.24 (m, 1H), 6.95 – 6.81 (m, 2H), 6.60 (t, J = 5.9 Hz, 1H), 4.59 (d, J = 5.7 Hz, 2H), 3.82 (s, 3H). ^{13}**C{H} NMR** (101 MHz, $CDCl_3$) δ 166.8 (**C**=O), 162.1, 138.4, 128.7, 128.6, 127.7, 127.4, 126.6, 113.6, 55.3 (-O**C**H_3), 43.9 (-**C**H_2-). Characterization is in agreement with previous reports of this compound [2].

*N-(4-nitrophenyl)-2-phenylacetamide (**4ab**)*; The compound was prepared as described in the general procedure (5 mol%, off-white solid, mass = 0.764 g, 60% yield); ^{1}**H NMR** (300 MHz, $CDCl_3$): ^{1}H NMR (400 MHz, Chloroform-d) δ 8.15 (d, J = 9.1 Hz, 2H), 7.60 (d, J = 9.2 Hz, 2H), 7.46 (s, 1H), 7.44 – 7.38 (m, 2H), 7.38 – 7.29 (m, 3H), 3.78 (s, 2H). ^{13}**C{H} NMR** (101 MHz, $CDCl_3$) ^{13}C NMR (101 MHz, Chloroform-d) δ 169.3 (**C**=O), 143.5, 143.3, 133.5, 129.4, 128.0, 124.9, 119.0, 44.8 (-**C**H_2-). Characterization is in agreement with previous reports of this compound [14].

References

1. Ramachandran, P. V.; Hamann, H. J.; Lin, R.; Singh, A., Scalable, Green Synthesis of Heteroaromatic Amine-boranes. *Org. Process Res. Dev.* **2023**, *27*, 775-783. 10.1021/acs.oprd.3c00031

2. Ramachandran, P. V.; Hamann, H. J., Ammonia-borane as a Catalyst for the Direct Amidation of Carboxylic Acids. *Org. Lett.* **2021**, *23*, 2938-2942. 10.1021/acs.orglett.1c00591

3. Ramachandran, P. V.; Hamann, H. J.; Choudhary, S., Amine-boranes as Dual-Purpose Reagents for Direct Amidation of Carboxylic Acids. *Org. Lett.* **2020**, *22*, 8593-8597. 10.1021/acs.orglett.0c03184

4. Duangkamol, C.; Jaita, S.; Wangngae, S.; Phakhodee, W.; Pattarawarapan, M., An efficient mechanochemical synthesis of amides and dipeptides using 2,4,6-trichloro-1,3,5-triazine and PPh_3. *RSC Adv.* **2015**, *5*, 52624-52628. 10.1039/C5RA10127A

5. Ohshima, T.; Hayashi, Y.; Agura, K.; Fujii, Y.; Yoshiyama, A.; Mashima, K., Sodium methoxide: a simple but highly efficient catalyst for the direct amidation of esters. *Chem. Comm.* **2012**, *48*, 5434-5436. 10.1039/C2CC32153J

6. Gockel, S. N.; Hull, K. L., Chloroform as a Carbon Monoxide Precursor: In or Ex Situ Generation of CO for Pd-Catalyzed Aminocarbonylations. *Org. Lett.* **2015**, *17*, 3236-3239. 10.1021/acs.orglett.5b01385

7. Manasa, K. L.; Tangella, Y.; Krishna, N. H.; Alvala, M., A metal-free approach for the synthesis of amides/esters with pyridinium salts of phenacyl bromides via oxidative C–C bond cleavage. *Beilstein J. Org. Chem.* **2019**, *15*, 1864-1871. 10.3762/bjoc.15.182

8. Ling, L.; Chen, C.; Luo, M.; Zeng, X., Chromium-Catalyzed Activation of Acyl C–O Bonds with Magnesium for Amidation of Esters with Nitroarenes. *Org. Lett.* **2019**, *21*, 1912-1916. 10.1021/acs.orglett.9b00554

9. Nozawa-Kumada, K.; Kadokawa, J.; Kameyama, T.; Kondo, Y., Copper-Catalyzed sp^3 C–H Aminative Cyclization of 2-Alkyl-N-arylbenzamides: An Approach for the Synthesis of N-Aryl-isoindolinones. *Org. Lett.* 2015, *17*, 4479-4481. 10.1021/acs.orglett.5b02235

10. Yu, W.; Yang, S.; Xiong, F.; Fan, T.; Feng, Y.; Huang, Y.; Fu, J.; Wang, T., Palladium-catalyzed carbonylation of benzylic ammonium salts to amides and esters via C–N bond activation. *Org. Biomol. Chem.* **2018**, *16*, 3099-3103. 10.1039/C8OB00488A

11. Sirgamalla, R.; Kommakula, A.; Banoth, S.; Dharavath, R.; Adem, K.; P, M.; Boda, S., Synthesis of Amides from Aliphatic Acids and Amines by using of I_2/TBHP at Room Temperature. *ChemistrySelect* **2018**, *3*, 1062-1065. 10.1002/slct.201702286

12. Gabriel, C. M.; Keener, M.; Gallou, F.; Lipshutz, B. H., Amide and Peptide Bond Formation in Water at Room Temperature. *Org. Lett.* **2015**, *17*, 3968-3971. 10.1021/acs.orglett.5b01812

13. Xiao, K.-J.; Wang, A.-E.; Huang, Y.-H.; Huang, P.-Q., Versatile and Direct Transformation of Secondary Amides into Ketones by Deaminative Alkylation with Organocerium Reagents. *Asian J. Org. Chem.* **2012**, *1*, 130-132. 10.1002/ajoc.201200066

14. Shao, J.; Huang, X. M.; Wang, S. Y.; Liu, B. X.; Xu, B., A straightforward synthesis of *N*-monosubstituted α-keto amides via aerobic benzylic oxidation of amides. *Tetrahedron* **2012**, *68*, 573-579. 10.1016/j.tet.2011.11.005

NMR Spectra of Borane-Pyridine and Amidation Products:

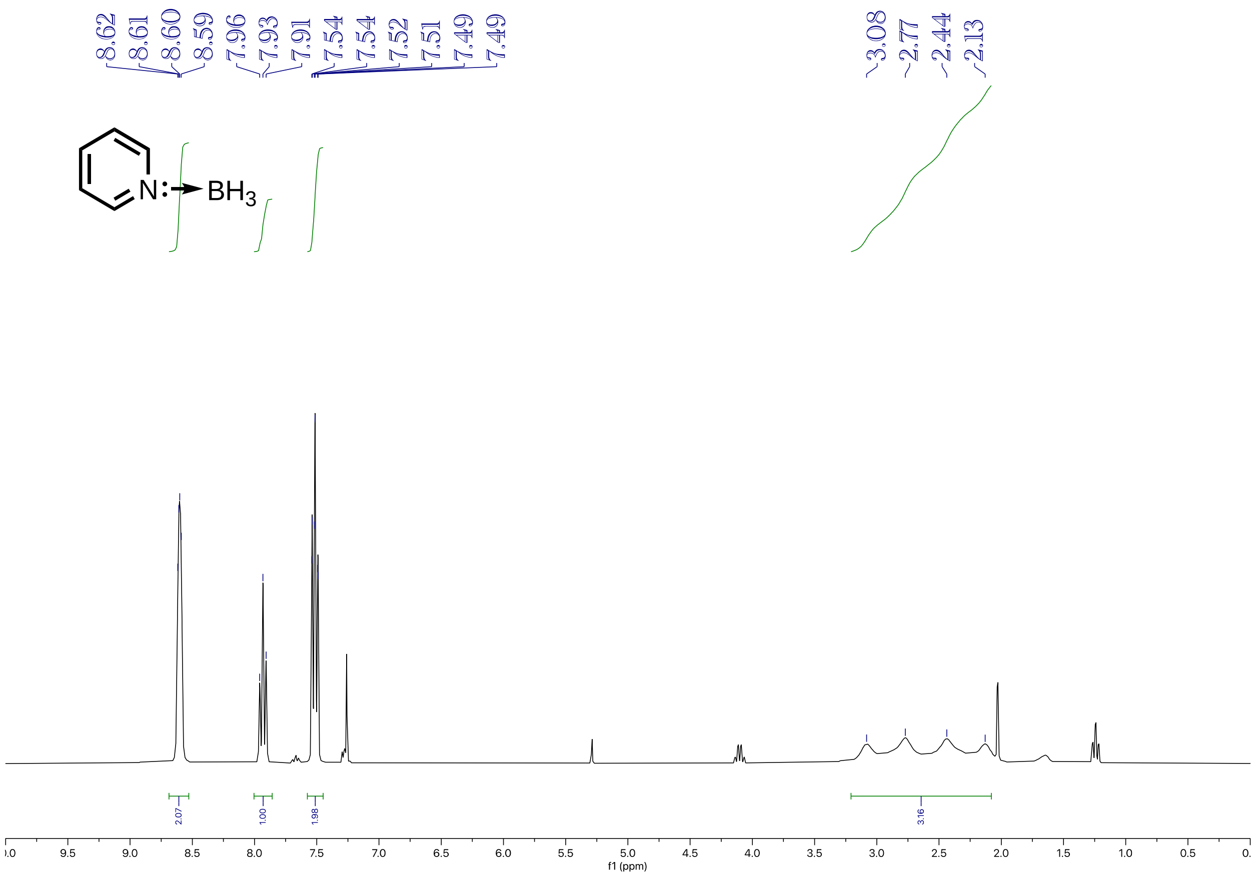

^{1}H NMR (300 MHz, Chloroform-*d*) Borane-pyridine (**1i**)

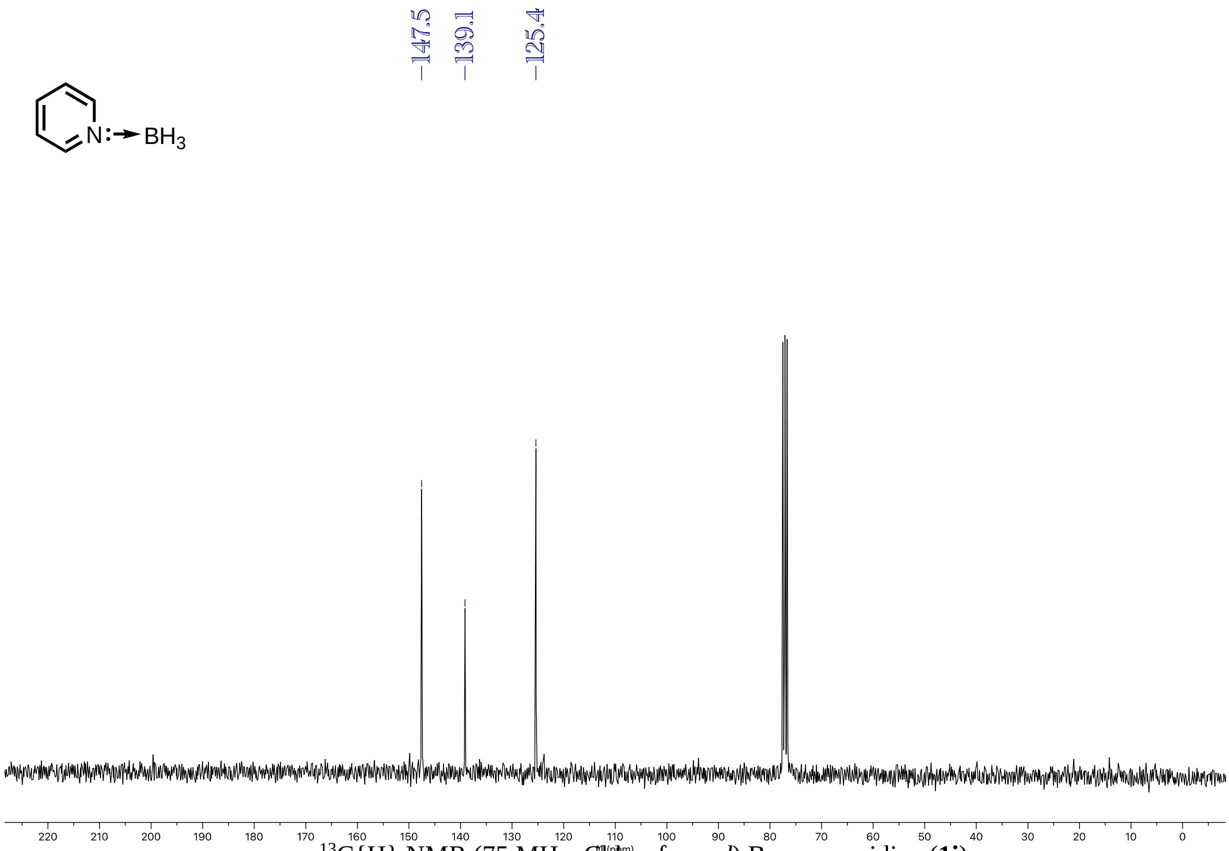

^{13}C{H} NMR (75 MHz, Chloroform-*d*) Borane-pyridine (**1i**)

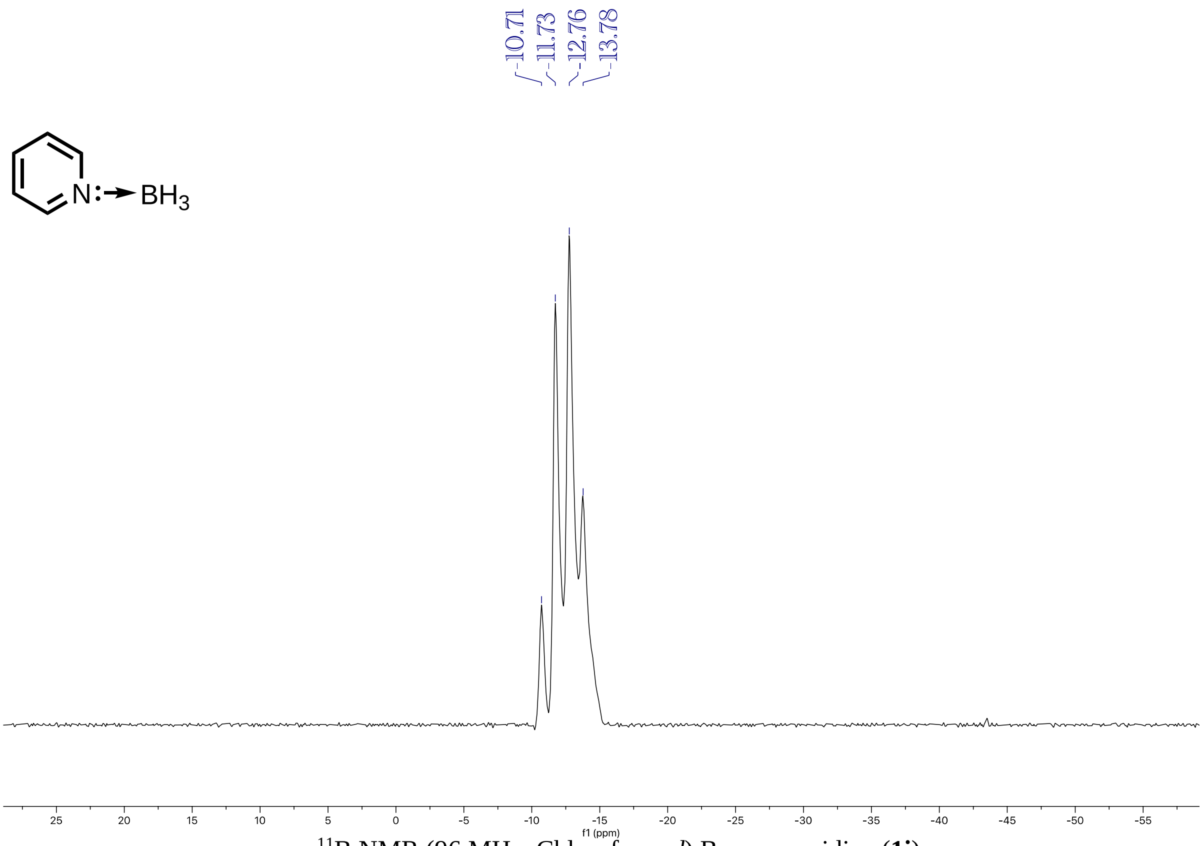

^{11}B NMR (96 MHz, Chloroform-*d*) Borane-pyridine (**1i**)

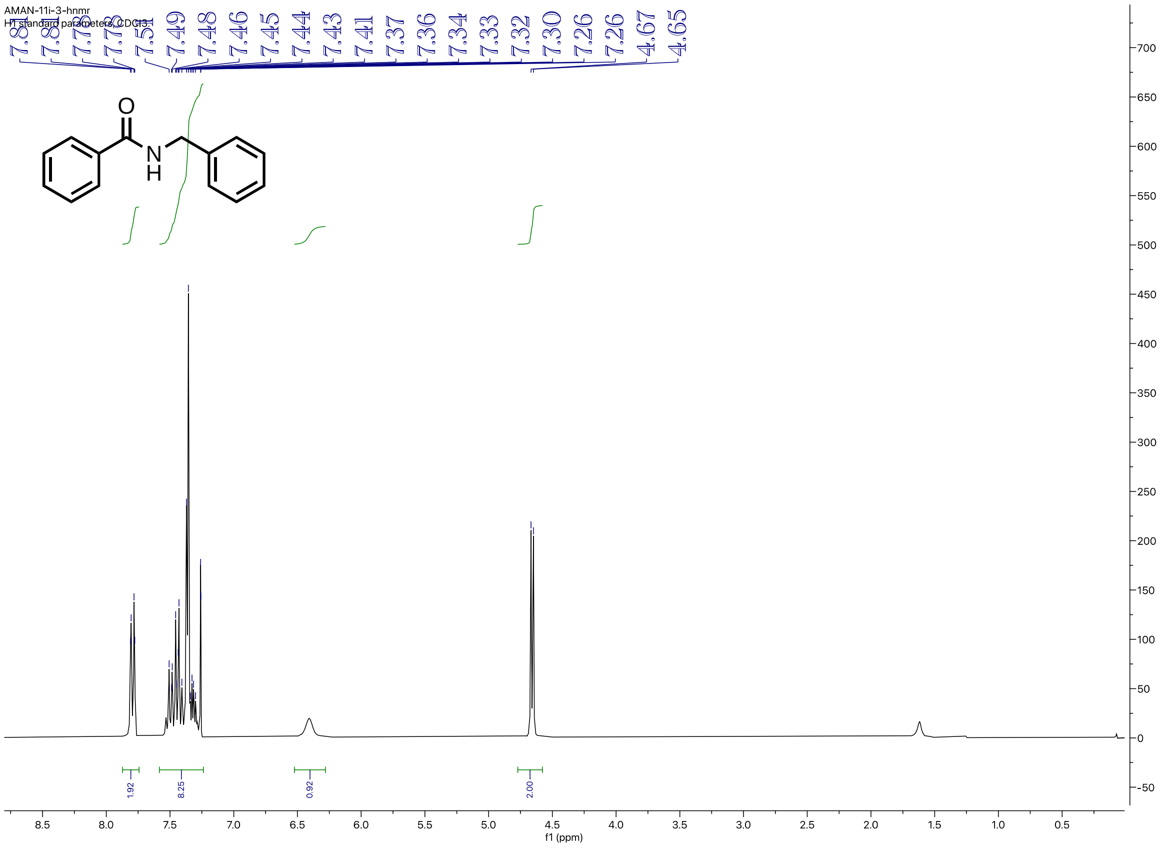

^{1}H NMR (300 MHz, Chloroform-*d*) *N*-benzylbenzamide (**4a**)

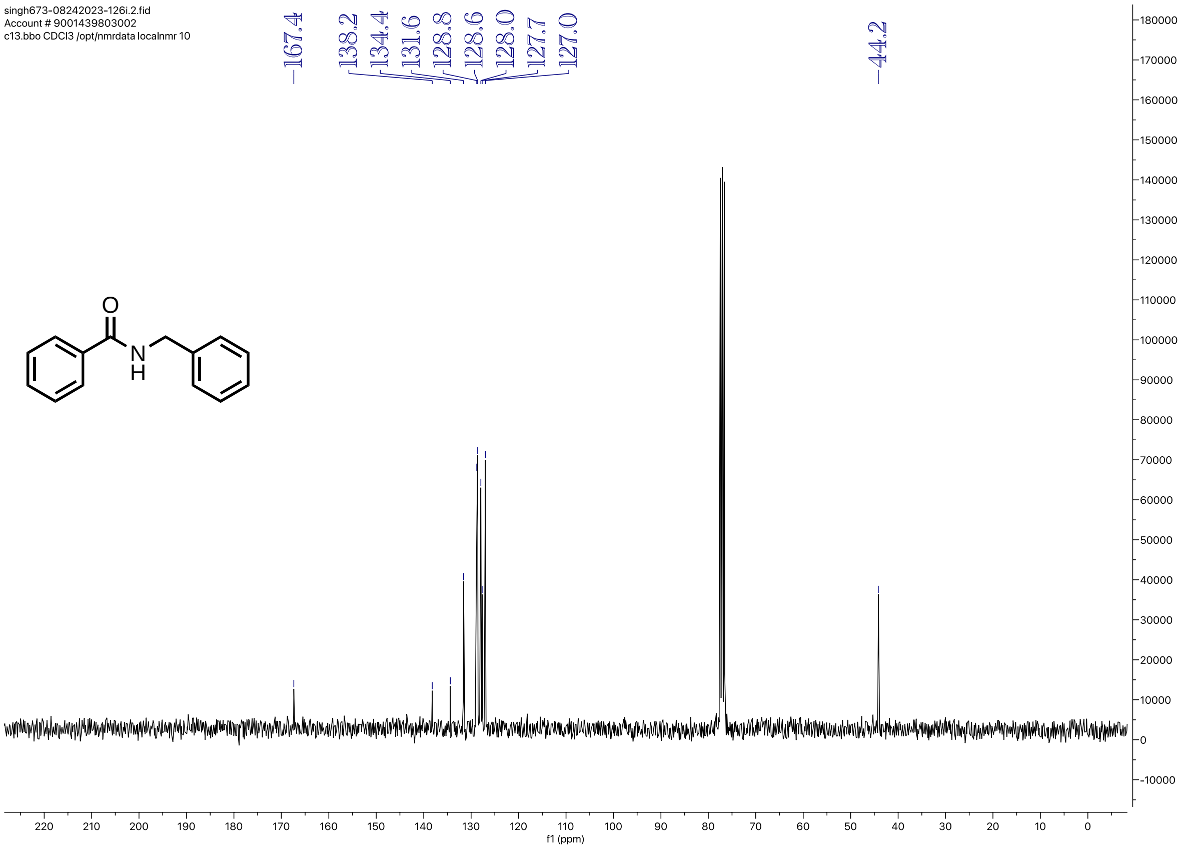

^{13}C{H} NMR (75 MHz, Chloroform-*d*) *N*-benzylbenzamide (**4a**)

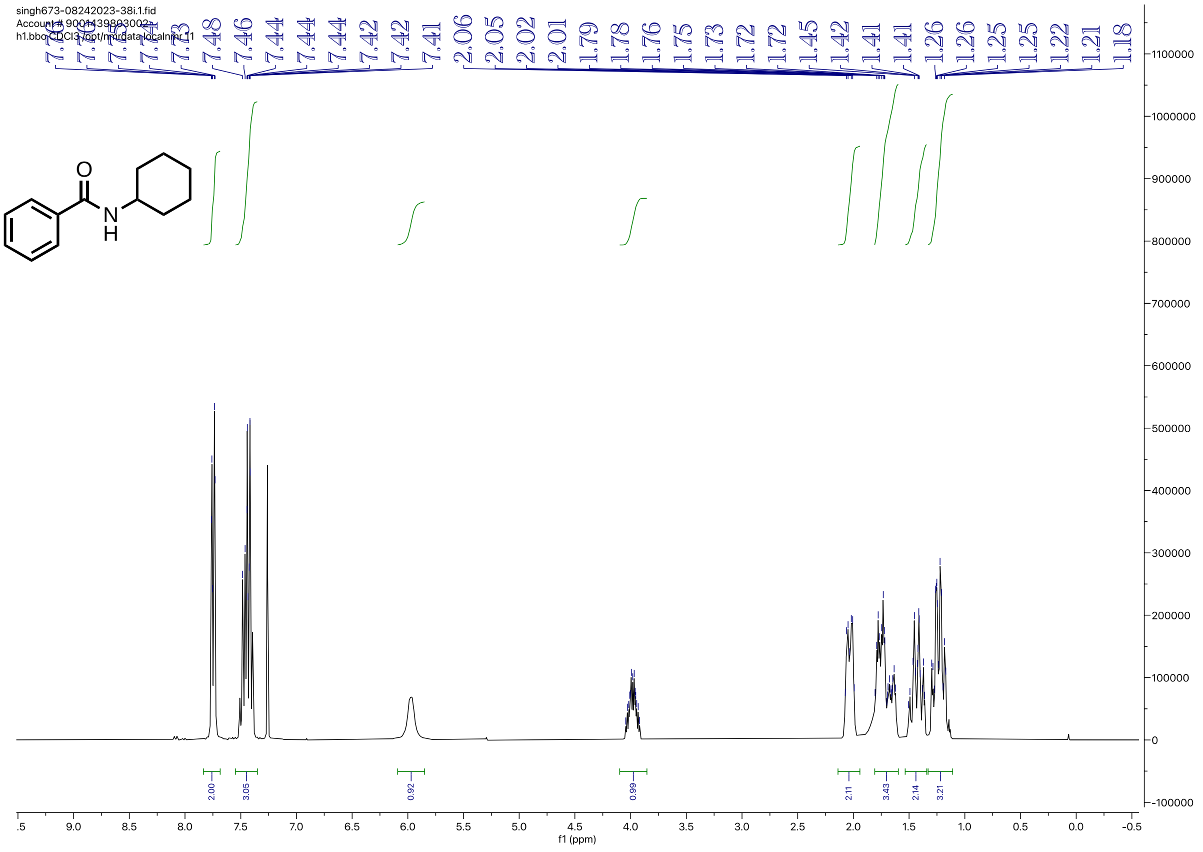

^{1}H NMR (300 MHz, Chloroform-*d*) *N*-cyclohexylbenzamide (**4b**)

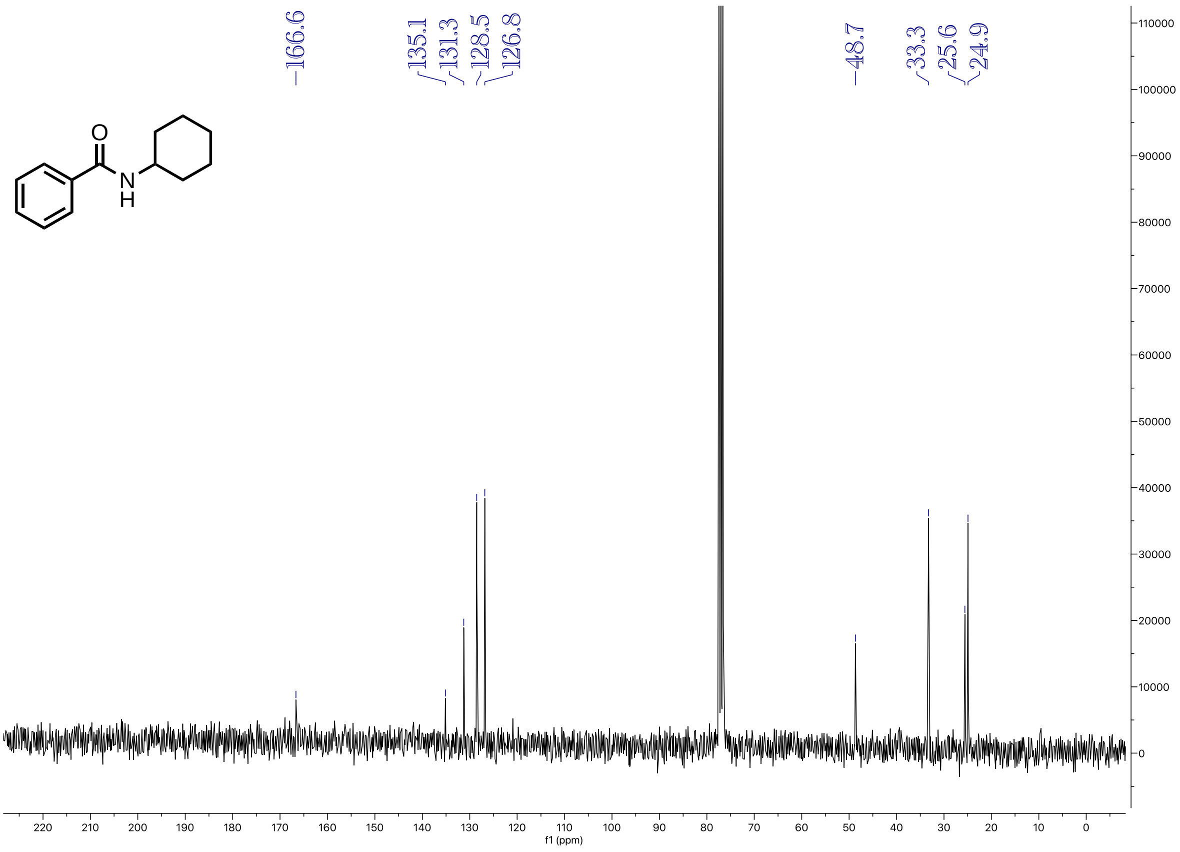

$^{13}C\{H\}$ NMR (75 MHz, Chloroform-*d*) *N*-cyclohexylbenzamide (**4b**)

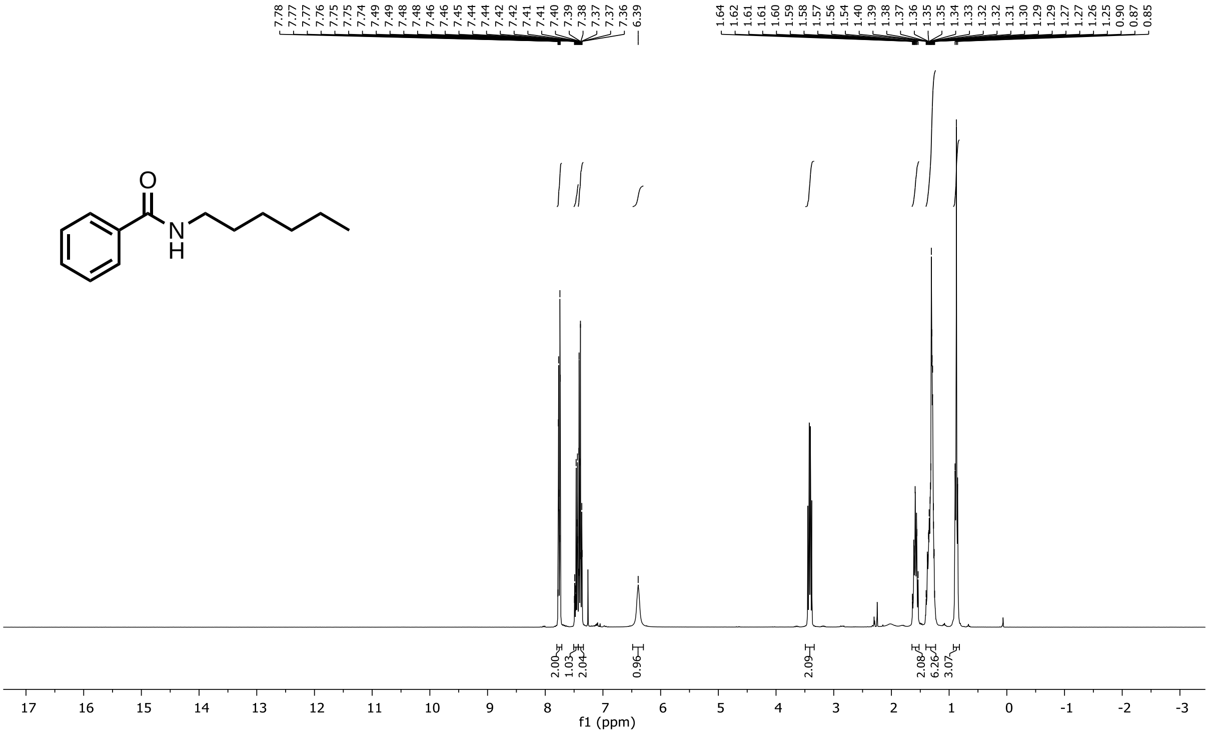

^{1}H NMR (300 MHz, Chloroform-*d*) *N*-hexylbenzamide (**4c**)

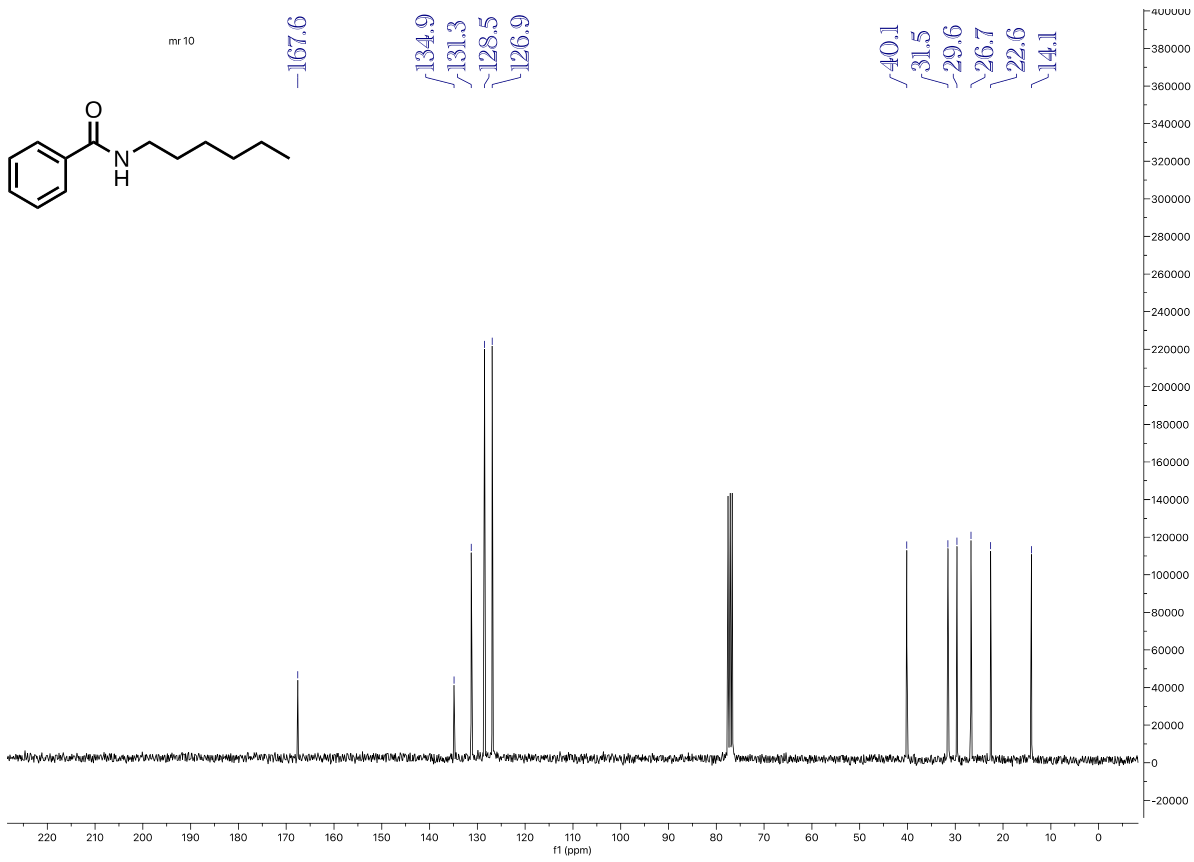

^{13}C{H} NMR (75 MHz, Chloroform-*d*) *N*-hexylbenzamide (**4c**)

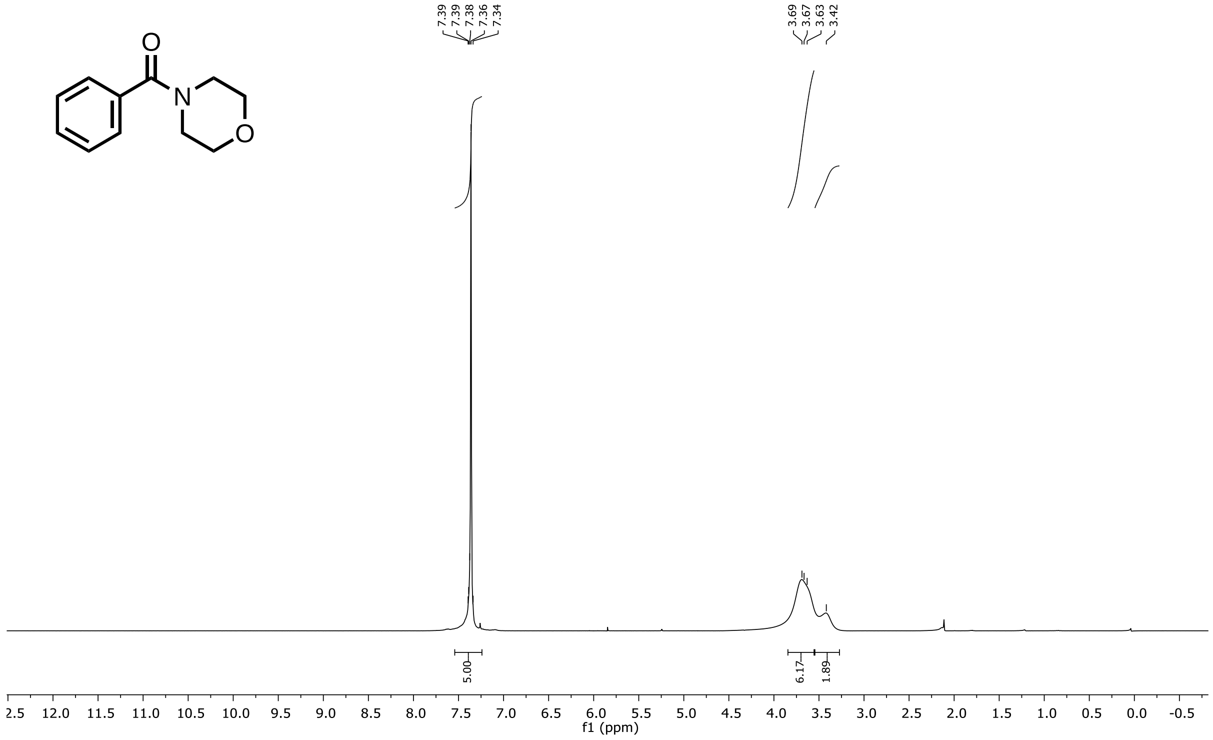

^{1}H NMR (400 MHz, Chloroform-*d*) morpholino(phenyl)methanone (**4d**)

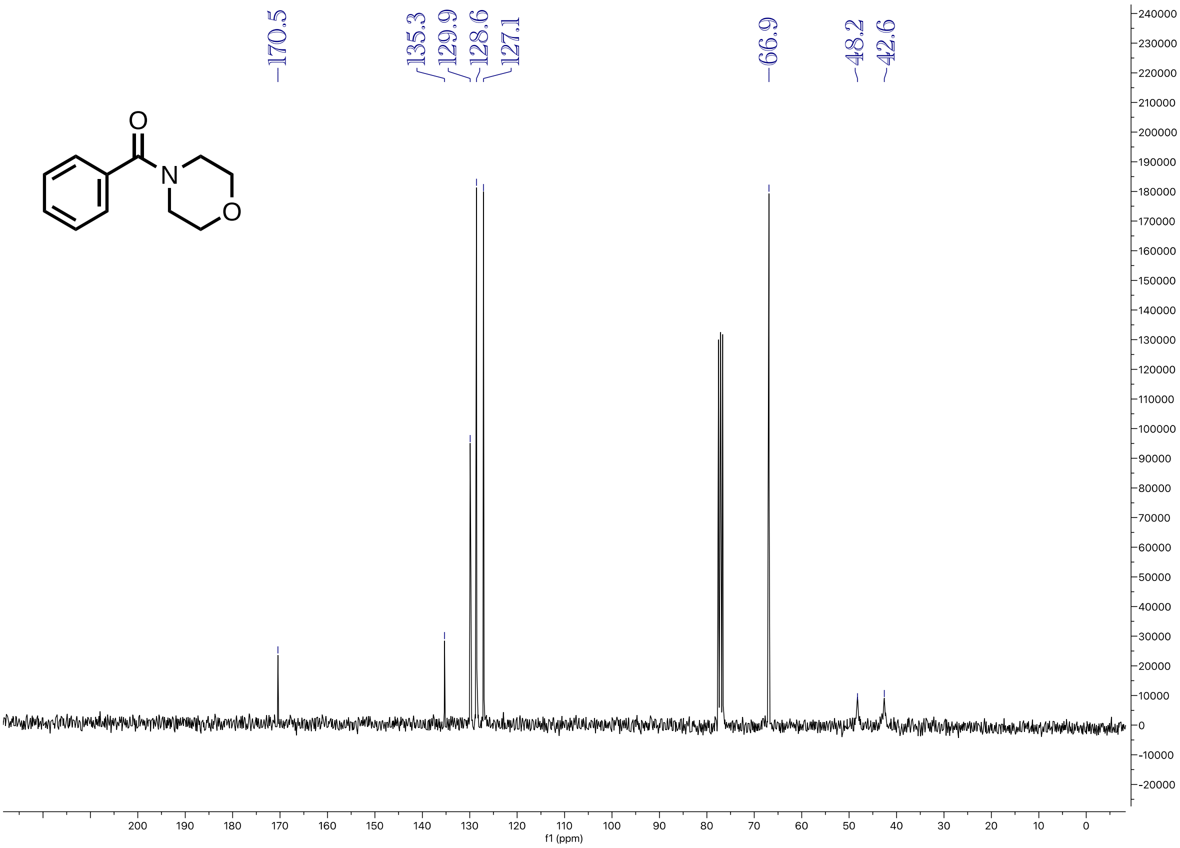

$^{13}C\{H\}$ NMR (75 MHz, Chloroform-*d*) morpholino(phenyl)methanone (**4d**)

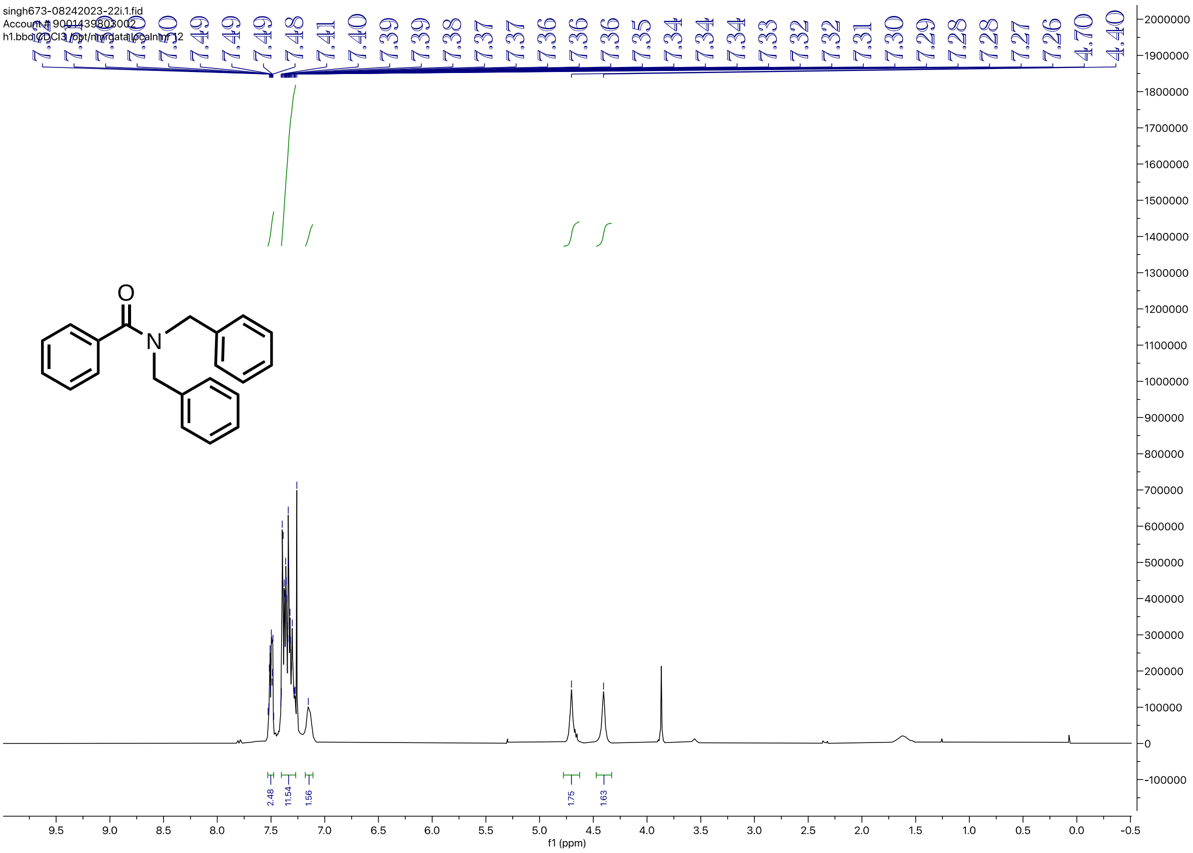

^{1}H NMR (300 MHz, Chloroform-*d*) *N*,*N*-dibenzylbenzamide (**4e**)

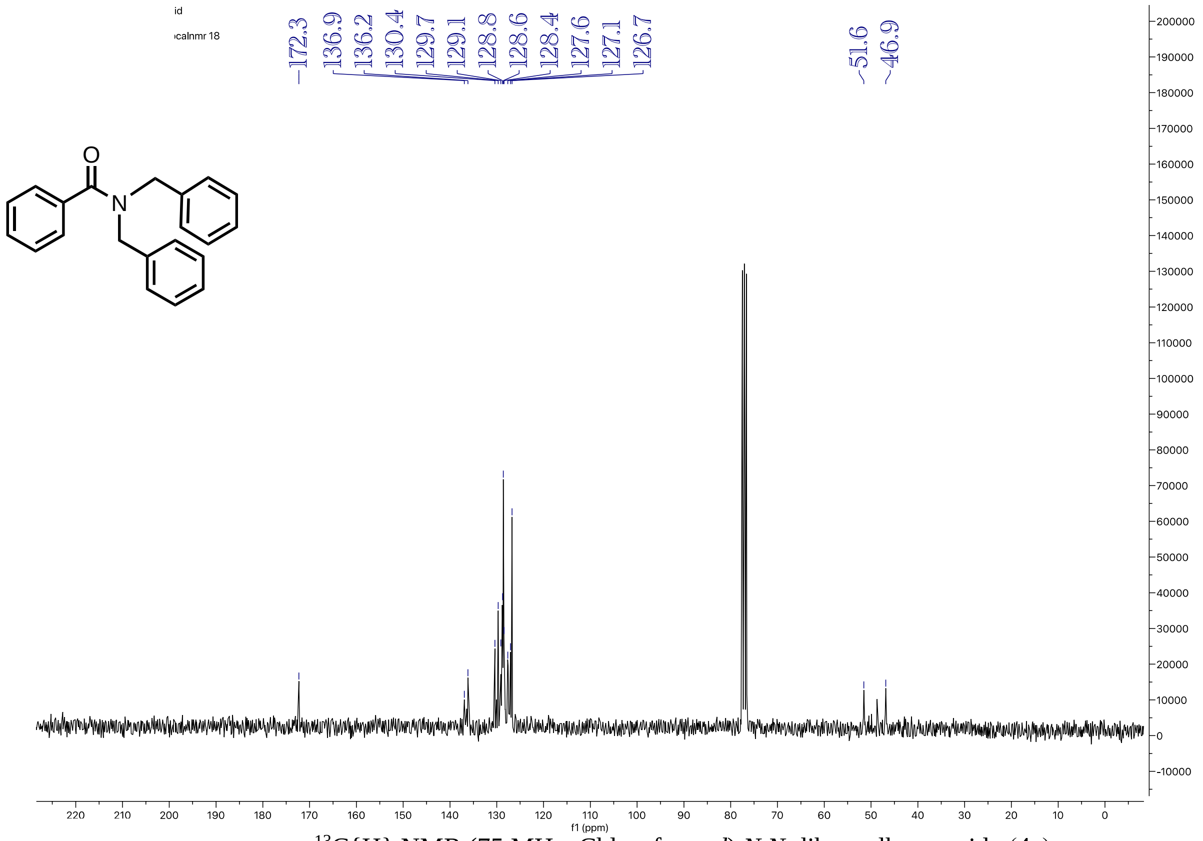

$^{13}C\{H\}$ NMR (75 MHz, Chloroform-*d*) *N*,*N*-dibenzylbenzamide (**4e**)

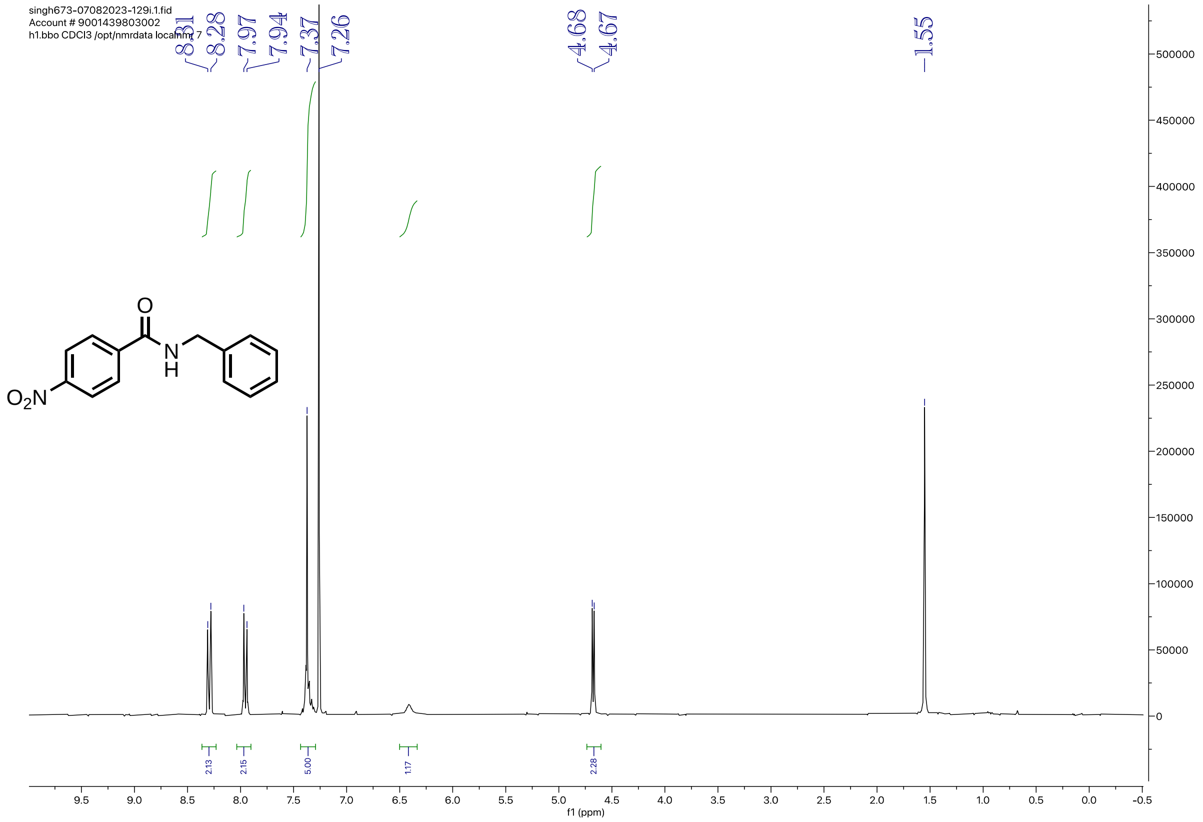

^{1}H NMR (300 MHz, Chloroform-*d*) *N*-benzyl-4-nitrobenzamide (**4f**)

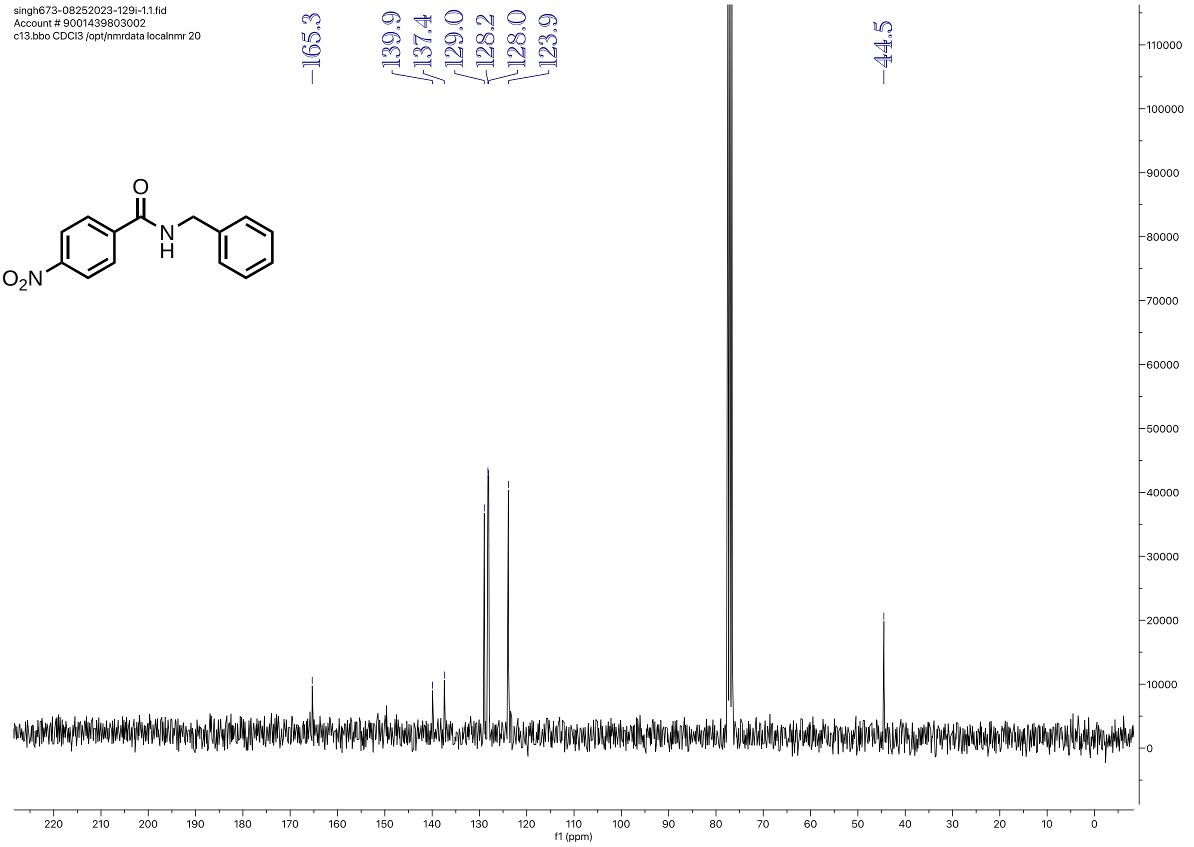

$^{13}C\{H\}$ NMR (75 MHz, Chloroform-*d*) *N*-benzyl-4-nitrobenzamide (**4f**)

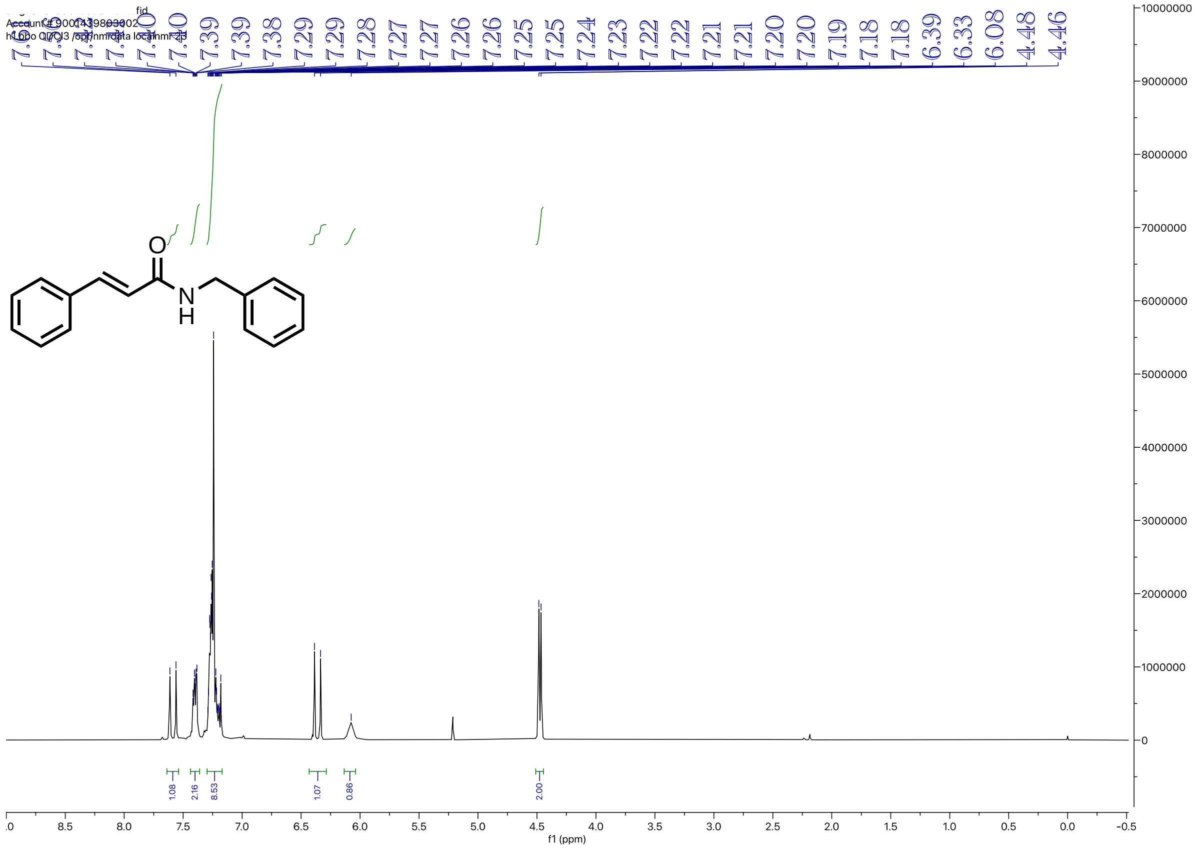

^{1}H NMR (300 MHz, Chloroform-*d*) *N*-benzylcinnamamide (**4g**)

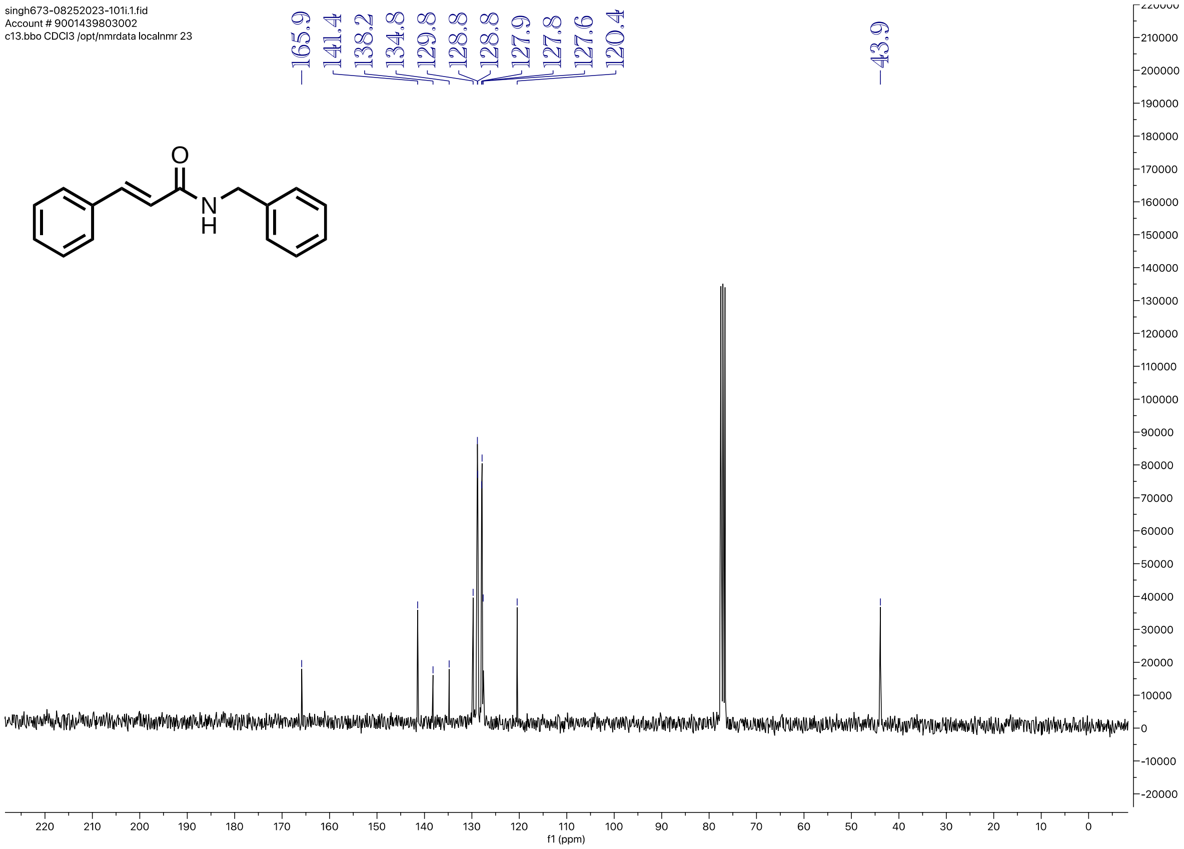

$^{13}C\{H\}$ NMR (75 MHz, Chloroform-*d*) *N*-benzylcinnamamide (**4g**)

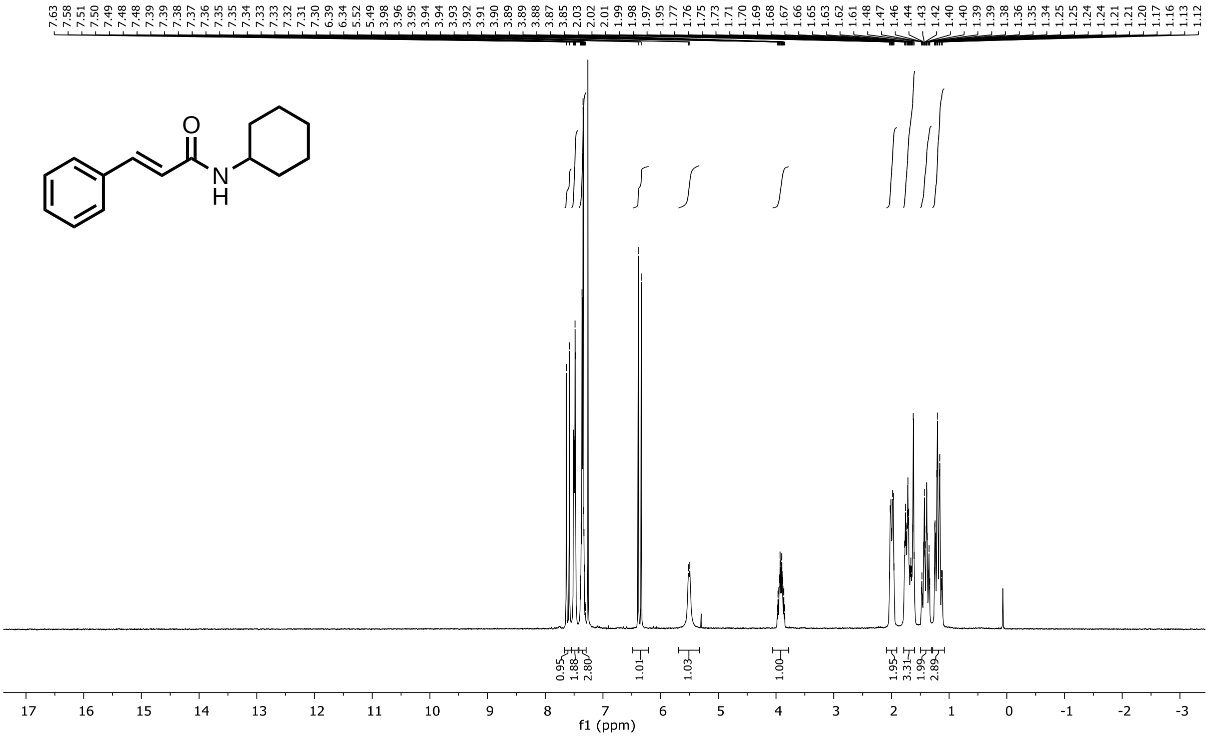

^{1}H NMR (300 MHz, Chloroform-*d*) *N*-cyclohexylcinnamamide (**4h**)

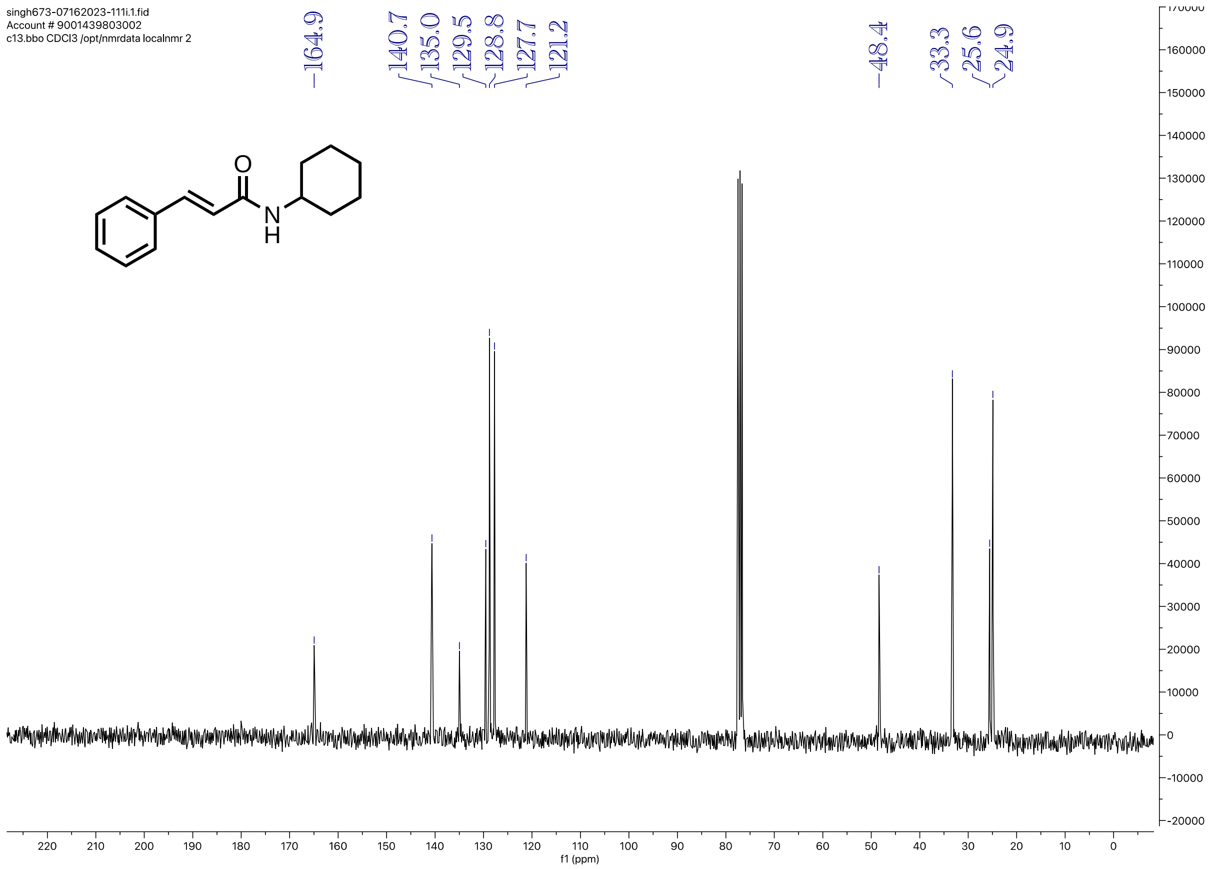

$^{13}C\{H\}$ NMR (75 MHz, Chloroform-*d*) *N*-cyclohexylcinnamamide (**4h**)

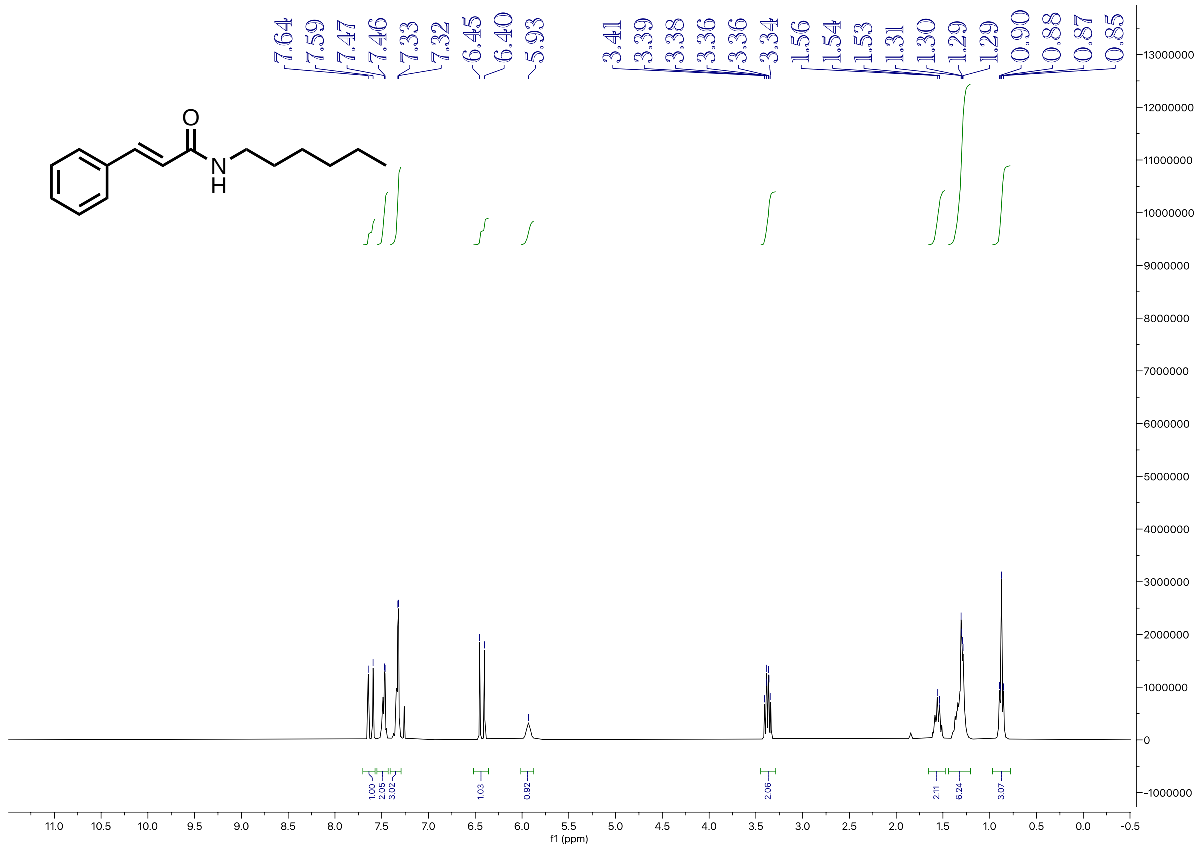

^{1}H NMR (300 MHz, Chloroform-*d*) *N*-hexylcinnamamide (**4i**)

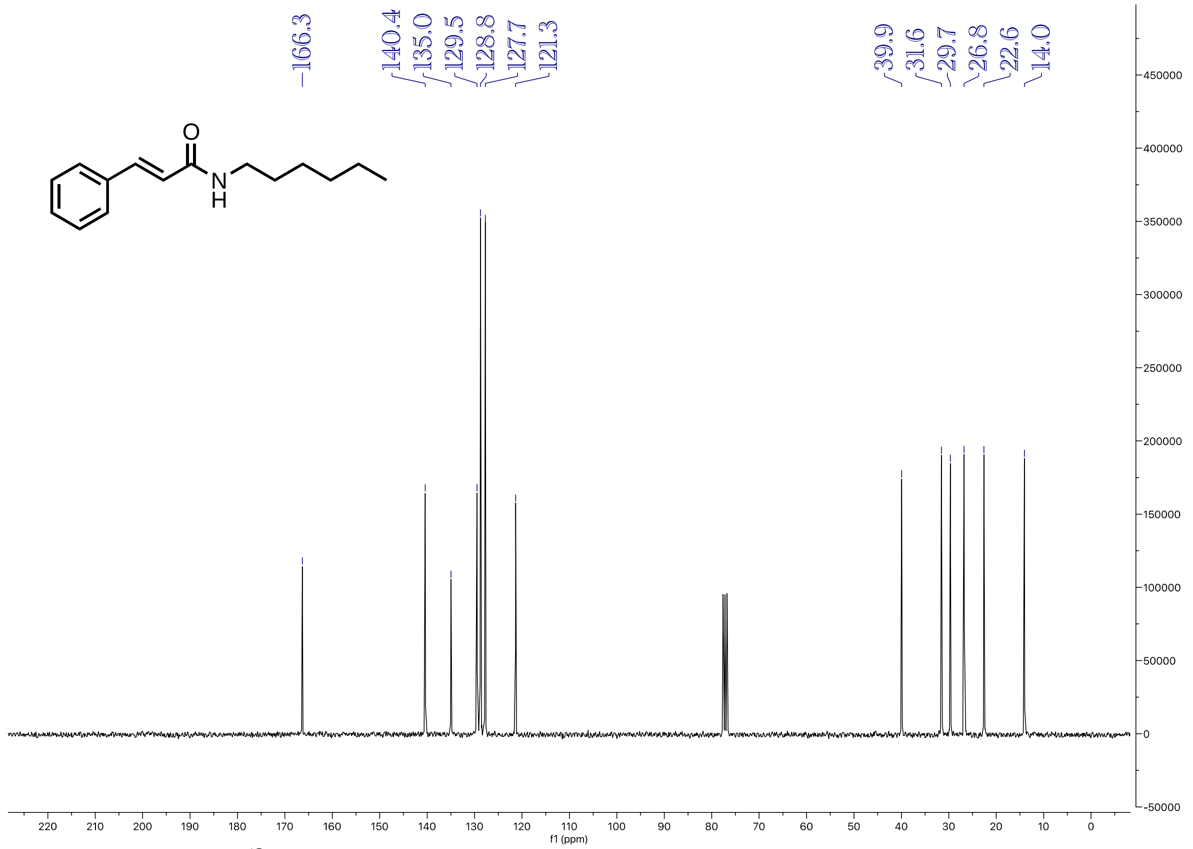

$^{13}C\{H\}$ NMR (75 MHz, Chloroform-*d*) *N*-hexylcinnamamide (**4i**)

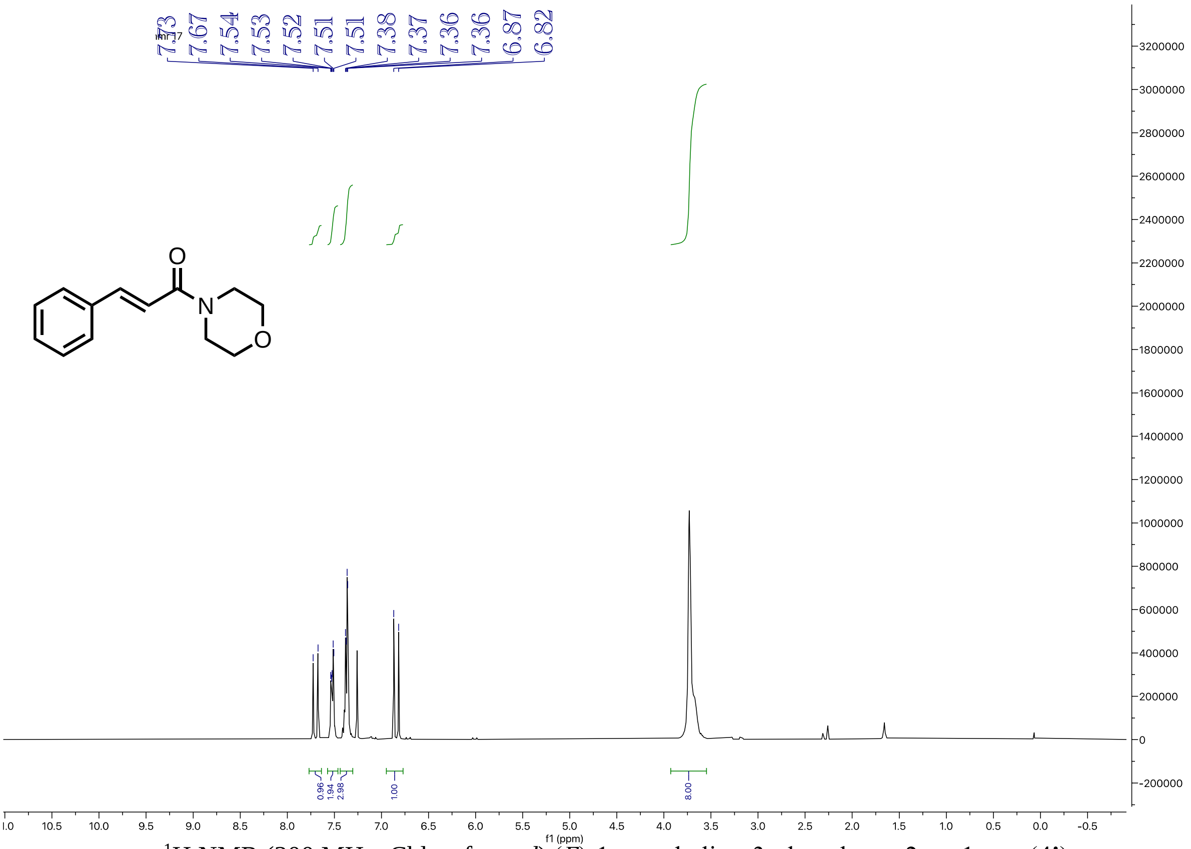

^{1}H NMR (300 MHz, Chloroform-*d*) (*E*)-1-morpholino-3-phenylprop-2-en-1-one (**4j**)

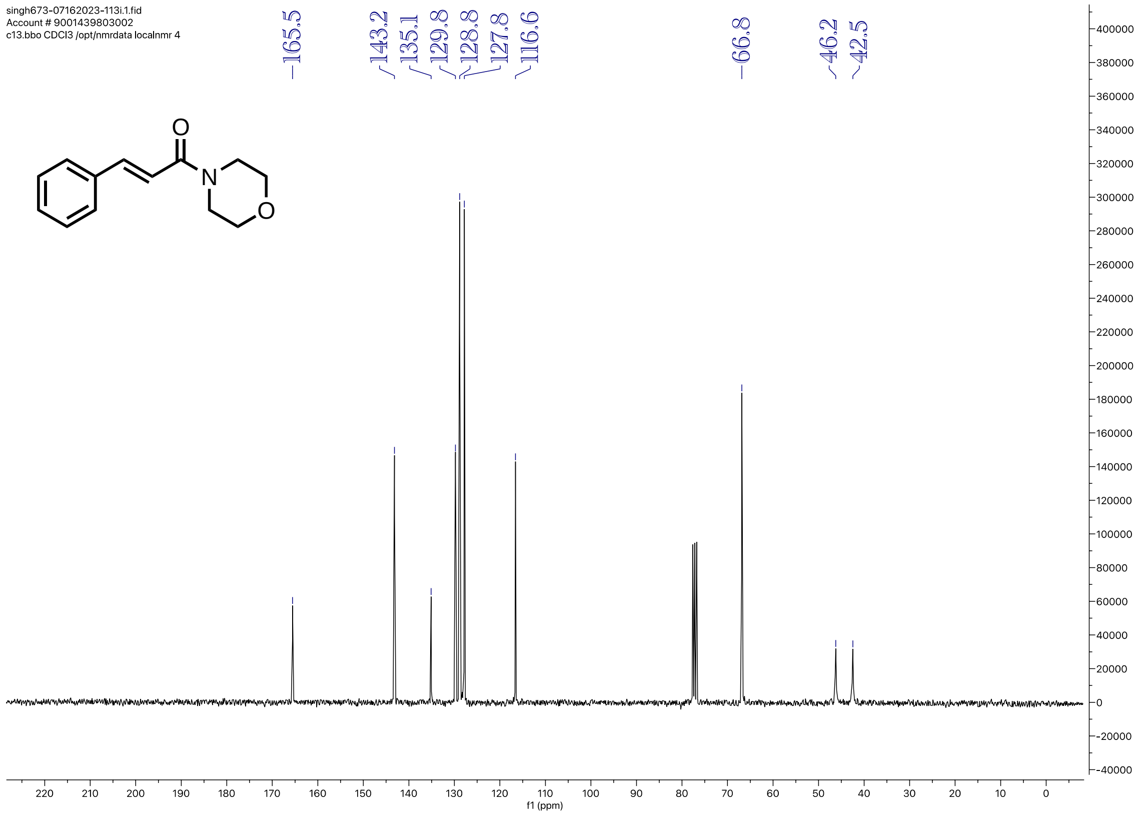

$^{13}C\{H\}$ NMR (75 MHz, Chloroform-*d*) (*E*)-1-morpholino-3-phenylprop-2-en-1-one (**4j**)

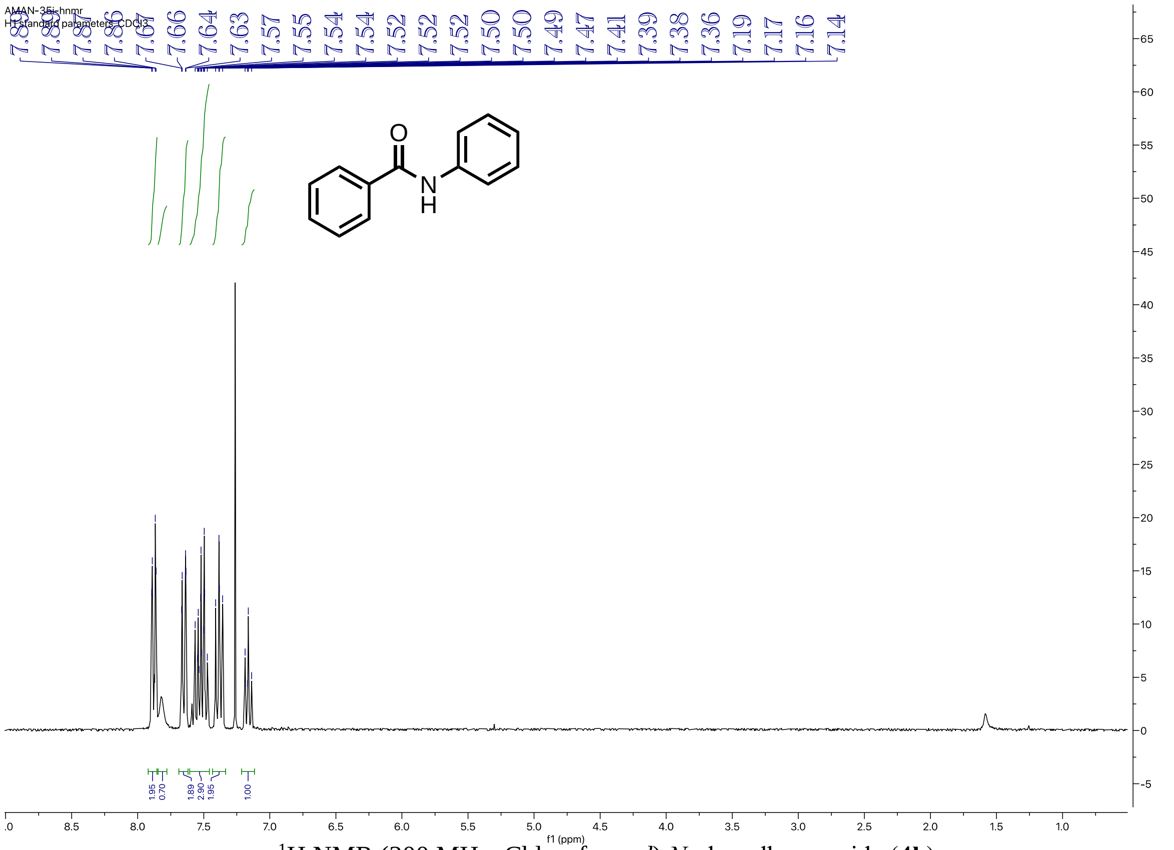

^{1}H NMR (300 MHz, Chloroform-*d*) *N*-phenylbenzamide (**4k**)

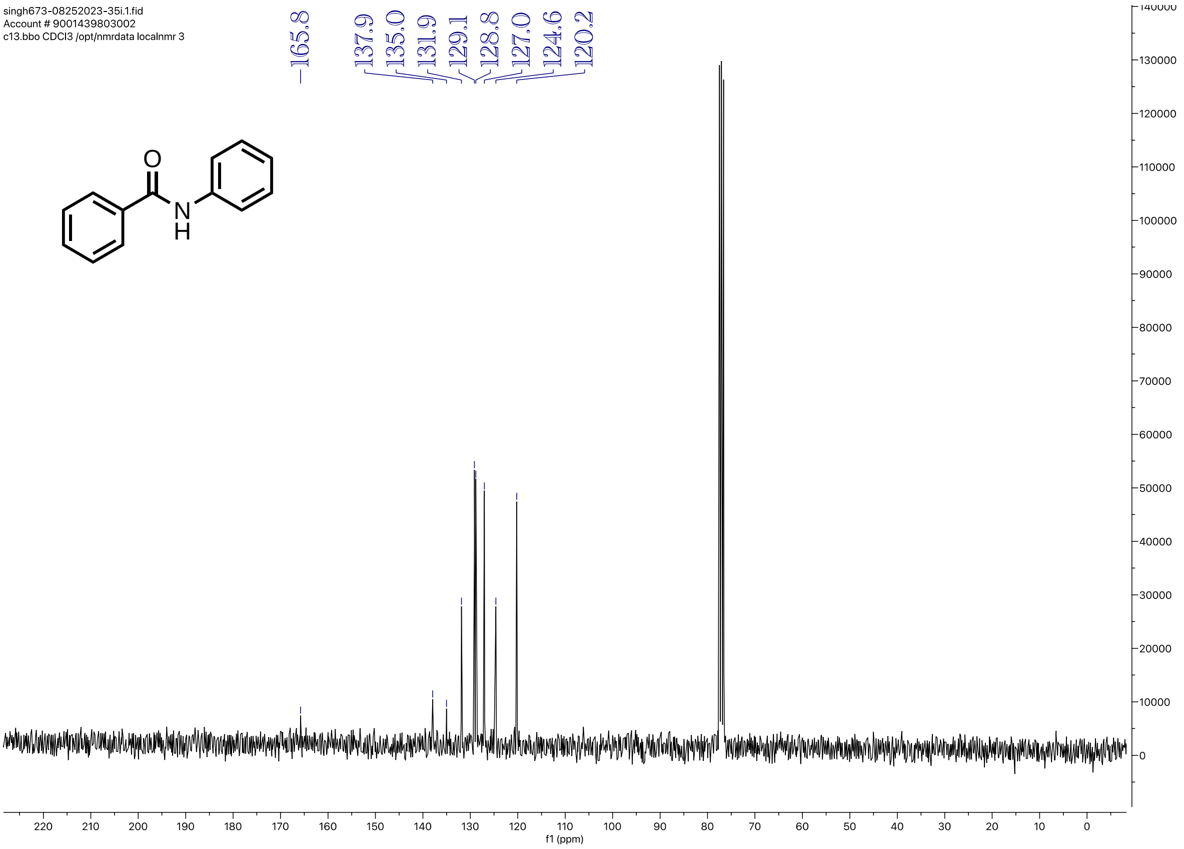

$^{13}C\{H\}$ NMR (75 MHz, Chloroform-*d*) *N*-phenylbenzamide (**4k**)

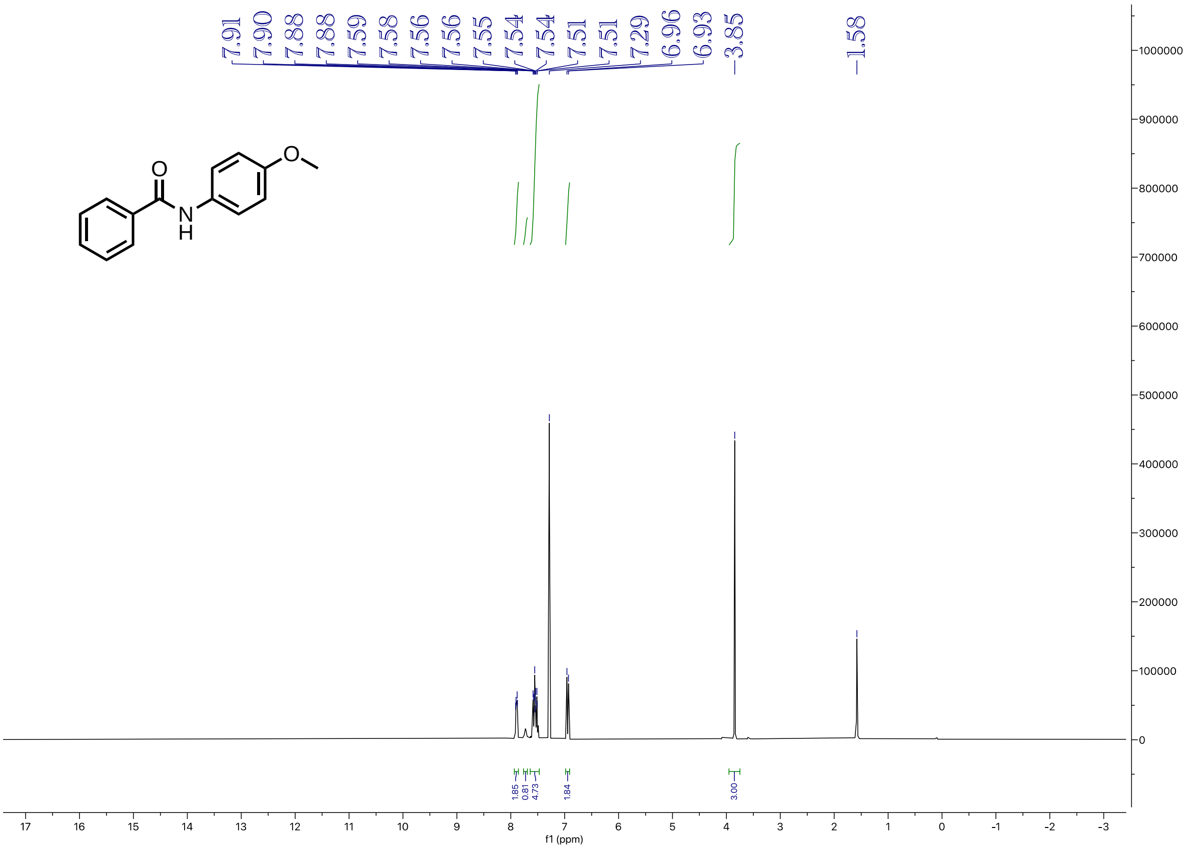

^{1}H NMR (300 MHz, Chloroform-*d*) N-(4-methoxyphenyl)benzamide (**4l**)

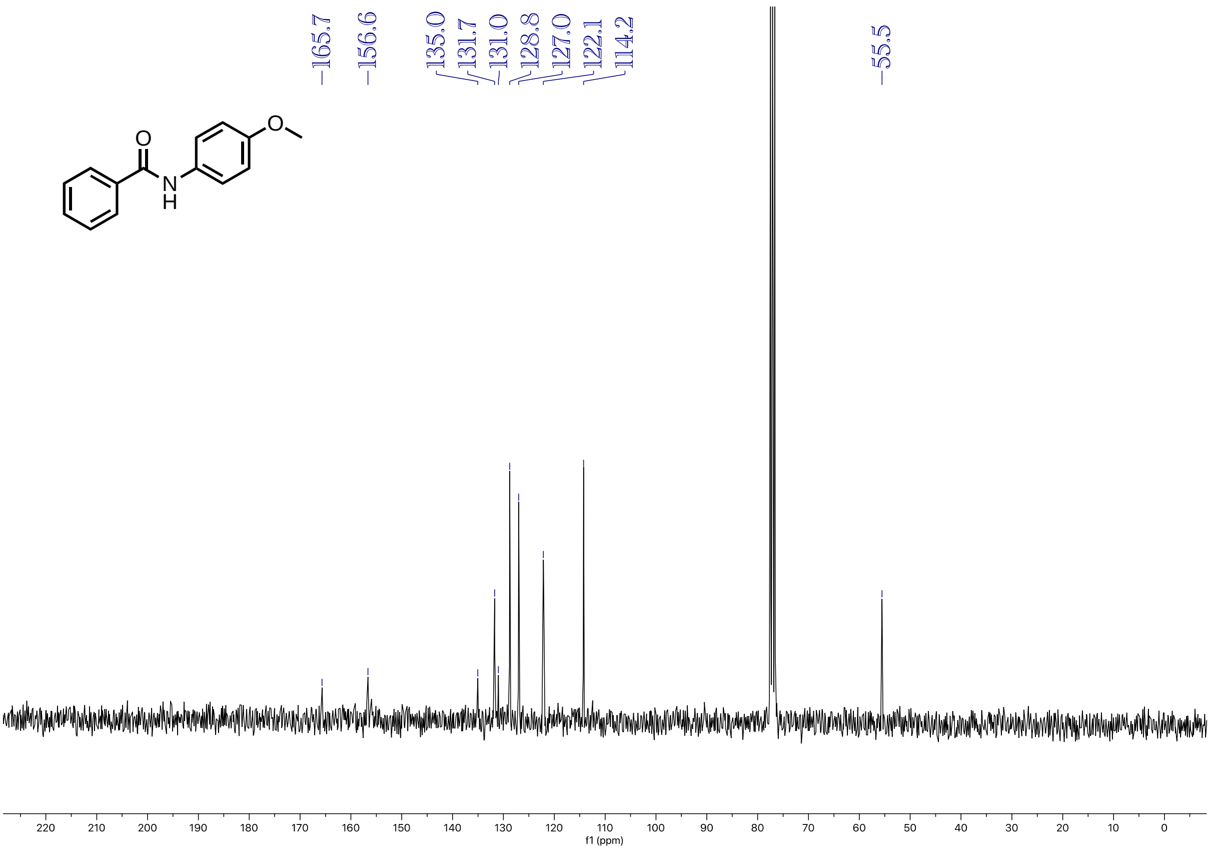

$^{13}C\{H\}$ NMR (75 MHz, Chloroform-*d*) N-(4-methoxyphenyl)benzamide (**4l**)

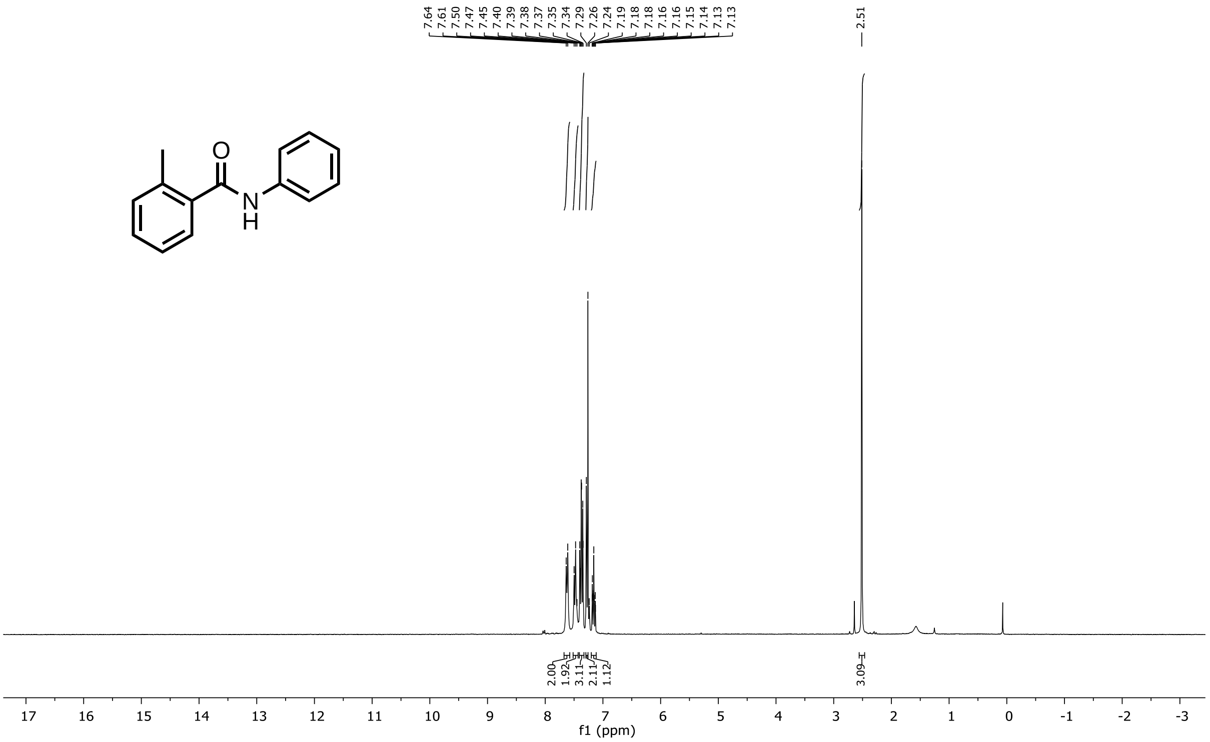

^{1}H NMR (300 MHz, Chloroform-*d*) 2-methyl-*N*-phenylbenzamide (**4m**)

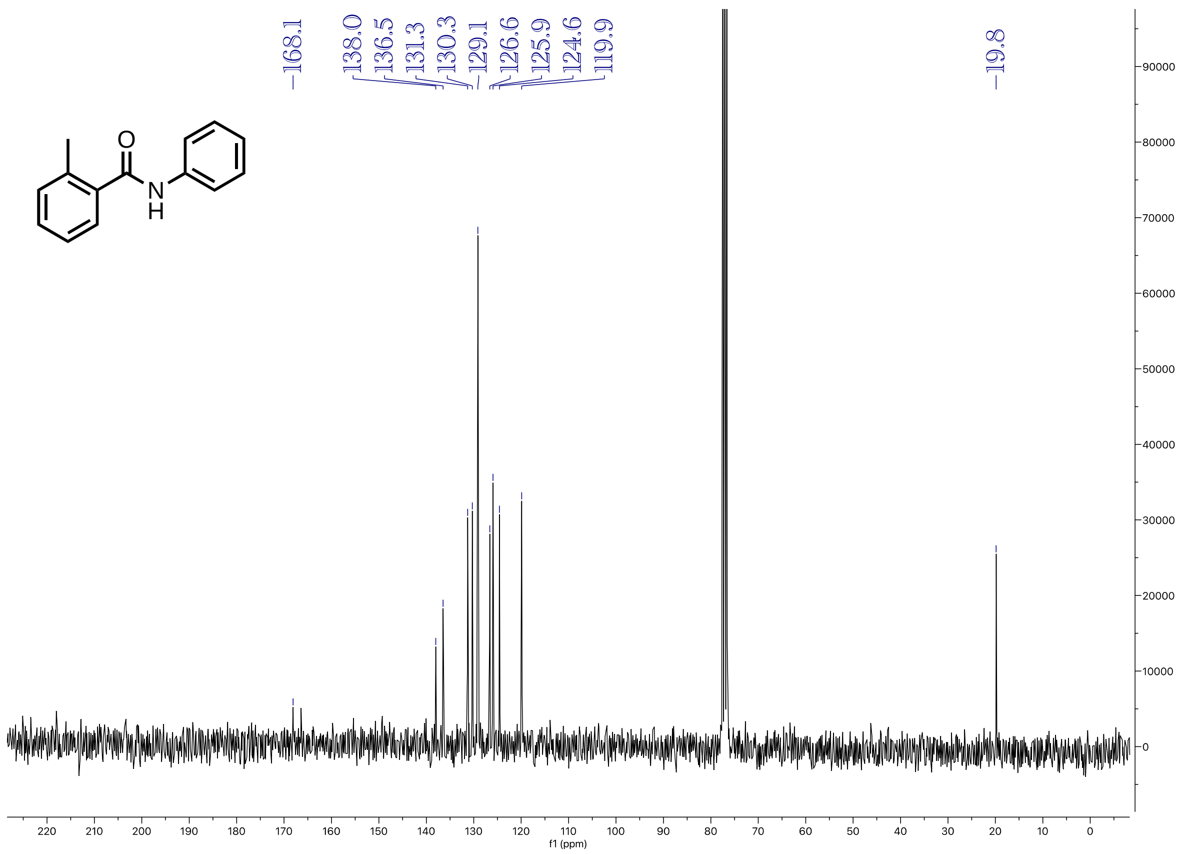

$^{13}C\{H\}$ NMR (75 MHz, Chloroform-*d*) 2-methyl-*N*-phenylbenzamide (**4m**)

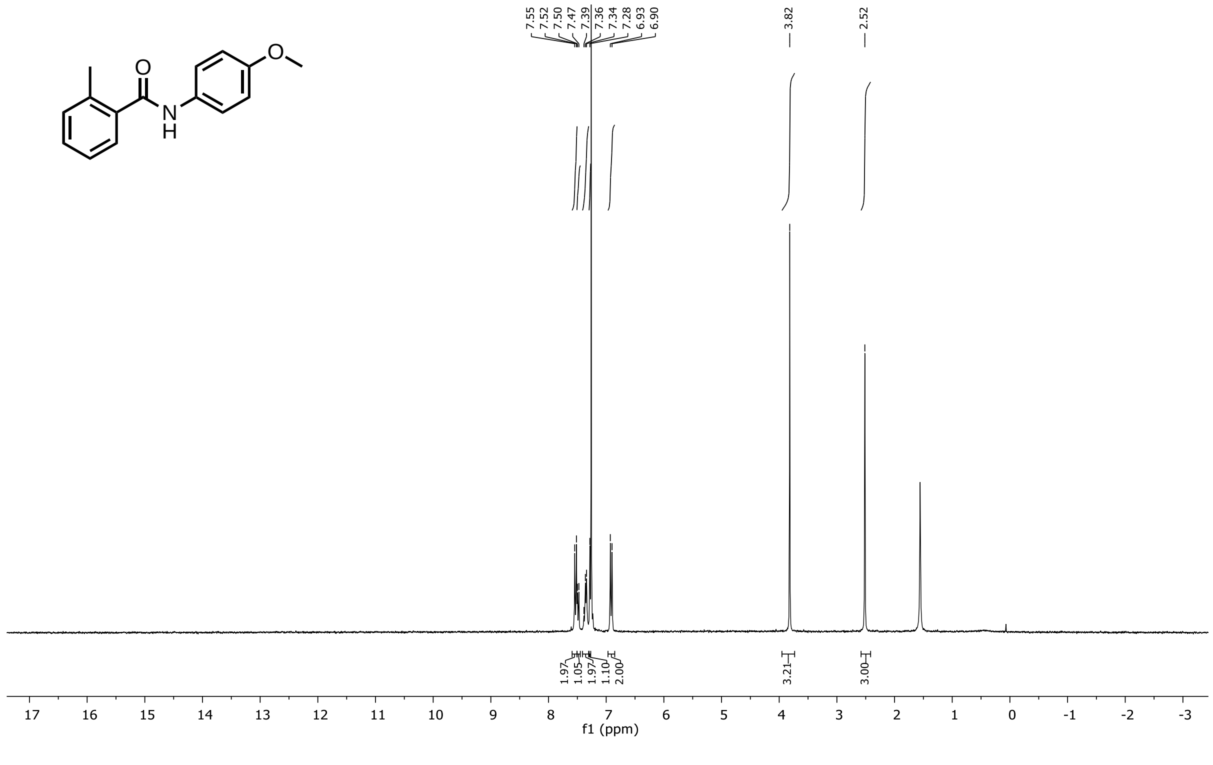

^{1}H NMR (300 MHz, Chloroform-*d*) *N*-(4-methoxyphenyl)-2-methylbenzamide (**4n**)

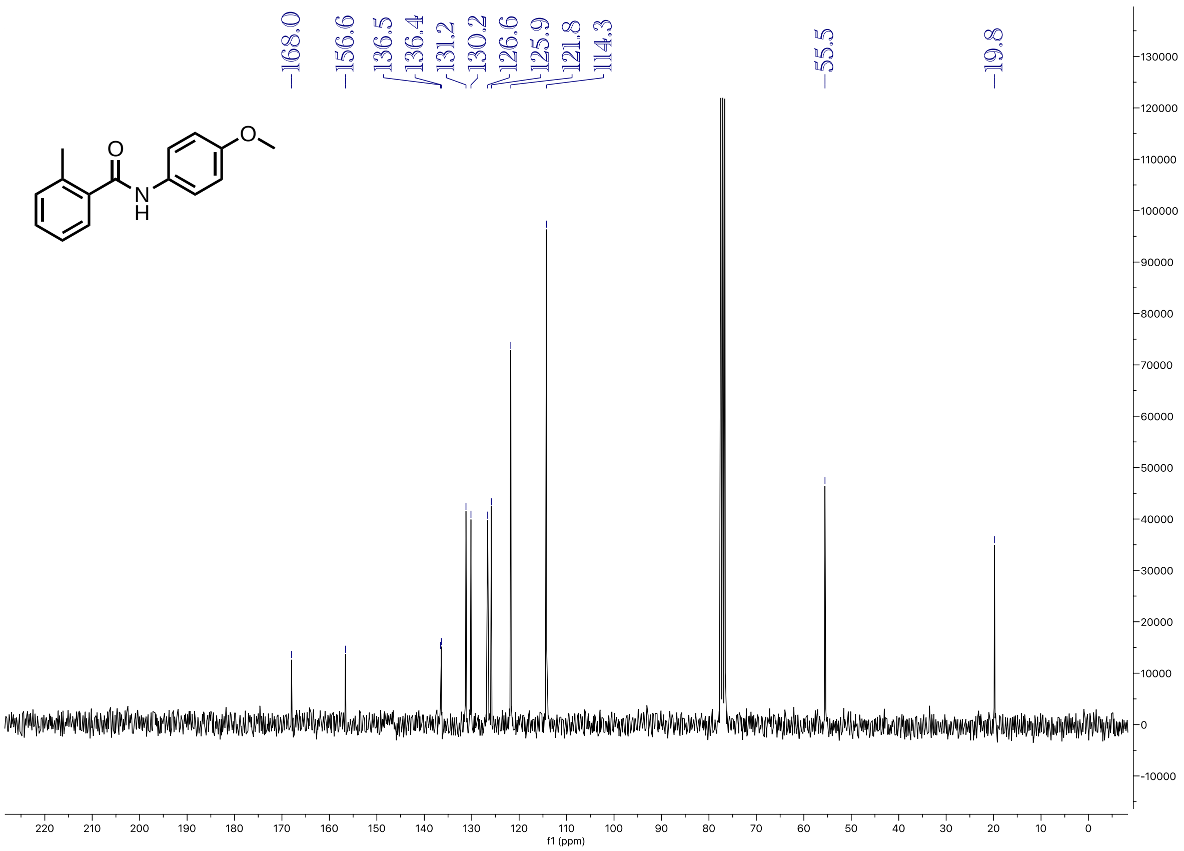

$^{13}C\{H\}$ NMR (75 MHz, Chloroform-*d*) *N*-(4-methoxyphenyl)-2-methylbenzamide (**4n**)

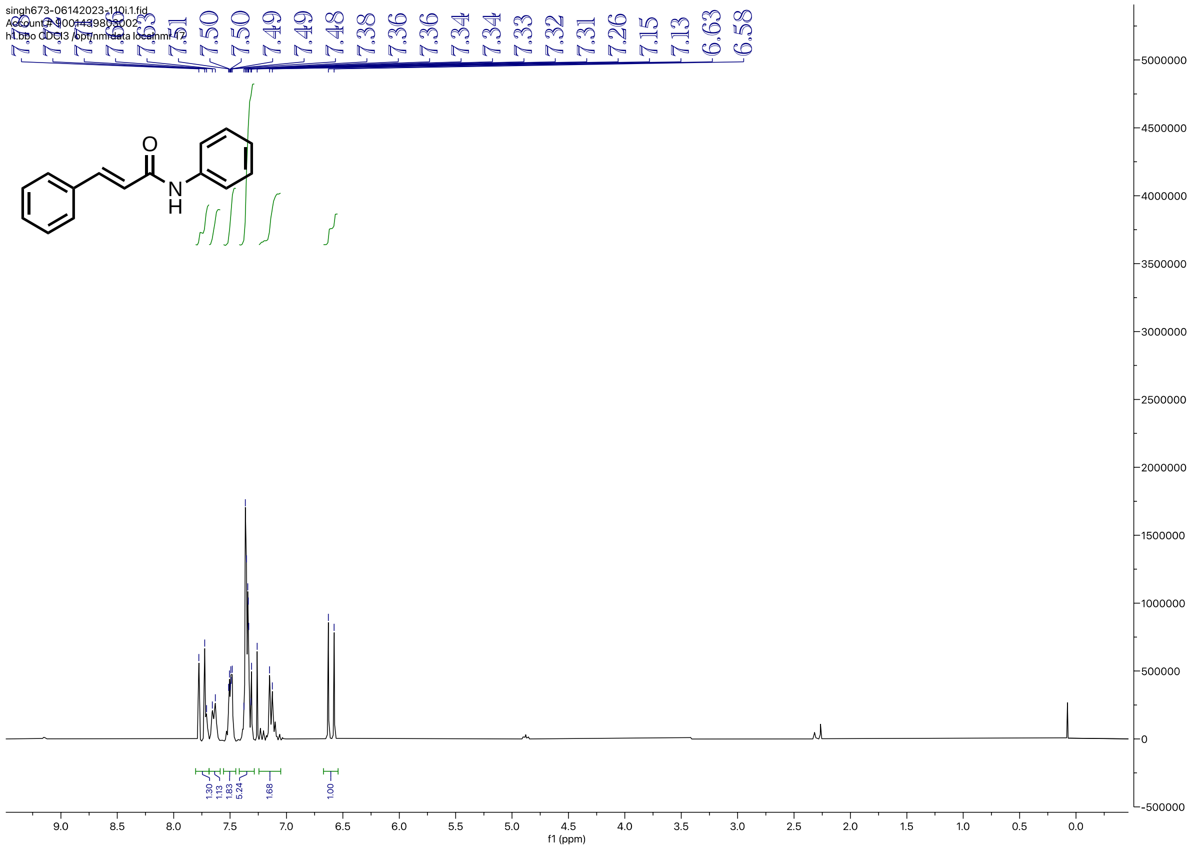

^{1}H NMR (300 MHz, Chloroform-*d*) *N*-phenylcinnamamide (**4o**)

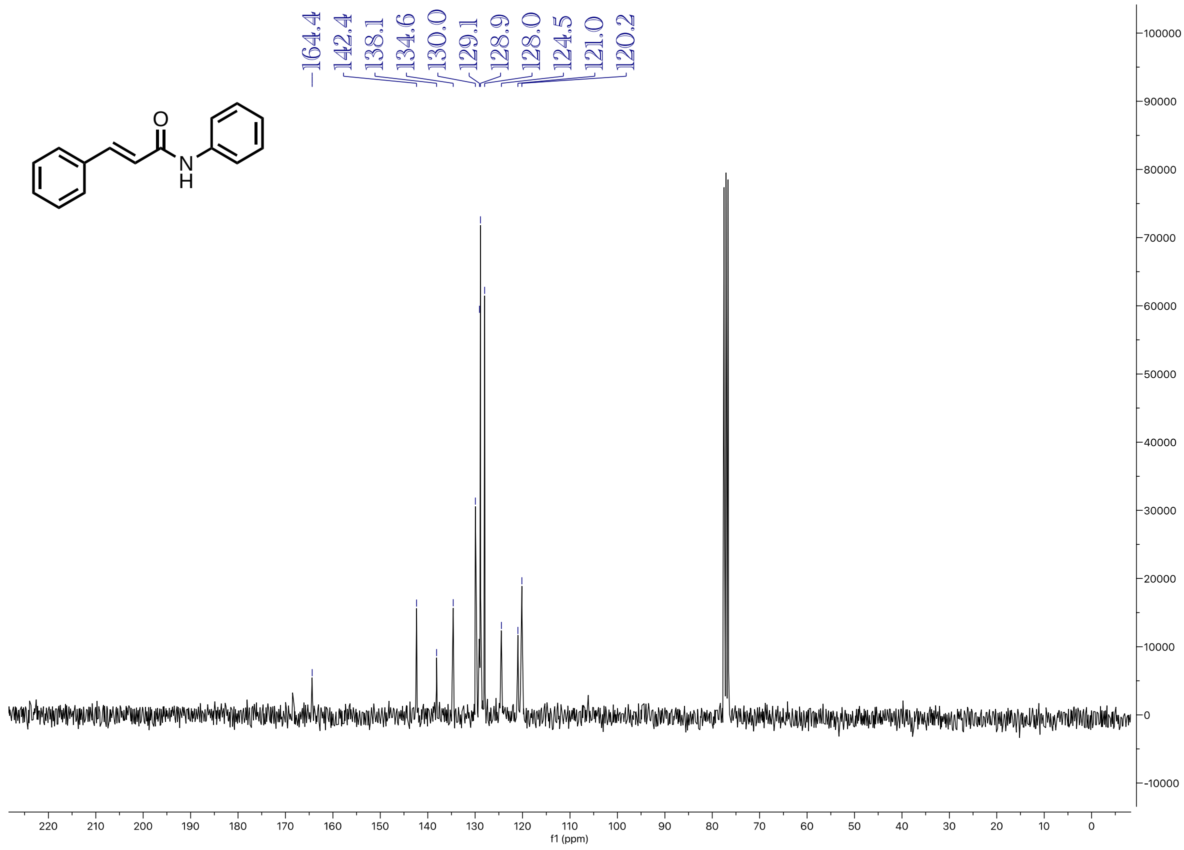

^{13}C{H} NMR (75 MHz, Chloroform-*d*) *N*-phenylcinnamamide (**4o**)

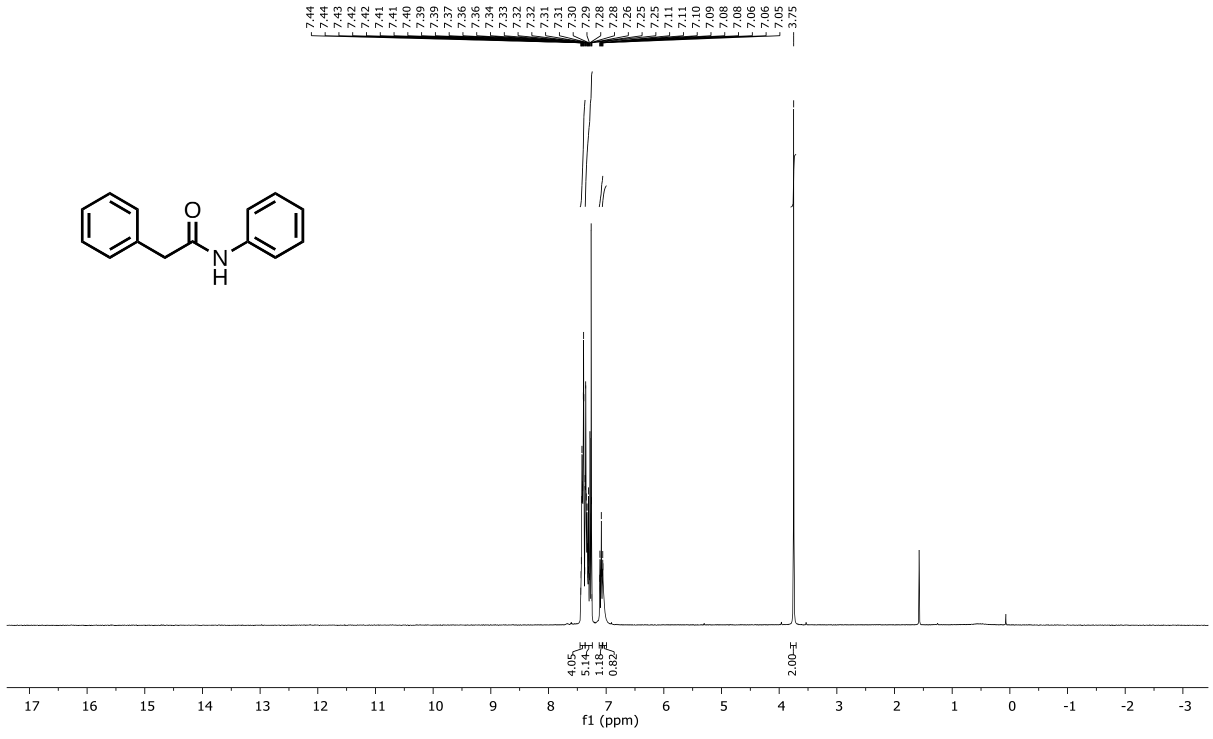

^{1}H NMR (300 MHz, Chloroform-*d*) *N*,2-diphenylacetamide (**4p**)

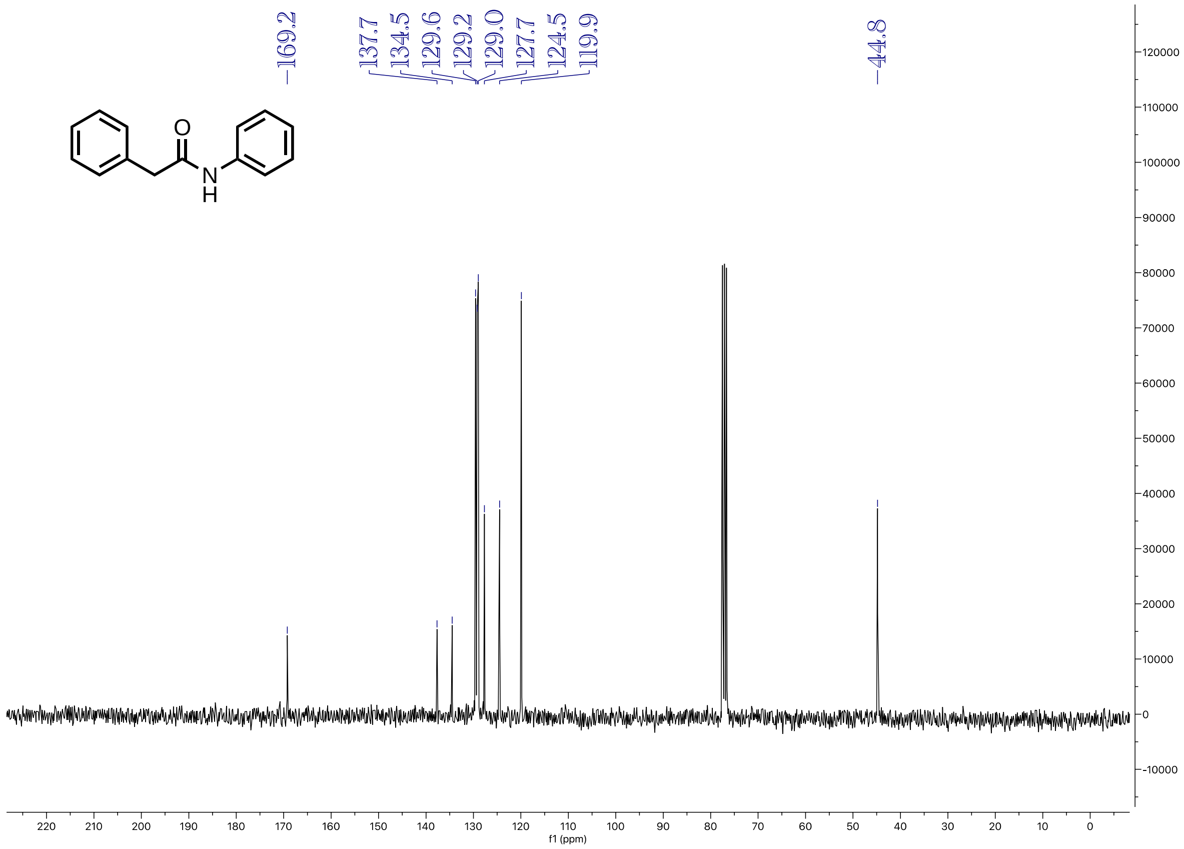

$^{13}C\{H\}$ NMR (75 MHz, Chloroform-*d*) *N*,2-diphenylacetamide (**4p**)

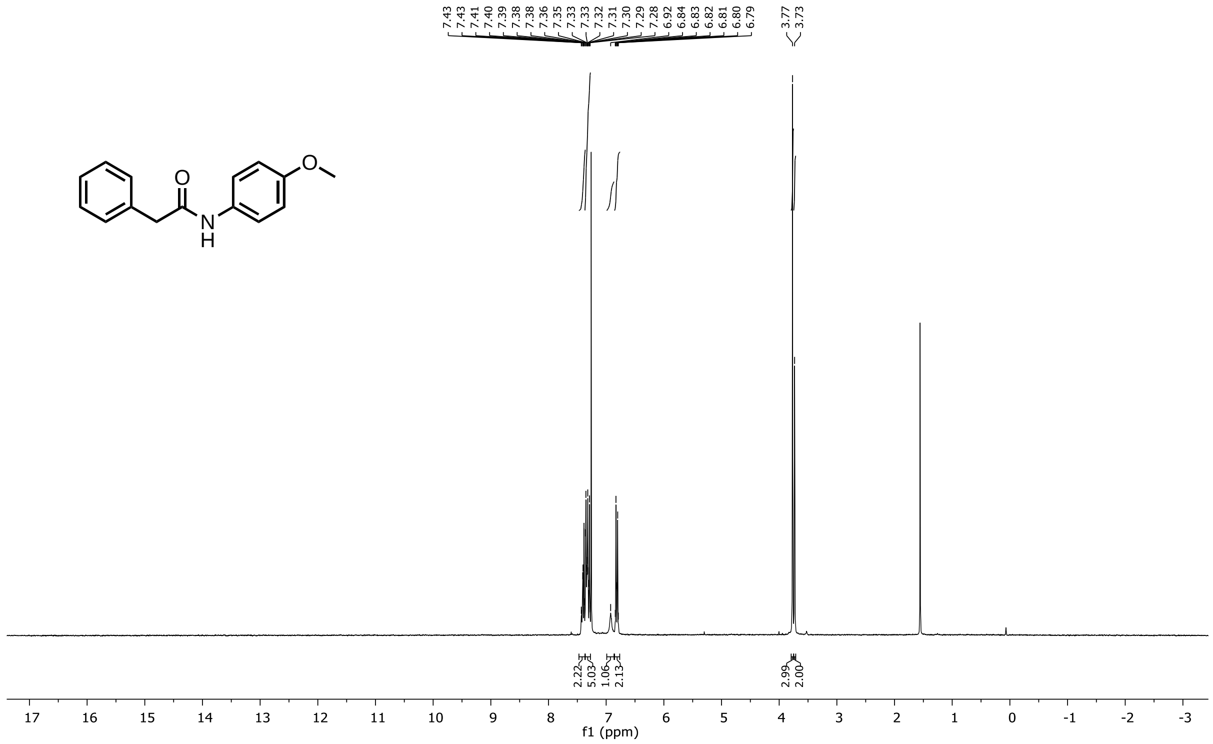

^{1}H NMR (300 MHz, Chloroform-*d*) *N*-(4-methoxyphenyl)-2-phenylacetamide (**4q**)

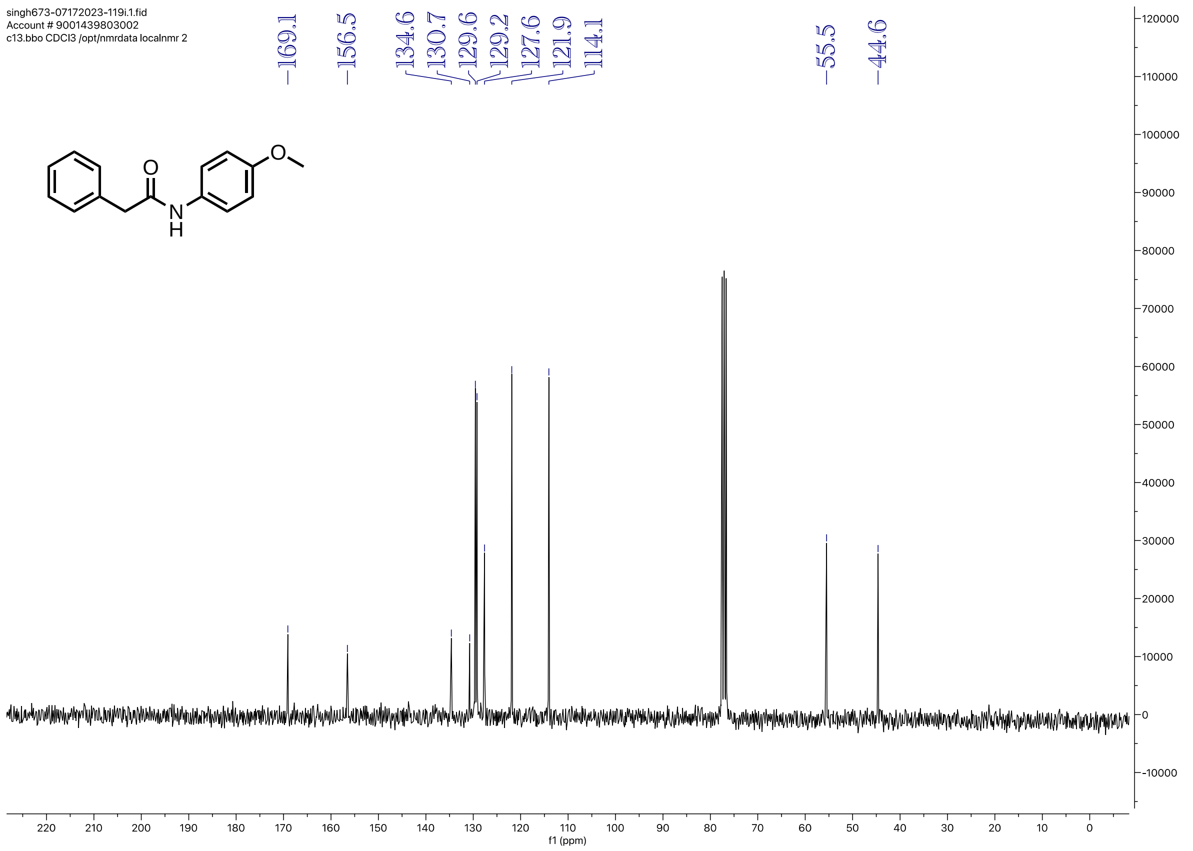

$^{13}C\{H\}$ NMR (75 MHz, Chloroform-*d*) *N*-(4-methoxyphenyl)-2-phenylacetamide (**4q**)

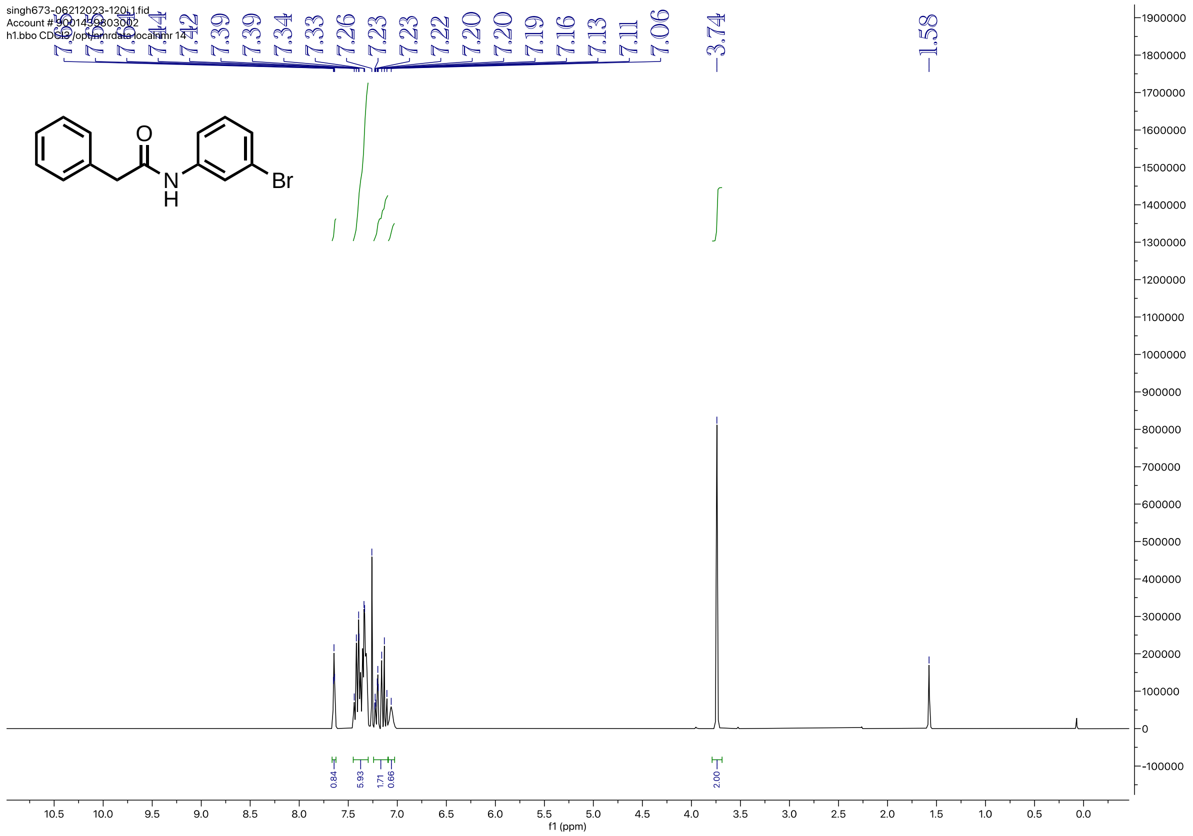

^{1}H NMR (300 MHz, Chloroform-*d*) *N*-(3-bromophenyl)-2-phenylacetamide (**4r**)

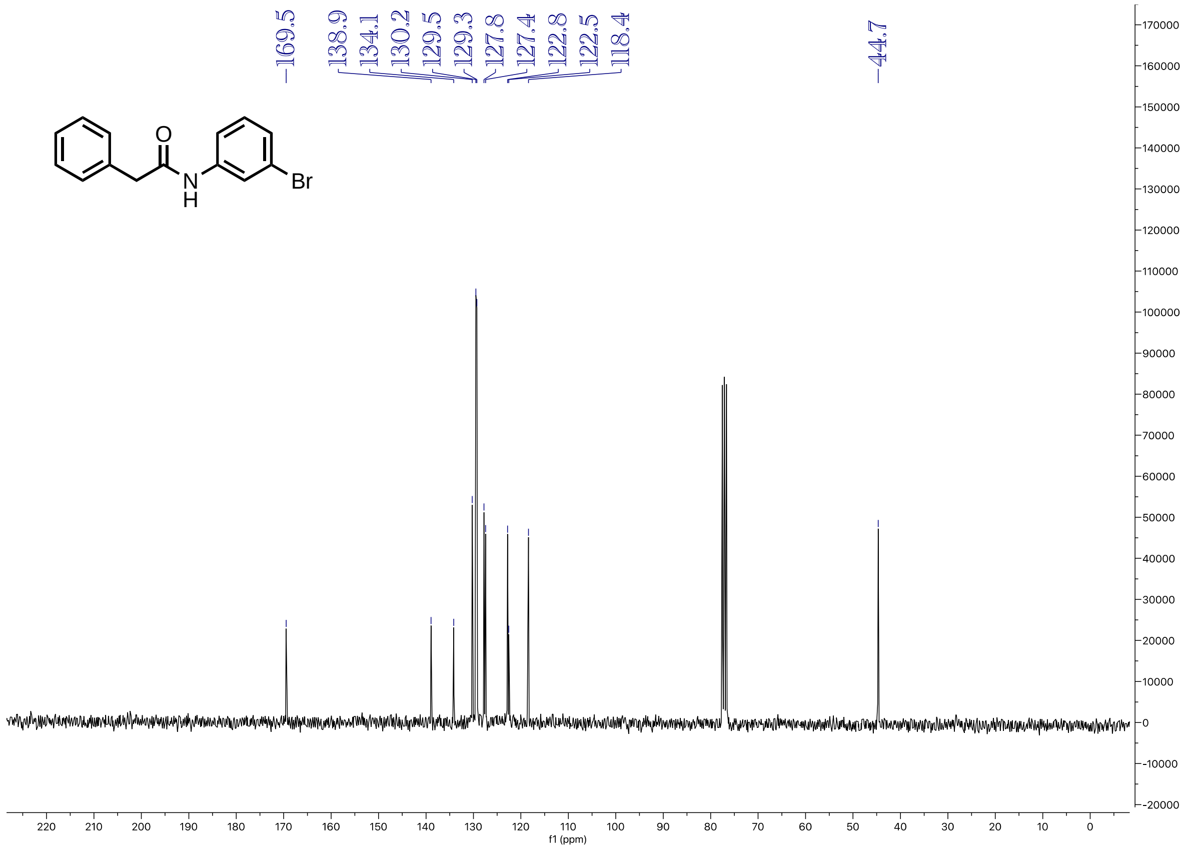

$^{13}C\{H\}$ NMR (75 MHz, Chloroform-*d*) *N*-(3-bromophenyl)-2-phenylacetamide (**4r**)

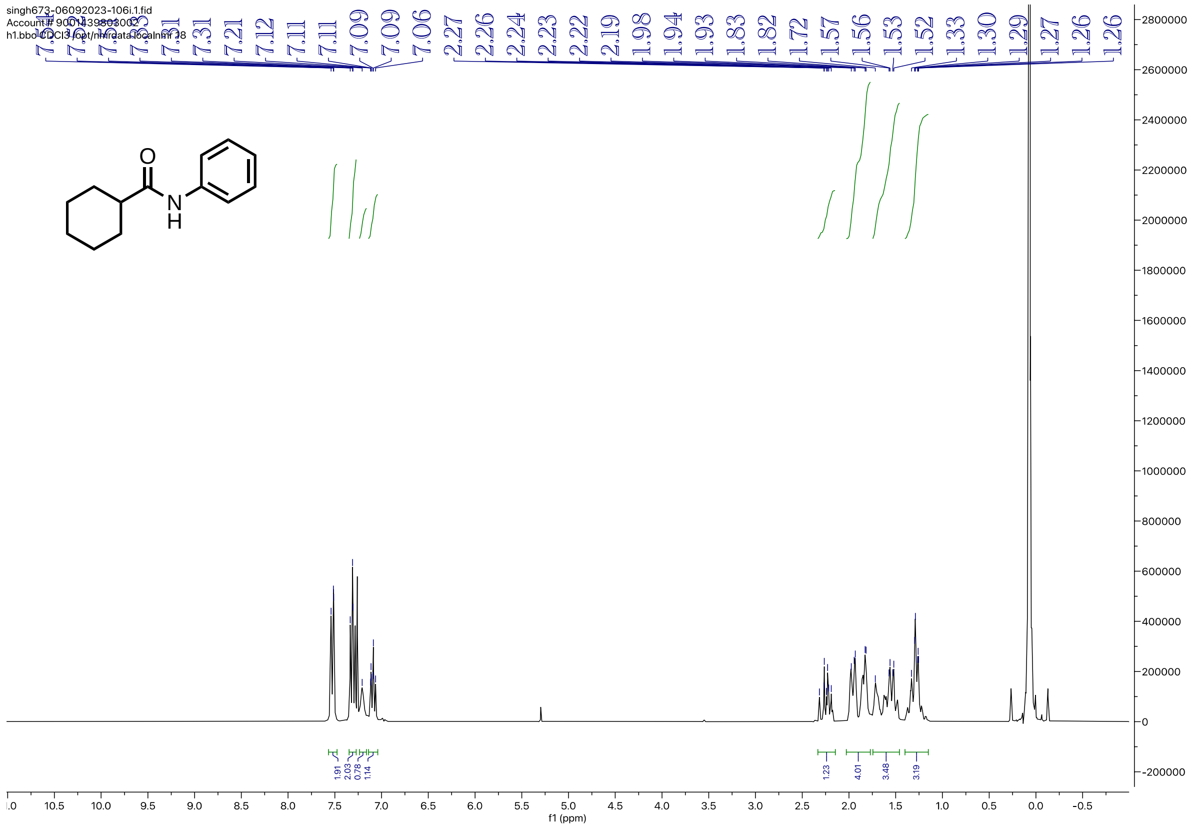

^{1}H NMR (300 MHz, Chloroform-*d*) *N*-phenylcyclohexanecarboxamide (**4s**)

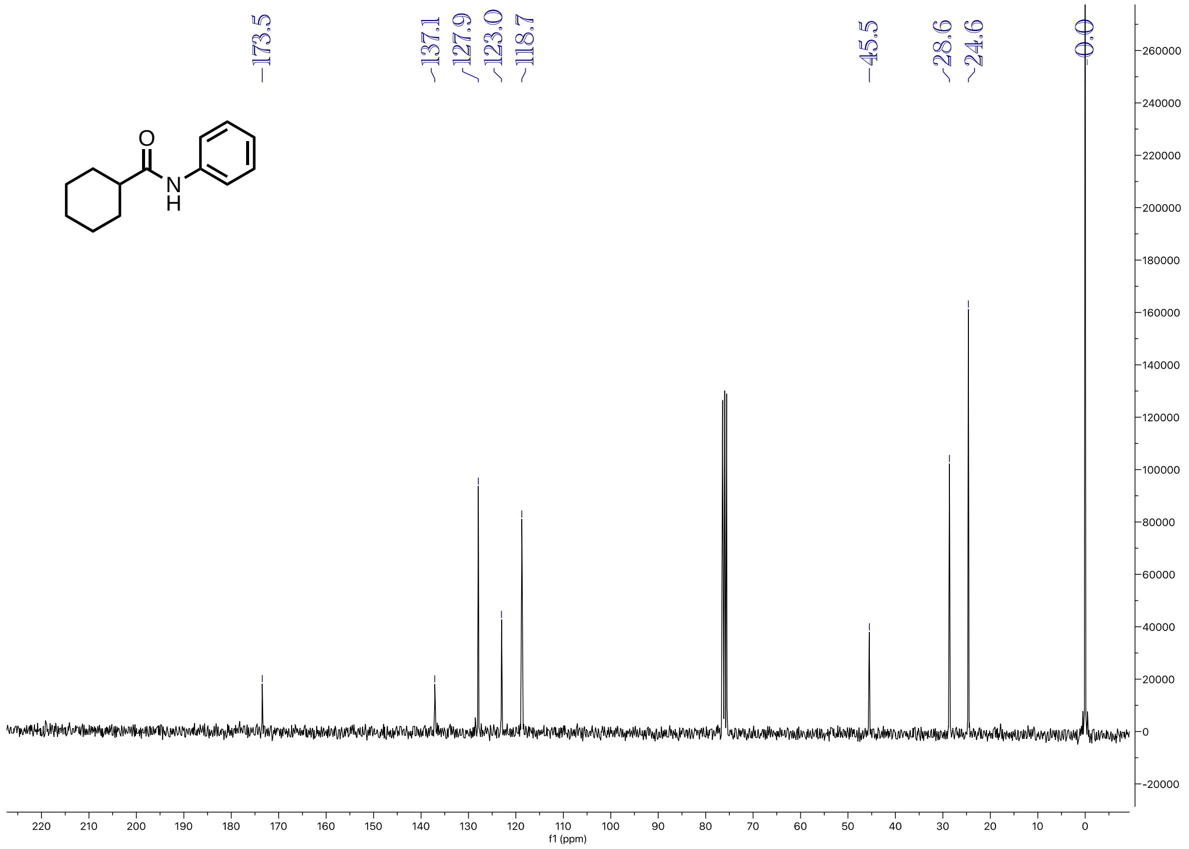

$^{13}C\{H\}$ NMR (75 MHz, Chloroform-*d*) *N*-phenylcyclohexanecarboxamide (**4s**)

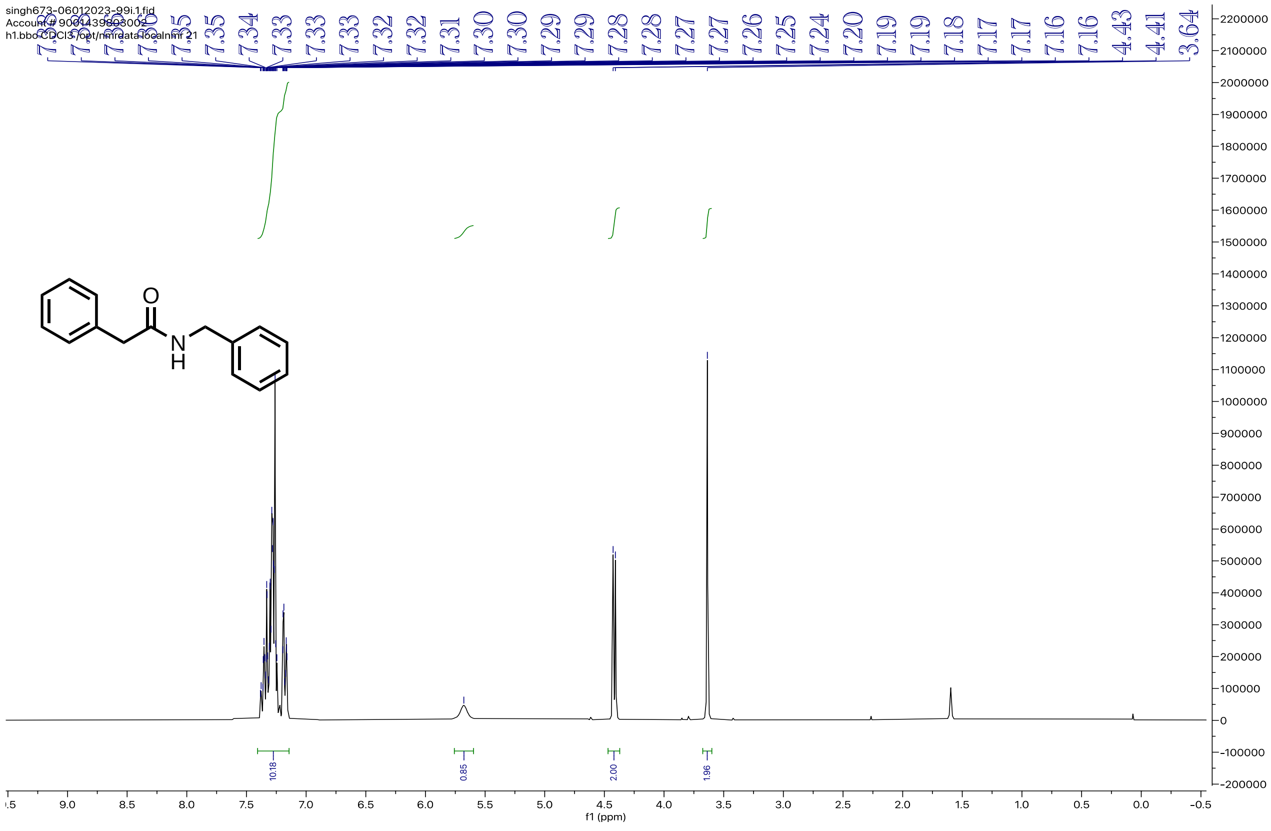

^{1}H NMR (300 MHz, Chloroform-*d*) *N*-benzyl-2-phenylacetamide (**4t**)

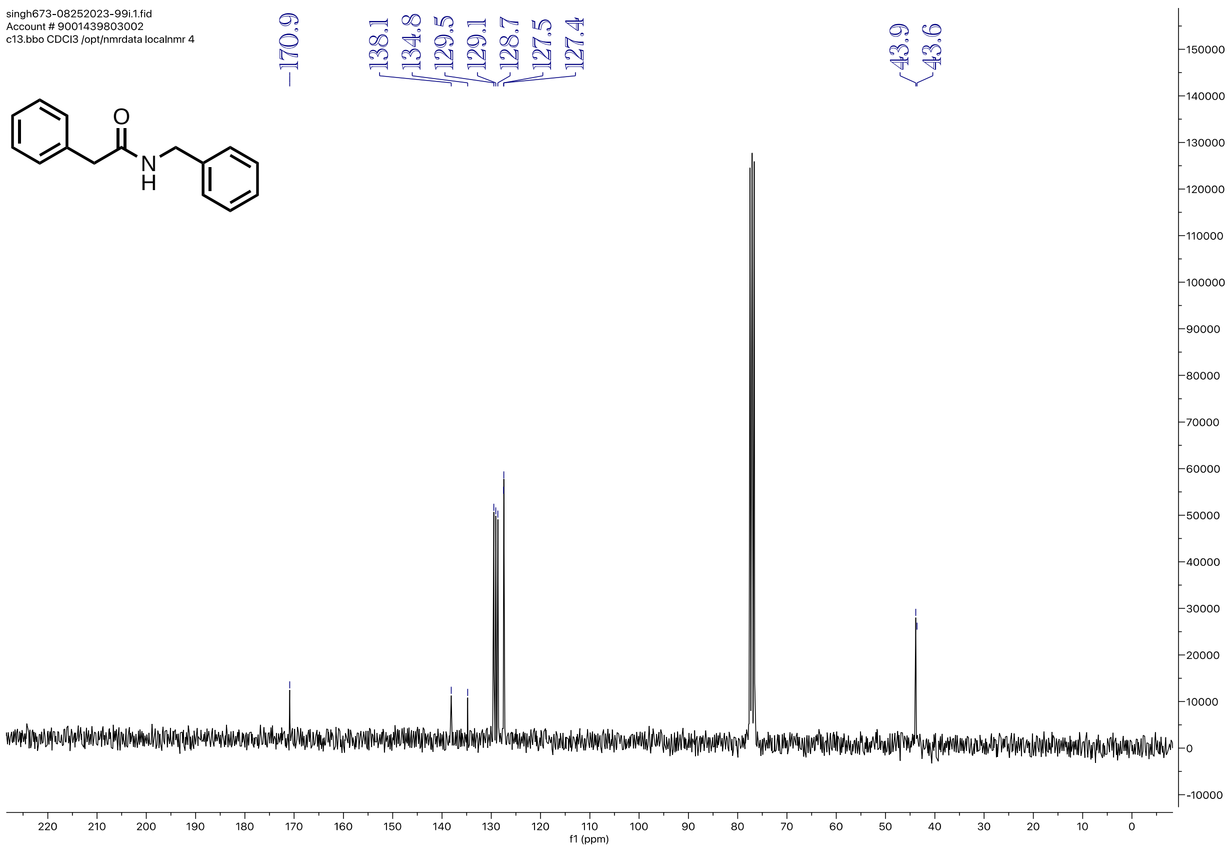

$^{13}C\{H\}$ NMR (75 MHz, Chloroform-*d*) *N*-benzyl-2-phenylacetamide (**4t**)

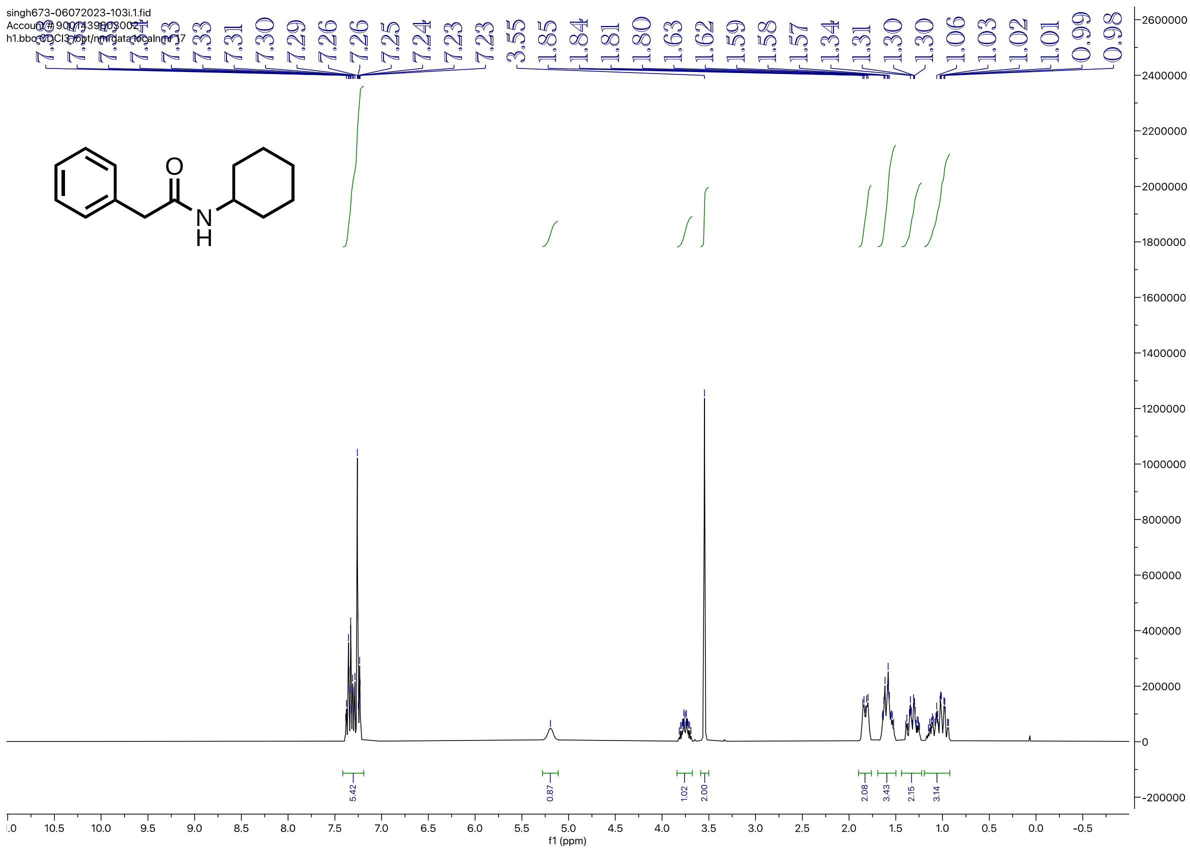

^{1}H NMR (300 MHz, Chloroform-*d*) *N*-cyclohexyl-2-phenylacetamide (**4u**)

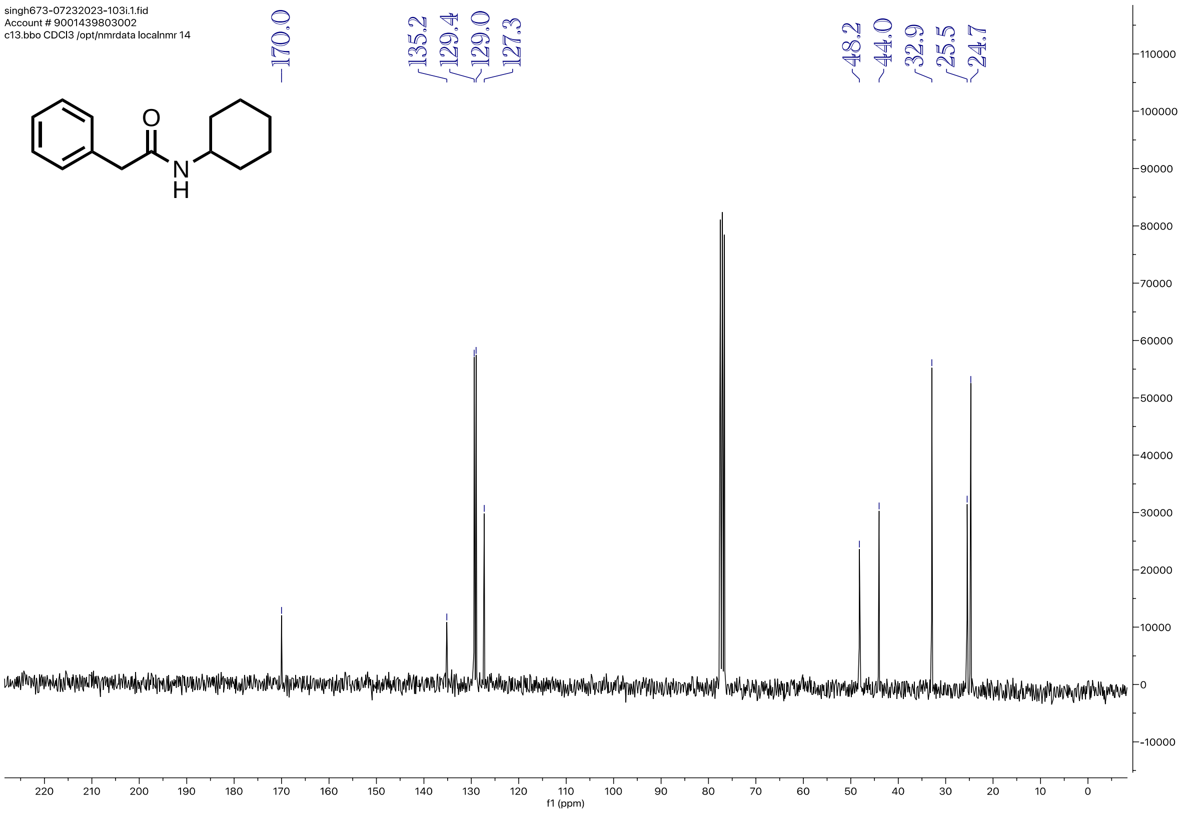

^{13}C{H} NMR (75 MHz, Chloroform-*d*) *N*-cyclohexyl-2-phenylacetamide (**4u**)

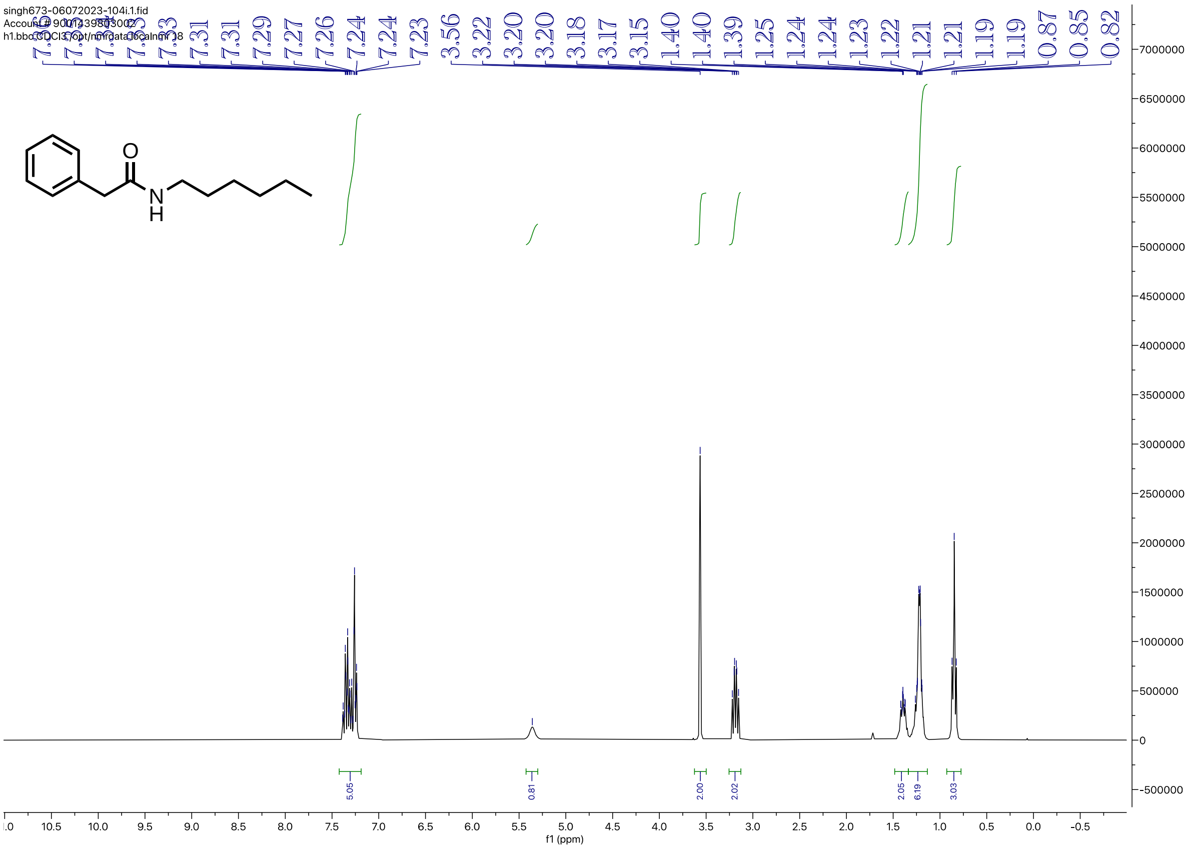

^{1}H NMR (300 MHz, Chloroform-*d*) *N*-hexyl-2-phenylacetamide (**4v**)

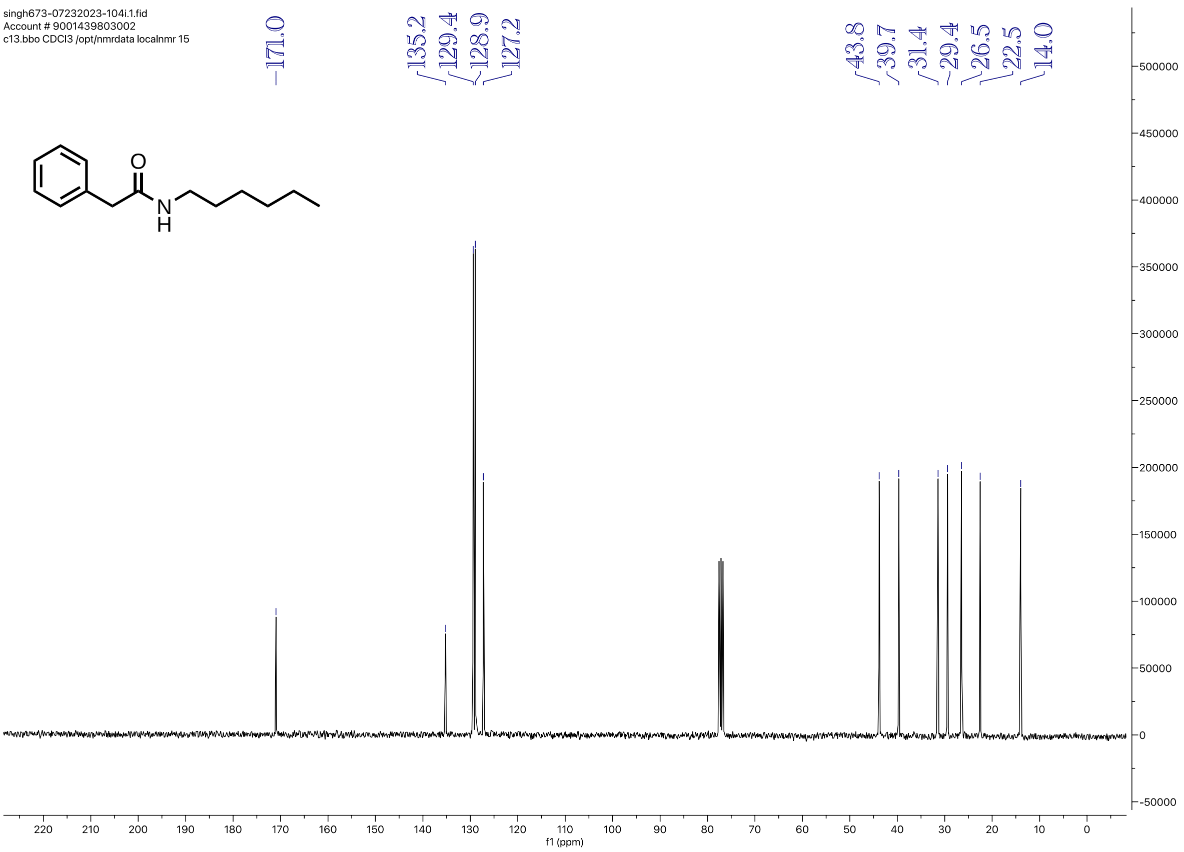

^{13}C{H} NMR (75 MHz, Chloroform-*d*) *N*-hexyl-2-phenylacetamide (**4v**)

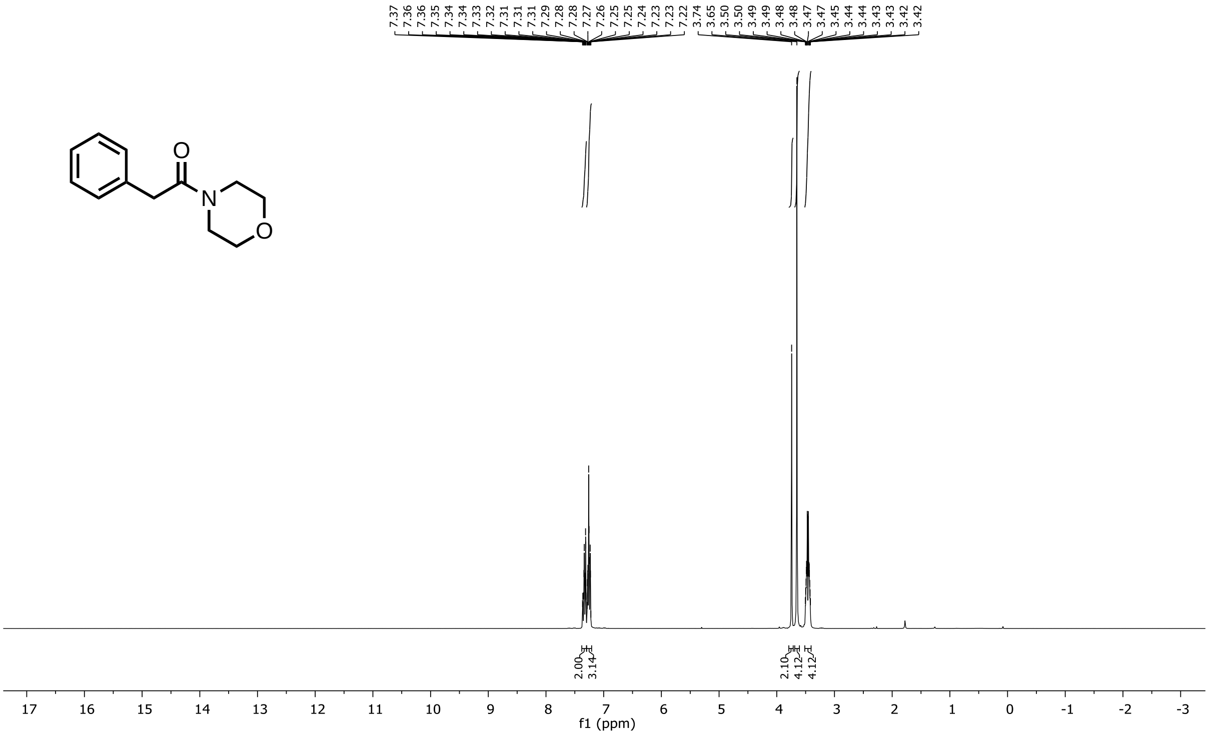

^{1}H NMR (300 MHz, Chloroform-*d*) 1-morpholino-2-phenylethan-1-one (**4w**)

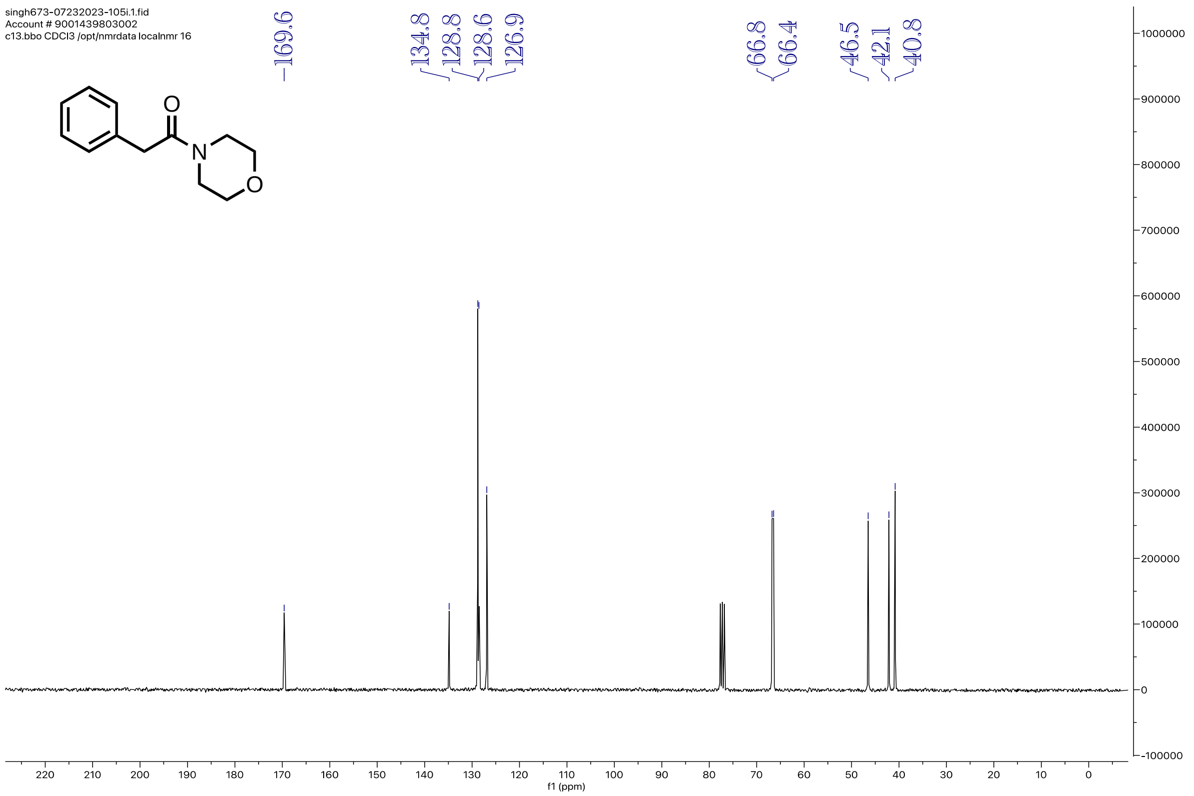

$^{13}C\{H\}$ NMR (75 MHz, Chloroform-*d*) 1-morpholino-2-phenylethan-1-one (**4w**)

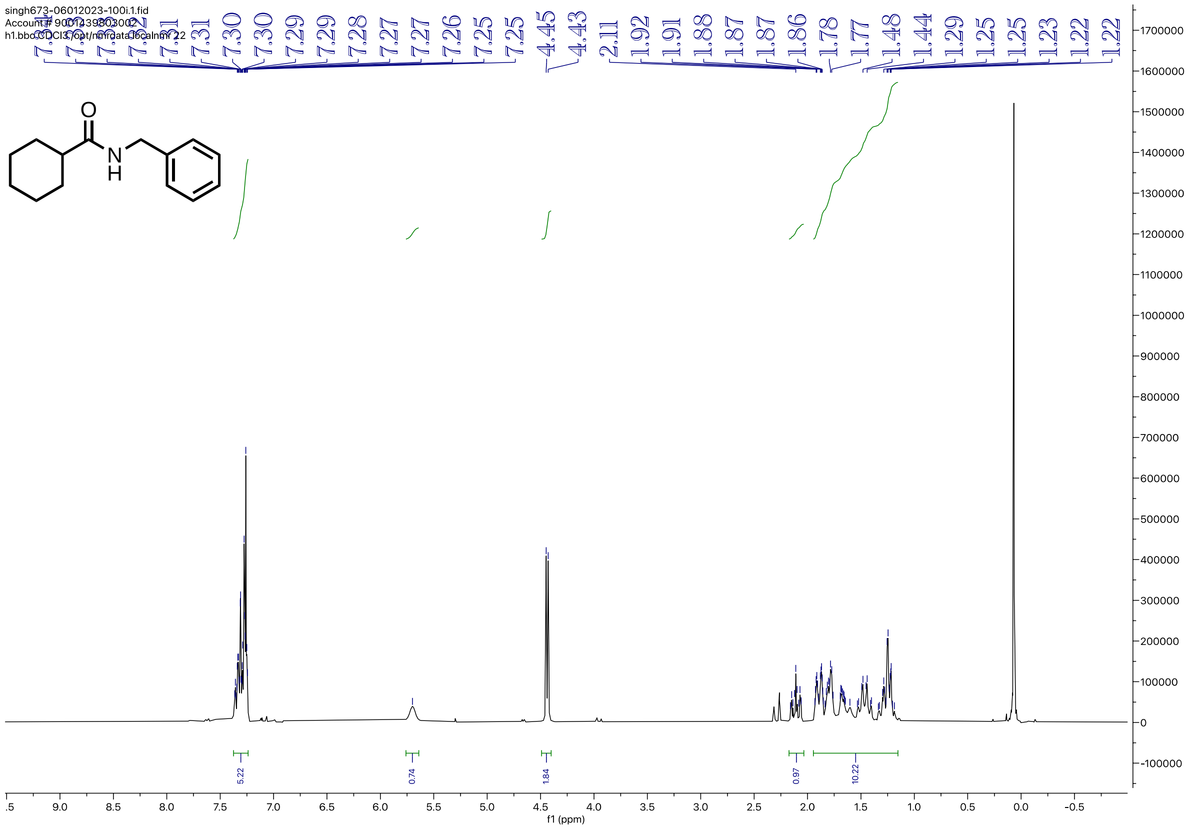

^{1}H NMR (300 MHz, Chloroform-*d*) 1-morpholino-2-phenylethan-1-one (**4x**)

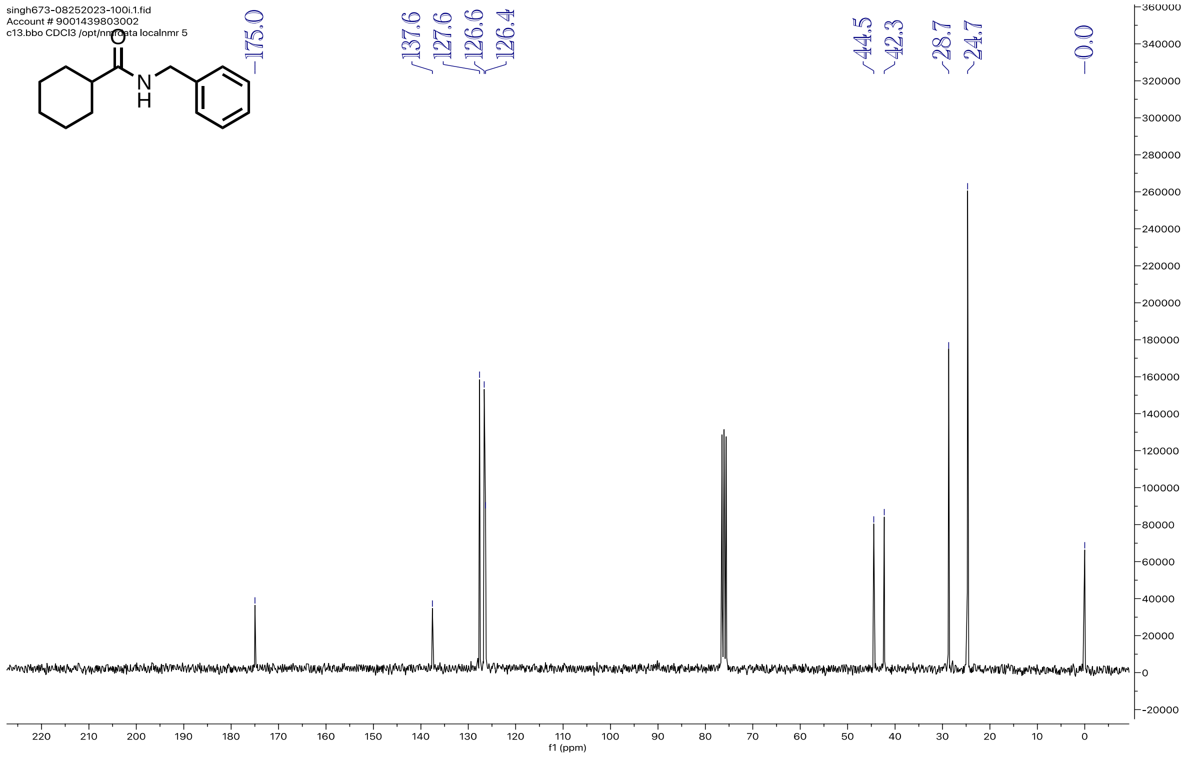

$^{13}C\{H\}$ NMR (75 MHz, Chloroform-*d*) 1-morpholino-2-phenylethan-1-one (**4x**)

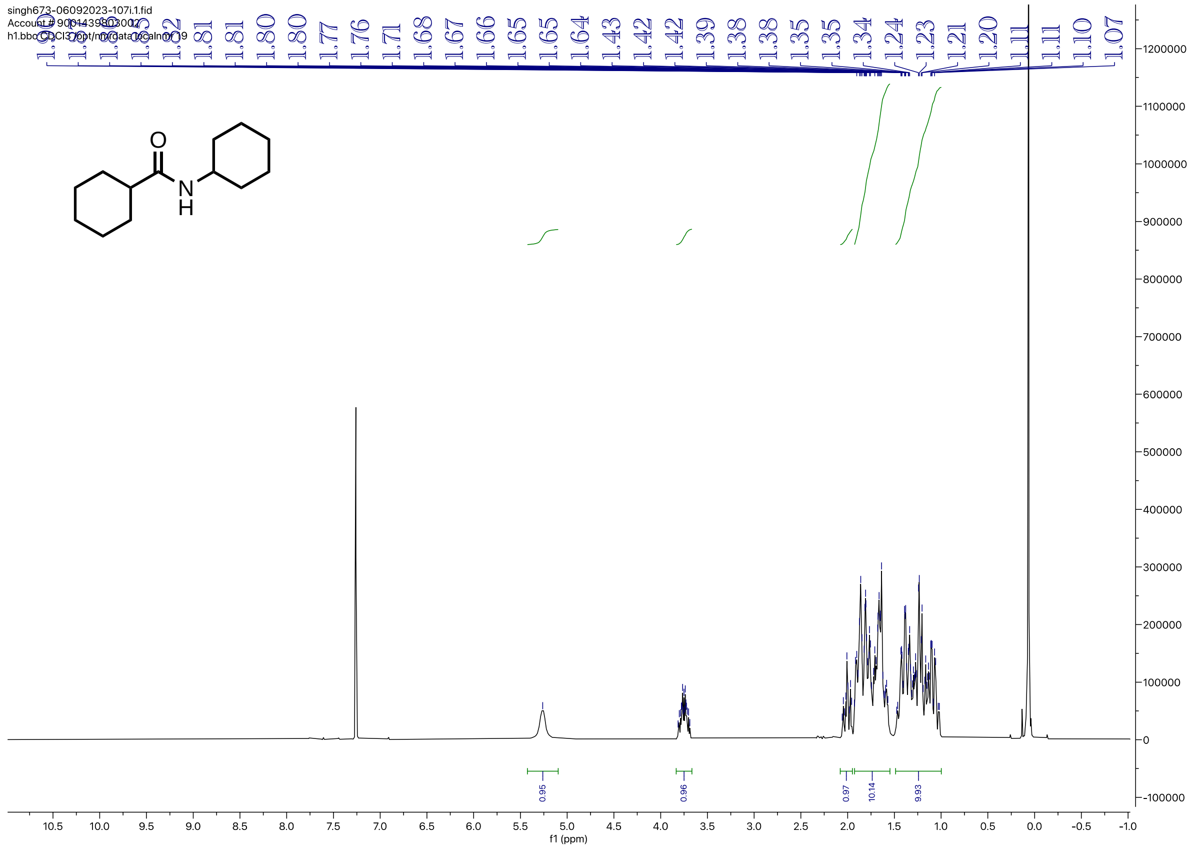

^{1}H NMR (300 MHz, Chloroform-*d*) *N*-cyclohexylcyclohexanecarboxamide (**4y**)

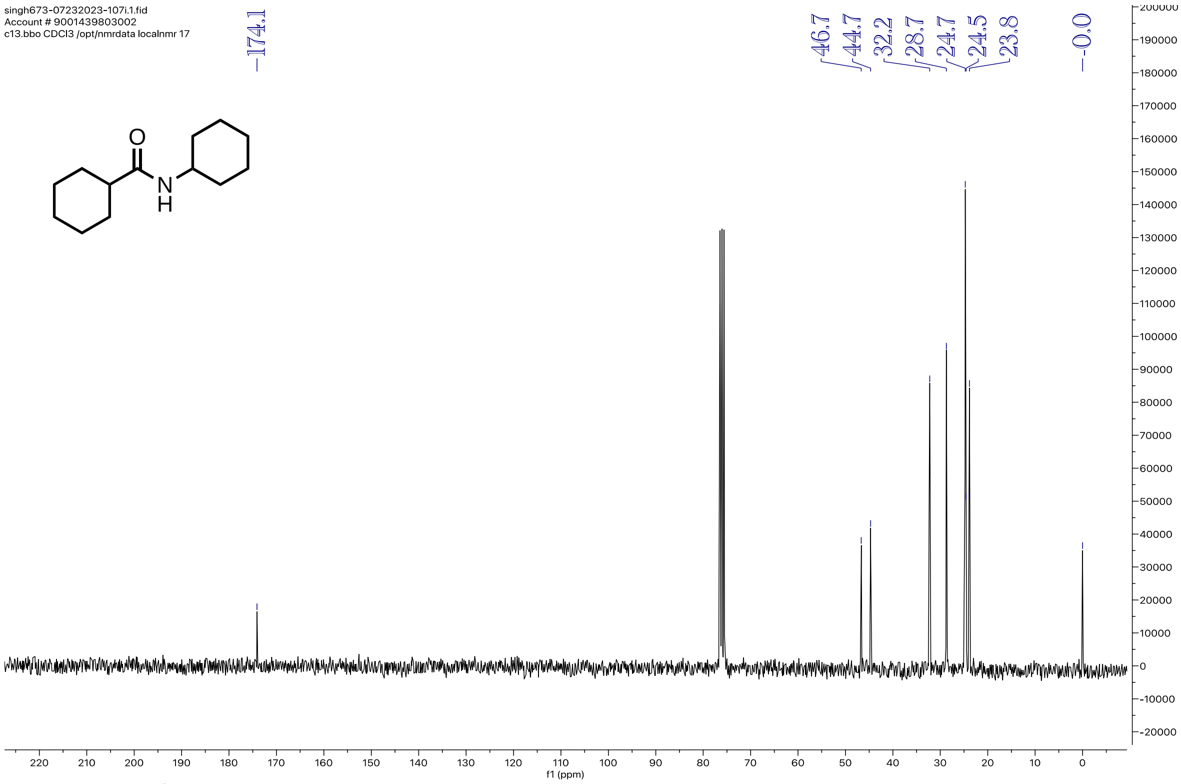

$^{13}C\{H\}$ NMR (75 MHz, Chloroform-*d*) *N*-cyclohexylcyclohexanecarboxamide (**4y**)

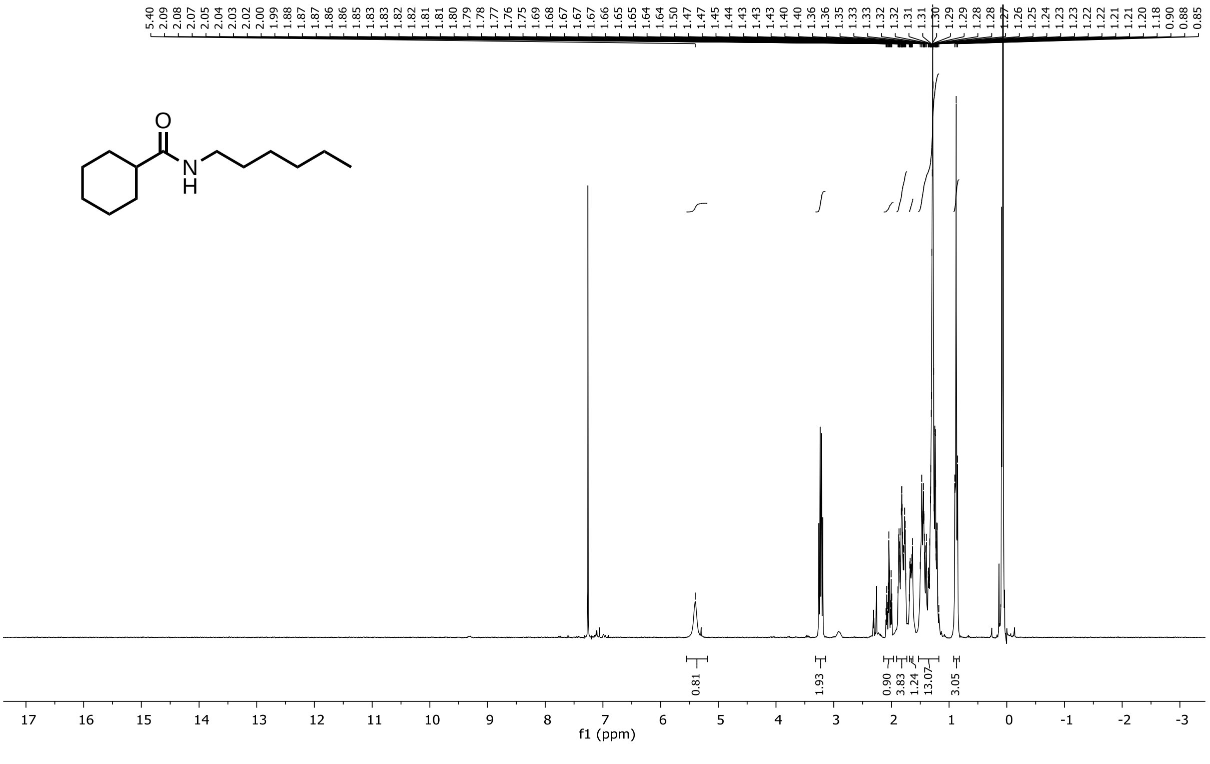

^{1}H NMR (300 MHz, Chloroform-*d*) *N*-hexylcyclohexanecarboxamide (**4z**)

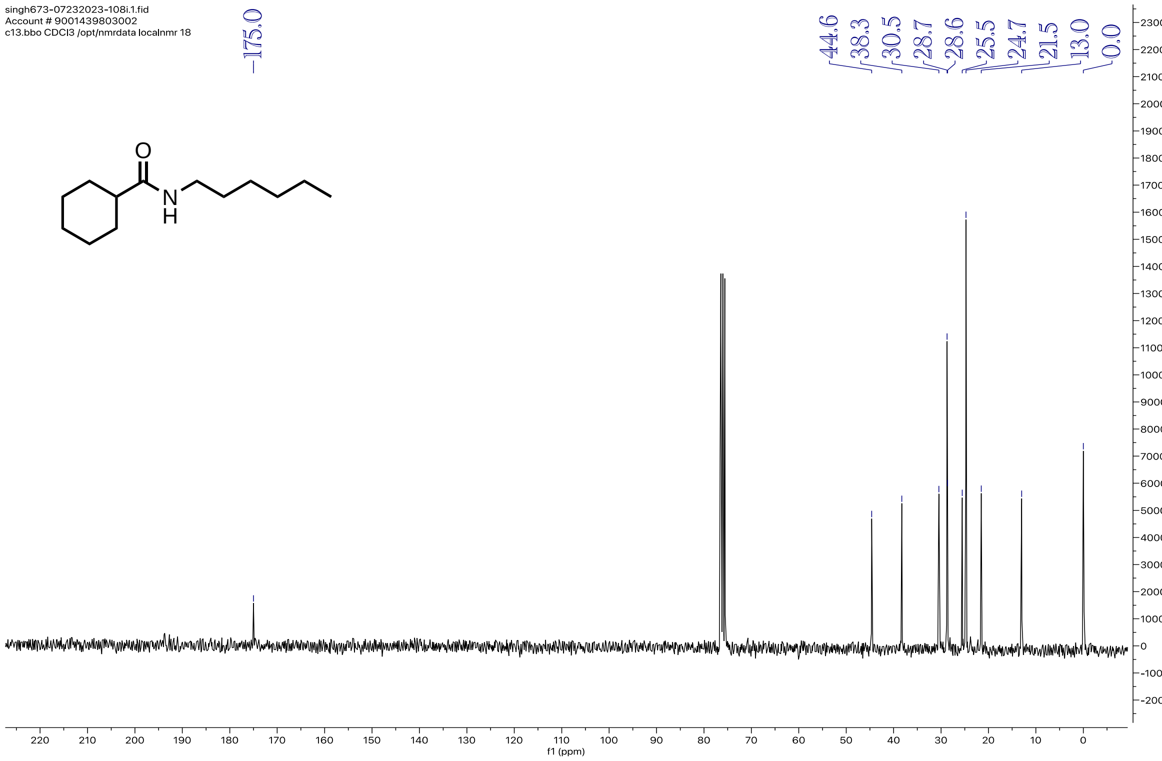

^{13}C{H} NMR (75 MHz, Chloroform-*d*) *N*-hexylcyclohexanecarboxamide (**4z**)

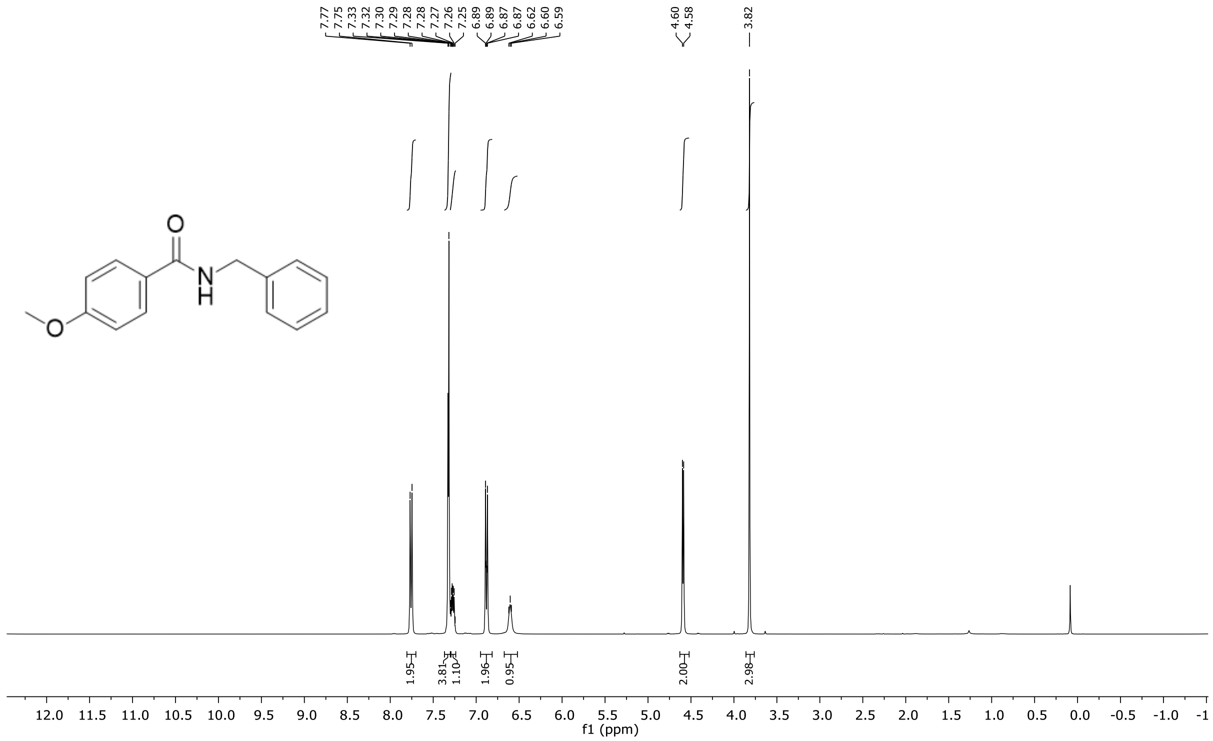

^{1}H NMR (400 MHz, Chloroform-*d*) *N*-benzyl-4-methoxybenzamide (**4aa**)

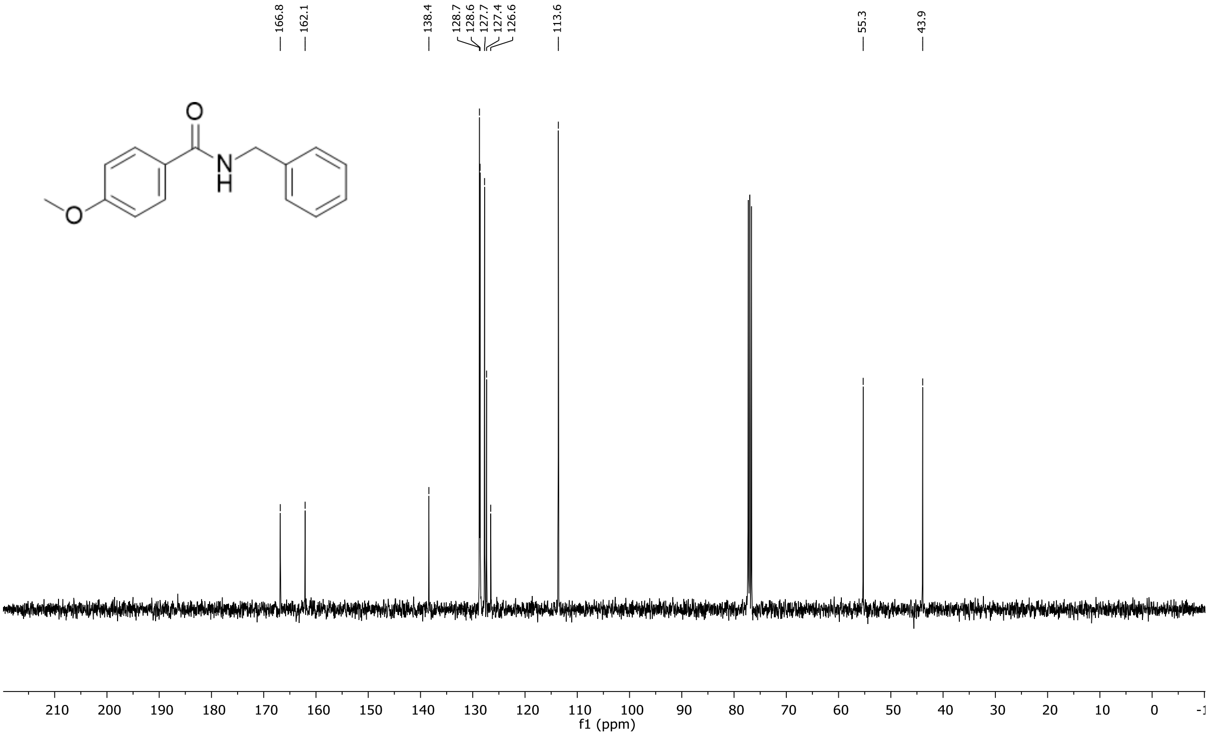

$^{13}C\{H\}$ NMR (101 MHz, Chloroform-*d*) *N*-benzyl-4-methoxybenzamide (**4aa**)

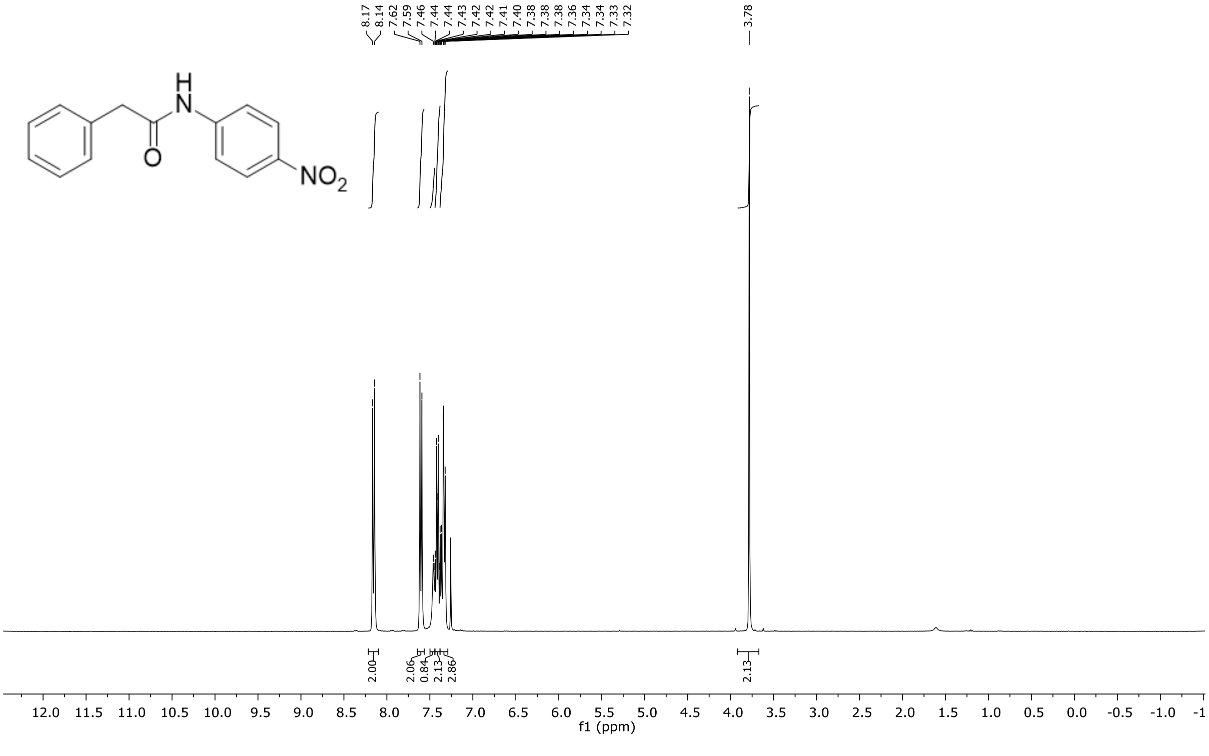

^{1}H NMR (400 MHz, Chloroform-*d*) *N*-(4-nitrophenyl)-2-phenylacetamide (**4ab**)

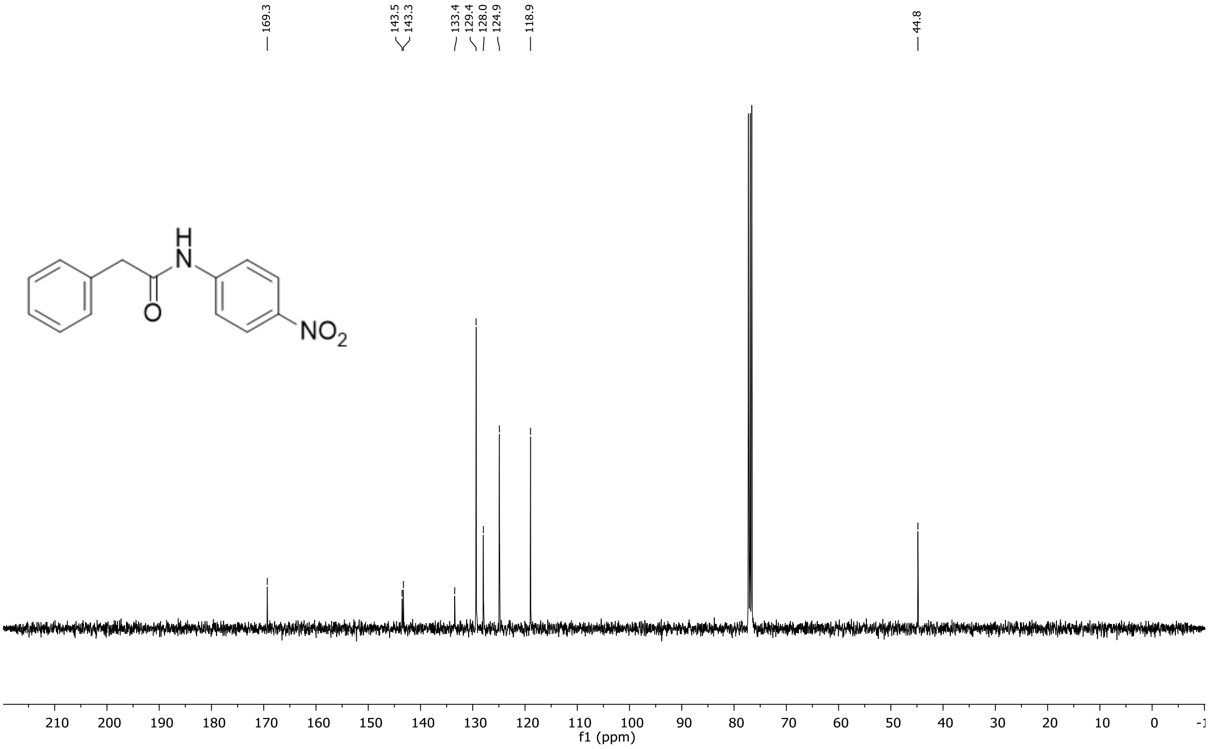

$^{13}C\{H\}$ NMR (101 MHz, Chloroform-*d*) *N*-(4-nitrophenyl)-2-phenylacetamide (**4ab**)
